# Inhibition of DPP-4 Attenuates Endotoxemia-Induced NLRC4 Inflammasome and Inflammation in Visceral Adipose Tissue of Mice Fed a High-Fat Diet †

**DOI:** 10.3390/biom15030333

**Published:** 2025-02-25

**Authors:** Francesca Bianchi, Paola Roccabianca, Elena Vianello, Guendalina Gentile, Lucia La Sala, Francesco Bandera, Lorenza Tacchini, Riccardo Zoia, Massimiliano M. Corsi Romanelli, Elena Dozio

**Affiliations:** 1 Dipartimento di Scienze Biomediche per la Salute, Università degli Studi di Milano, 20133 Milan, Italy; francesca.bianchi1@unimi.it (F.B.); elena.vianello@unimi.it (E.V.); guendalina.gentile@unimi.it (G.G.); lucia.lasala@unimi.it (L.L.S.); francesco.bandera@unimi.it (F.B.); lorenza.tacchini@unimi.it (L.T.); riccardo.zoia@unimi.it (R.Z.); mmcorsi@unimi.it (M.M.C.R.); 2 Laboratorio di Morfologia Umana Applicata, IRCCS Policlinico San Donato, 20097 San Donato Milanese, Italy; 3 Dipartimento di Medicina Veterinaria e Scienze Animali, Università degli Studi di Milano, 26900 Lodi, Italy; paola.roccabianca@unimi.it; 4 Laboratorio Sperimentale Ricerche Biomarcatori di Danno d’Organo, IRCCS Istituto Auxologico Italiano, 20149 Milan, Italy; 5 IRCCS MultiMedica, 20138 Milan, Italy; 6 Dipartimento di Patologia Clinica e Sperimentale, IRCCS Istituto Auxologico, 20149 Milan, Italy

**Keywords:** adipocytes, DPP-4, endotoxemia, fat, high-fat diet, inflammation, inflammasomes, linagliptin, macrophage phenotype, NLRC-4 inflammasome

## Abstract

Inflammasomes are protein complexes that trigger pro-inflammatory responses and promote many diseases, including adipose tissue dysfunction. Linagliptin (L), a DPP-4 inhibitor used for type 2 diabetes therapy, has putative anti-inflammatory effects. This work explores L effects on inflammasome regulation, inflammation, and adipose tissue dysfunction in obese mice. Male C57BL/6N mice were fed a normal chow (NC) diet, high-fat (HF) diet, or HF diet with L (HFL) for 15 weeks. Gene expression and histological examinations were performed on visceral (VAT) and subcutaneous (SAT) adipose tissue samples. Biomarkers were quantified on sera. Murine macrophages were utilized for in vitro analyses. L decreased HF-induced endotoxemia and circulating inflammatory indicators. Despite having no effect on body weight, L reduced VAT inflammation by decreasing endotoxemia-induced *NLRC4* inflammasome, inflammation severity, and fat cell hypertrophy. Although SAT response differed from VAT, inflammation was slightly reduced in this tissue too. In vitro, L modulated inflammation by directly reducing the pro-inflammatory macrophage phenotype. In obesity, increased *NLRC4* inflammasome expression links endotoxemia and VAT inflammation. L protected against endotoxemia, maybe by affecting gut permeability and VAT responses. The decreased polarization of macrophages toward a pro-inflammatory phenotype and the reduction in adipocyte hypertrophy are involved in the response to L.

## 1. Introduction

The low-grade inflammation associated to obesity, mainly visceral obesity, contributes to the onset and progression of several obesity-related morbidities, such as atherosclerosis, metabolic syndrome, insulin resistance, type 2 diabetes mellitus (T2DM), and non-alcoholic fatty liver disease [1]. In response to excessive food intake, visceral adipose tissue (VAT) expands mainly through adipocyte hypertrophy. When the maximal adipocyte volume is reached, the rate of cell death increases and “crown-like” structures, mostly composed by M1-polarized macrophages, appear around dying adipocytes [2]. Lipids and other small molecules, including lipid-derived metabolites, adipokines, chemokines, and cytokines, are released by dying adipocytes and infiltrating immune cells. All together, they sustain a chronic inflammatory response that not only impairs VAT metabolism but has additional detrimental effects on many organs [3,4,5,6,7,8,9].

Inflammasomes are multimeric protein complexes that assemble in the cell cytoplasm in response to stressors and activate pro-inflammatory caspases, which in turn induce the synthesis of cytokines and promote pyroptotic cell death. Structurally, most inflammasomes are composed of a sensor protein (PRR—unique pattern recognition receptor), an adaptor protein (ASC—apoptosis-associated speck-like protein), and an inactive procaspase [10]. Inflammasomes have been linked to the onset and progression of different inflammatory diseases, including obesity-related complications, and their modulation is under investigation as a promising therapeutic target [11,12]. Depending on the PRR sensor protein, specific inflammasome cascades can be activated [13]. Proteins of the nucleotide-binding and oligomerization domain (NOD)-like receptor (NLR) family are a group of PRR, and some of them, including NLRP3, NLRP6 NLRC1, NLRC2, NLRC5, and NLRP12, promote inflammation in obesity [14,15,16].

In obese subjects, both VAT and subcutaneous adipose tissue (SAT) express higher levels of the enzyme dipeptidyl peptidase-4 (DPP-4) than lean individuals. As DPP-4 expression rises, local inflammation, adipocyte size, and extracellular matrix deposition increase both in vivo and in vitro [17,18]. Linagliptin (L) is a DPP-4 inhibitor used in the clinical practice for the treatment of T2DM. One study indicated that L attenuates obesity-associated inflammation by decreasing pro-inflammatory macrophages infiltrating adipose tissue [19]. According to pre-clinical studies, L seems to protect against inflammation and thrombosis, regardless of its glucose-lowering effects [20,21]. In T2DM patients, treatment with L decreases markers of systemic inflammation and improves endothelial and microvascular functions [22]. In hemodialysis patients with T2DM, L monotherapy decreases inflammatory markers such as prostaglandin E2 and interleukin (IL)-6 [23]. Finally, the RELEASE study further suggests a favorable effect of L on arterial stiffness in T2DM [24].

Since obesity can affect adipose tissue metabolism by modulating inflammasomes and L seems to exert additional anti-inflammatory actions regardless of its glucose-lowering effects, in this study, we used a mouse model of diet-induced obesity to explore the effect of L on high-fat (HF) diet-induced inflammasomes and immune cell infiltration in VAT and SAT. The effect of L in modulating immune activation was further tested in an in vitro model of macrophages. The final aim is to highlight the off-target effects of L as a modulator of inflammasomes in the adipose tissue of obese mice.

## 2. Materials and Methods

### 2.1. Animal Model

Twenty-one six-week-old male C57BL/6N mice (Charles River Laboratories, Calco, Italy) were divided into 3 groups and fed for 15 weeks as follows: (1) normal chow diet with 10% calories from fat (NC, *n* = 8), (2) high-fat diet with 60% calories from fat (HF, n = 5), and (3) HF with L (120 μg/die, HFL, n = 8) (Charles River Laboratories). The mice were housed at constant room temperature (22 ± 2 °C) and humidity (60 ± 5%) with a light-dark cycle of 12 h each and water at libitum. Body weight was measured before diet administration (week 0–W0), after 7 weeks (W7), and at the day of sacrifice (week 15–W15) using a precision scale. At the age of 21 W, mice were sacrificed by exposure to atmosphere saturation of carbon dioxide. VAT and SAT were immediately snap-frozen in liquid nitrogen and stored at −80° until analyses, or fixed in 10% buffered formalin, routinely processed, and paraffin-embedded. Serum samples were obtained from blood taken from the left ventricle and stored at −80°C. The Italian Ministry of Health approved all animal procedures, which were performed in accordance with the Guidelines for the Care and Use of Laboratory Animals (authorization 467/2022-PR). L was kindly supplied by Boehringer Ingelheim, Milan, Italy.

### 2.2. Total RNA Extraction from Tissues and Reverse Transcription

Disruption and homogenization of tissue samples were performed with the TissueLyser II equipment (QIAGEN, Milan, Italy) through high-speed shaking in plastic tubes with stainless steel beads. Total RNA was then isolated using the RNeasy Lipid Tissue Mini Kit (QIAGEN), according to the manufacturer’s instructions. RNA concentration was quantified with NanoDrop (Thermo Fisher Scientific, Monza, Milan, Italy). RNA samples (0.5 μg) were first treated with a genomic DNA elimination step (5 min/42 °C and kept on ice at least 1 min) and then reversely transcribed using the RT^2^ First Strand Kit (15 min/42 °C and 5 min/95 °C) (QIAGEN). Samples were stored at −20 °C until real-time polymerase chain reaction (qPCR).

### 2.3. RT^2^ Profiler PCR Arrays and Single Gene Quantification

RT^2^ Profiler PCR Arrays allowed the detection of 84 key gene transcripts related to mouse inflammasomes (PAMM-097ZF, QIAGEN). Each cDNA sample was diluted with nuclease-free water and mixed with the RT^2^ SYBR green Mastermix (QIAGEN). Twenty-five μL of the same experimental mixture was automatically added to each well of the array (one array for each cDNA) using the QIAgility^®^ equipment (QIAGEN). qPCR was performed by the QIAquant (QIAGEN) and consisted of an initial activation of the Hot-start DNA Taq polymerase at 95 °C/10 min, followed by 40 cycles of 95 °C/15 sec and 60 °C/1 min. Dissociation curves were then performed to verify the specificity of the amplicons using the default melting curve program of the instrument. Data were analyzed using the RT2 Profiler PCR Array Data Analysis Web Portal (QIAGEN). A list of all transcripts included in the array panel is included in Supplementary File S1. A set of additional gene transcripts, including *CD68* (Cluster of Differentiation 68), *ITAGX* (Integrin Subunit Alpha X), *MRC1* (Mannose Receptor C-Type 1), *IL-10* (Interelukin-10), *PPIA* (Peptidylprolyl Isomerase A), *SLC2A4* (Solute Carrier Family 2 Member 4), *NOS2* (Nitric Oxide Synthase 2), and *ARG2* (Arginase 2), was quantified using specific gene primers (catalog 330001-RT^2^ PCR Primer Set, QIAGEN) and with the same reagents, equipment, and conditions previously described.

### 2.4. Analysis of RT2 Profiler PCR Arrays

Each array includes 5 housekeeping genes for normalization, 1 genomic DNA control, 3 reverse transcription controls, and 3 positive PCR controls. The same threshold was applied to all arrays for data analysis. A Ct (cycle threshold) > 33 was considered a negative call. Ct for genomic DNA controls > 33 and Ct of 20 ± 2 for the positive PCR controls confirmed the lack of DNA contamination and efficient PCR amplification, respectively. Normalization of data expression can be performed using one of the housekeeping genes or any other of the 84 genes on the condition that the Ct value of the gene used for normalization does not differ more than 1.5 cycle across arrays. The RT2 Profiler PCR Array Data Analysis Web Portal was used for data analysis. The reference genes were automatically chosen by the software. Normalization was then performed by calculating the ΔCt for each gene. The fold change was then obtained based on the ΔΔCt method. For fold-change values greater than 1, the results were reported as fold upregulation. For fold-change values less than 1, the negative inverse of the results was reported as fold downregulation. Genes that showed a fold change > 1.7 or <−1.7 were shown [25]. The *p* values are calculated based on a Student’s *t*-test of the replicate 2^(-^ΔCt^) values for each gene in the control group and treatment groups, and *p* values less than 0.05 are indicated. The ΔΔCt method was also applied for calculating the fold change of gene transcripts not included in the array. PPIA was utilized as the housekeeping gene in this set of assays.

### 2.5. Lipopolysaccharide (LPS) Binding Protein (LBP) Quantification

LBP was quantified by the Mouse LBP SimpleStep ELISA^®^ Kit (LPS Binding Protein) by Abcam (Cambridge, UK) according to manufacturer’s instructions. Briefly, 50 µL of standard and serum samples was mixed with 50 µL of an antibody mix cocktail and incubated for 1 h at room temperature on a plate shaker. After washing, 100 µL of a TMB Development Solution was added to each well and incubated for 10 min in the dark on a plate shaker. The reaction was stopped by adding 100 µL of stop solution. The absorbance was then read at 450 nm and concentration was determined by interpolating the blank control subtracted-absorbance values against the standard curve.

### 2.6. Tissue Preparation, Histological Examination, and Scoring

#### 2.6.1. Microscopic Morphologic Analysis

Five μm thick sections of VAT and SAT were stained with hematoxylin–eosin and visualized using a Leica DM1000 microscope (Leica Microsystems, Milan, Italy). Adipocyte morphology, nuclear morphology, and presence of inflammatory cells (lymphocytes, plasma cells, macrophages, and neutrophils) were qualitatively assessed. Inflammation was scored semi-quantitatively as follows: score 0, no inflammation; score 1, ≤5 inflammatory foci with mild accumulation of cells (≤ of 20 inflammatory cells per accumulation); score 2, >5 inflammatory foci ≤ 20, with a moderate accumulation of inflammatory cells (>20 cells ≤50 per accumulation); score 3, >20 diffuse inflammatory foci or severe accumulations (>50 cells per accumulation).

#### 2.6.2. Adipocyte Counts

For adipocyte counts, single-field images from the same sections of fat used to assess morphology were acquired. For each case, an image was collected at 400× magnification, choosing the area of greatest cell expansion. To further standardize image acquisition, adipocytes closest to the small intestinal serosa were obtained for VAT. For SAT, images were taken from adipocytes adjacent to skeletal muscle. Images were acquired with a Leica ICC50 W camera(Leica, Milan, Italy) mounted on a Leica DM 100 microscope using a 40×/0.65 objective and 10× eyepiece including as many adipocytes with complete membranes as possible. All images corresponded to the same area (width [pixels]: 2592, height [pixels]: 1944). Adipocytes were counted manually by including only adipocytes where all 4 membranes were present and excluding incomplete adipocytes. Manual counts were performed even though they are time-consuming because they are considered more accurate [26].

#### 2.6.3. Image Analysis

For digital image analysis, images were acquired at 200× magnification using a Leica ICC50 W camera mounted on a Leica DM 100 microscope using a 20×/0.40 objective and 10× eyepiece. All images were collected as Jpeg format and corresponded to the same area (width [pixels]: 2592, height [pixels]: 1944). Area of adipocytes and their equivalent diameter were collected for all adipocytes using image J software with the Adiposoft plugin automated analysis utilizing the following settings: auto mode, exclude on edges, pixel output units, analyzing a directory comprising all the images belonging to one group [27,28]. Data per group were obtained as excel spreadsheet and mean, median, and standard deviation. Image metadata were width [voxel]: 2592, height [voxel]: 1944, number of channels: 4, number of slices: 1, number of frames: 1, voxel width: 1.0, voxel height: 1.0, voxel depth: 1.0, frame interval: 0.0, spatial unit: pixel, and temporal unit: s.

### 2.7. Cells and Cell Culture

Immortalized murine macrophage (RAW 264.7, from ATCC, LGC Standards S.r.l.—Sesto San Giovanni, Italy) cell line was routinely maintained at 37 °C in 5% CO_2_ atmosphere in Dulbecco’s Modified Eagle Medium (DMEM) (Gibco, Thermofisher Scientific, Milan, Italy) supplemented with 10% fetal bovine serum (Gibco). Cells were seeded at 5 × 10^5^ cells/well in 6-well plates (Costar, Corning Incorporated, Sigma-Aldrich, Milan, Italy). Untreated RAW 264.7 mouse macrophages, considered as M0 macrophages, were stimulated with IFN-γ (5 ng/mL) and LPS 10 ng/mL to induce M1 phenotype, as previously described [29]. L was added to the culture medium at 100 nM or 500 nM final concentration for 6 or 24 h. RNA was isolated from harvested cells using QIAzol (QIAGEN) following the manufacturer’s instructions, as previously described [29,30]. Quantification of RNA concentration, reverse transcription, and gene amplification were performed as indicated in the previous sections.

### 2.8. Luminex Assay

Chemokine (C-C motif) ligand-5 (CCL-5), interleukin-10 (IL-10), interleukin-1 β (IL-1β), tumor necrosis factor α (TNFα), chemokine (C-C motif) ligand-7 (CCL-7), interferon γ (INFγ), interleukin-33 (IL-33), chemokine (C-C motif) ligand-11 (CCL-11), DPP-4, and interleukin-17A (IL-17A) serum levels were quantified by the Mouse Magnetic Luminex Discovery Assay kit (R&D System, Catalog Number LXSAMSM) and the Bio-Rad Bio-Plex (Bio-Rad Laboratories, Milan, Italy) according to manufacturer’s instructions, as also previously described [31,32]. Briefly, color-coded magnetic beads coupled with anti-cytokine antibodies were incubated with samples and standards. After washing, captured analytes were subsequently detected using a cocktail of biotinylated detection antibodies and a streptavidin–phycoerythrin conjugate. Luminex 200 instrument converted the mean fluorescence intensity of analytes into concentration (pg/mL). The standard curves and the lower limit of detection were as follows: CCL-5, 78.2–19,000 pg/mL, 19.1 pg/mL; IL-10, 12.8–3100 pg/mL, 8.20 pg/mL; IL-1β: 247–60,000 pg/mL, 41.8 pg/mL; TNFα: 2.88–700 pg/mL, 1.47 pg/mL; IL-33, 82.3–20,000, 57.1; CCL-11: 11.1–2700 pg/mL, 1.46 pg/mL; INFγ: 14.4–3500 pg/mL, 1.85 pg/mL; CCL-7: 6.17–500 pg/mL, 1.69 pg/mL; DPP-4: 364–88,500 pg/mL, 135 pg/mL; IL-17A: 49.4–12,000 pg/mL, 7.08 pg/mL.

### 2.9. Statistical Analysis

Analysis of genomic data can be found in the Section 2.4. Concerning other analyses, data are shown as mean ± SEM (standard error of the mean) or fold change. Comparisons between more than two groups were performed by one-way ANOVA and Bonferroni’s Multiple Comparison Test. The T test and Mann–Whitney test were used for two-group analyses. The GraphPad Prism 10.3.1 biochemical statistical package (GraphPad Software, San Diego, CA, USA) was used for data analyses. A *p*-value < 0.05 was considered significant.

## 3. Results

### 3.1. Effect of Linagliptin on Body Weight

**Figure 1:**
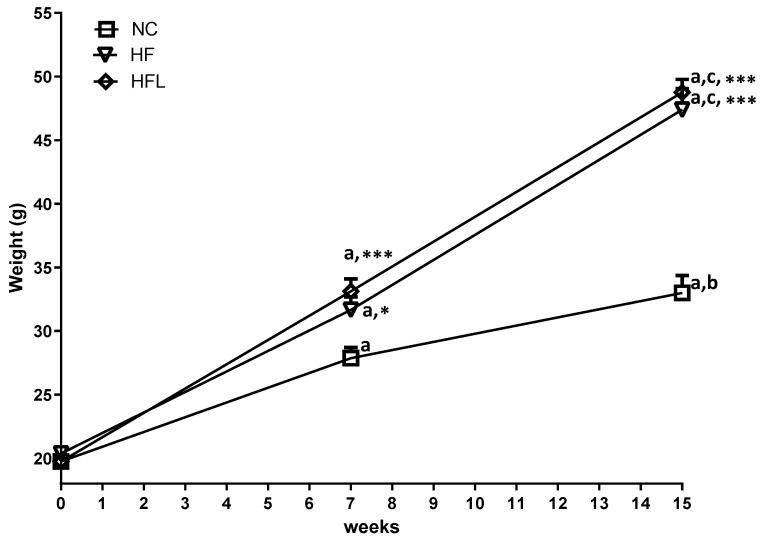
Effect of high-fat diet (HF) and high-fat diet combined with Linagliptin (HFL) on body weight. The figure shows the changes in body weight across the experimental weeks in each group. a, *p* < 0.001 vs. week 0; b, *p* < 0.01 vs. week 7; c, *p* < 0.001 vs. week 7; * *p* < 0.05 vs. NC, *** *p* < 0.001 vs. NC. NC, normal chow; HF, high fat; HFL, high-fat Linagliptin.

Each group gained weight from W0 to W15 (Figure 1). Significant differences were reported in all groups between W7 vs. W0 (*p* < 0.001), W15 vs. W0 (*p* < 0.001), and W15 vs. W7 (*p* < 0.01 for NC group and *p* < 0.001 for HF and HFL groups). Looking at inter-group body weight changes, HFL diet did not reduce body weight gain neither at W7 nor at W15 (*p* > 0.05, HFL vs. HF).

### 3.2. Effect of HF Diet and HF Combined with Linagliptin Diet (HFL) on Inflammasome-Related Transcriptome in VAT

To explore the potential protective effect of L on HF diet-induced inflammasomes in adipose tissue, we assessed the expression of a wide panel of gene transcripts related to mouse inflammasomes. Considering VAT, HF diet raised 17 gene transcripts and downregulated 2 gene transcripts, as compared to NC. Table 1 summarizes the names of HF-affected genes, their function, and fold regulation. Collectively, most of the upregulated genes belong to pro-inflammatory pathways, as expected, and to *NLRC4* inflammasome.

Upon HF diet, L affected the expression of 9 of the 19 genes selectively regulated by HF diet in VAT. Collectively, HFL increased the expression of 1 and mitigated the expression of 8 gene transcripts, thus promoting a pro-inflammatory effect. Table 2 summarizes the names of the HFL-affected genes, their roles, and fold regulation.

### 3.3. Effect of HF Diet and HF Combined with Linagliptin Diet (HFL) on Inflammasome-Related Transcriptome in SAT

In SAT, HF diet downregulated 20 gene transcripts and raised 5 gene transcripts as compared to NC, indicating a different activation of tissue inflammatory response compared to what was observed in VAT. Besides the activation of pro-inflammatory genes and the downregulation of different anti-inflammatory transcripts, a decrease in the expression of some pro-inflammatory genes can be further observed. This suggests a bidirectional response in SAT in which pro- and anti-inflammatory signals co-exist and may regulate each other. Table 3 summarizes the names of HF-affected genes, their function, and fold regulation. Contrary to what was observed in VAT, none of the genes modulated by the HF diet in SAT were affected by the addition of L, thus suggesting different responses of the two fat depots to HF and HFL diets.

### 3.4. Effect of HF and HF Combined with Linagliptin Diet (HFL) on LBP Serum Level

**Figure 2:**
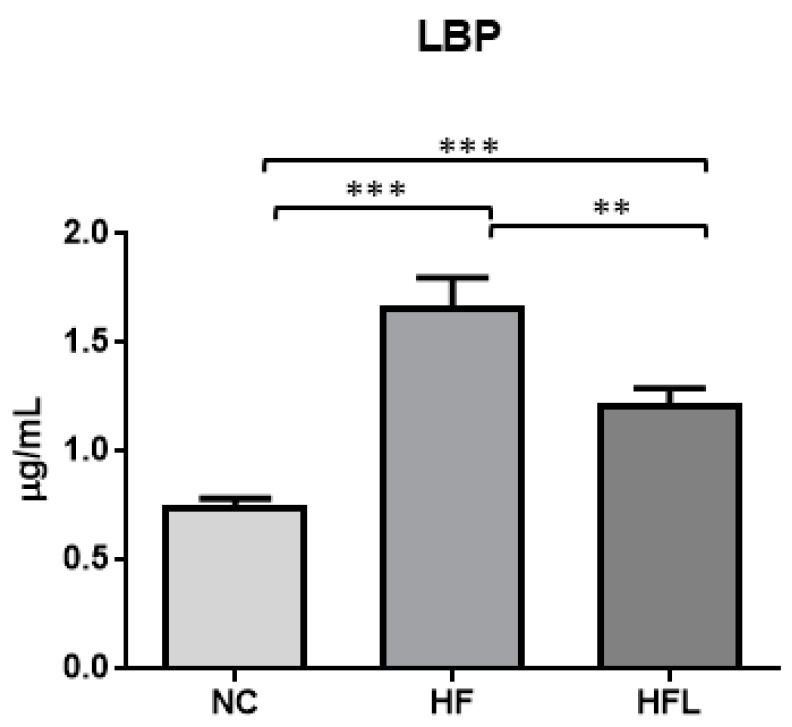
Effect of high-fat diet (HF) and high-fat diet combined with Linagliptin (HFL) on serum level of lipopolysaccharide binding protein (LBP). The figure shows the effect of HF diet and HFL diet on serum LBP concentration. ** *p* < 0.01, *** *p* < 0.001.

LBP was detected at low level in mice fed NC diet (0.74 ± 0.09 µg/mL). HF diet increased mean LBP serum concentration of about 2.3 times (1.67 ± SD 0.13 µg/mL, *p* < 0.001 vs. NC). The addition of L to HF diet significantly decreased LBP level of about 30% (1.22 ± SD 0.07 µg/mL, *p* < 0.01 vs. HF diet) (Figure 2).

### 3.5. Effect of HF Diet and HF Combined with Linagliptin Diet (HFL) Diet on VAT and SAT Immune Cells Infiltration, Adipocyte Area, and Equivalent Diameter

**Figure 3:**
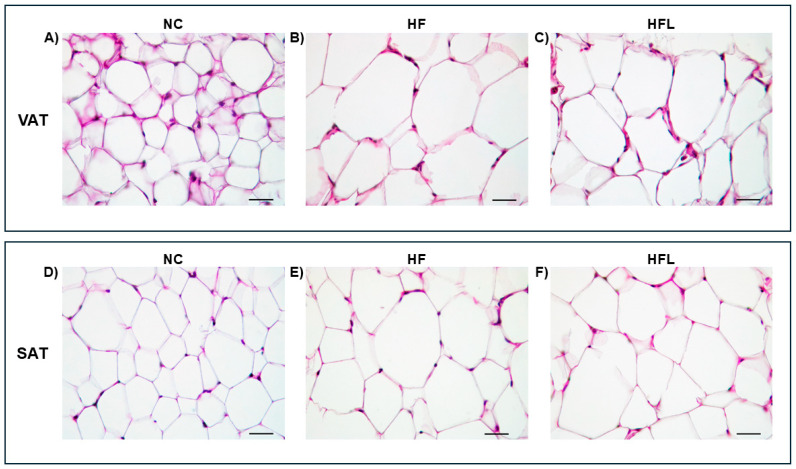
Histological analysis of visceral adipose tissue (VAT) and subcutaneous adipose tissue (SAT) of the three experimental groups (NC, normal chow; HF, high fat; FHL, high fat with Linagliptin). Microscopical morphology of VAT (panel **A**–**C**) and SAT (panel **D**–**F**) adipocytes stained with hematoxylin and eosin. VAT and SAT adipocytes of mice fed NC diet have a normal size with variably evident mostly plump nuclei (panel **A**,**D**), while those of mice fed HF diet are 2–4 times larger than NC group (panel **B**,**E**). In HFL group (panel **C**,**F**), adipocytes have similar morphology of those in HF group. Images are at 400× magnification. Bars = 50 microns.

The results of the morphological, semi-quantitative analysis of inflammation and adipocyte manual counts are provided and described in Supplementary Files S2–S8 and summarized in Figure 3. Concerning VAT, in the NC diet group, adipocyte numbers ranged from 23 to 37 and averaged 31.1, resulting in more numerous and smaller cells compared to adipocytes of HF and HFL groups. The number of HF adipocytes ranged from 8 to 15, averaging 11.4 cells. Adipocytes of the HFL group ranged from 6 to 15, averaging 10.8 cells. No major differences in adipocyte numbers were observed between HF and HFL groups (*p* > 0.05). Relative to SAT, NC adipocyte numbers ranged from 15 to 41 and averaged 29.3, resulting in more numerous and smaller cells compared to adipocytes of HF and HFL diet groups. HF adipocytes ranged from 8 to 19, averaging 12 cells. HFL adipocytes ranged from 8 to 18, averaging 11.1 adipocytes. No major differences in adipocyte numbers and sizes were observed between HF and HFL groups (*p* > 0.05).

**Figure 4:**
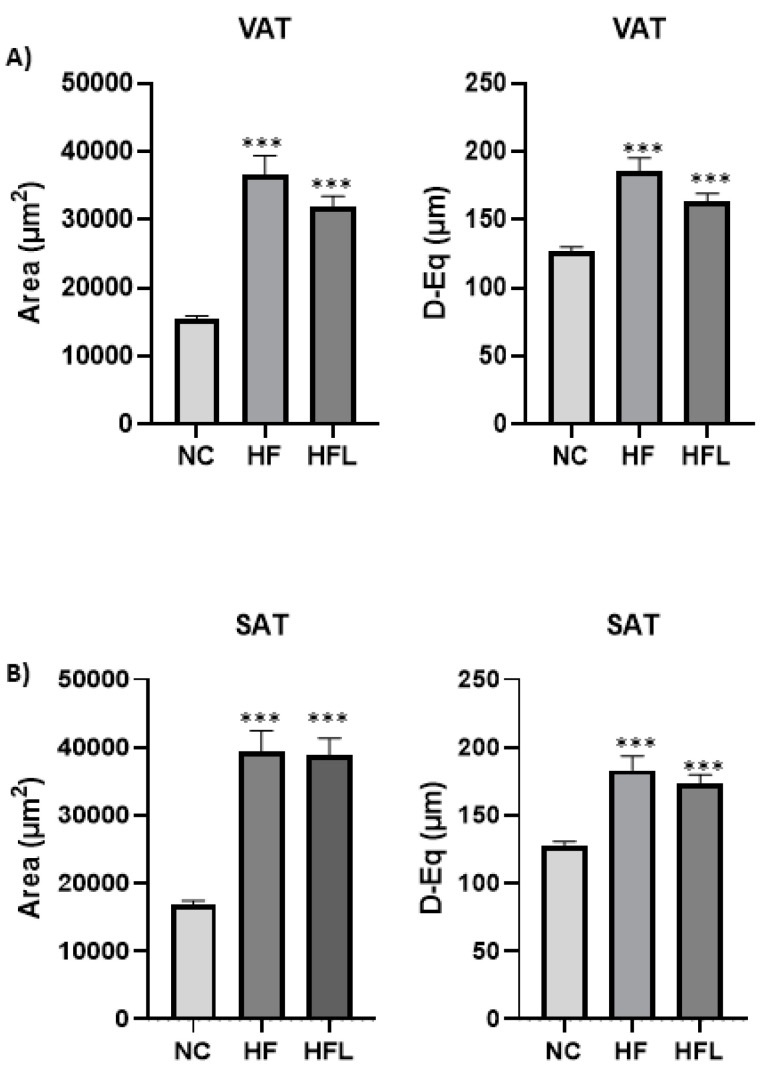
Effect of high-fat diet (HF) and high-fat diet combined with Linagliptin (HFL) on adipocyte equivalent diameter (D-Eq) and area. The figure shows the effect of HF diet and HFL diet on D-Eq and area of visceral (VAT, panel **A**) and subcutaneous (SAT, panel **B**) adipocytes. *** *p* < 0.001 vs. NC. In (panel **A**), the difference between HFL and HF was at limit of statistical significance both for area (*p* = 0.07) and D-Eq (*p* = 0.051).

The area and equivalent diameter of VAT (Figure 4, panel A) and SAT (Figure 4, panel B) adipocytes were compared between the three experimental groups. In both fat depots, HF diet and HFL diet significantly increased adipocyte area and equivalent diameter compared to NC diet (*p* < 0.001). In VAT (panel A), the mean area and equivalent diameter of HFL adipocytes were both lower than HF adipocytes (area: 31,848 ± 1594 μm^2^ vs. 36,625 ± 2758 μm^2^; diameter: 163.7 ± 5.43 μm vs. 185.3 ± 9.97 μm^2^) but at a limit of statistically significant (*p* = 0.051 for diameter and *p* = 0.074 for area).

### 3.6. Effect of HF Diet and HF Combined with Linagliptin Diet (HFL) on Macrophage-Polarization in VAT and in Murine Monocyte/Macrophage-like Cells

**Figure 5:**
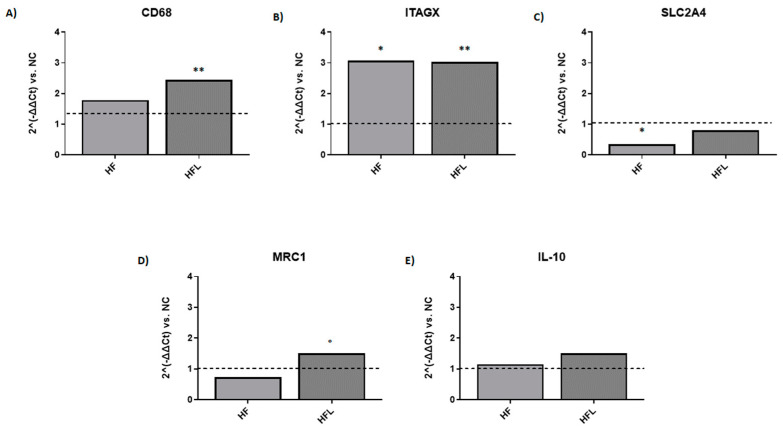
Effect of high-fat diet (HF) and high-fat diet combined with Linagliptin (HFL) on macrophage polarization in vivo. The figure shows the effect of HF and HFL diet on the expression of markers associated to M1- and M2-phenotype macrophage polarization in visceral adipose tissue. Data are expressed as 2^(-ΔΔCt) vs. NC (normal chow; dotted line). *CD68* (cluster differentiation 68, panel **A**) is an overall marker of macrophage, *ITAGX* (Integrin Subunit Alpha X, panel **B**) is a marker of M1-polarized macrophages, and *SLC2A4* encodes glucose transporter Type 4 (Solute Carrier Family 2 Member 4, panel **C**). *IL-10* (Interelukin-10, panel **D**) and *MRC1* (Mannose Receptor C-Type 1, panel **E**) are markers of M2-polarized macrophages. * *p* < 0.05 vs. NC, ** *p* < 0.01 vs. NC, ° *p* < 0.05 vs. HF.

To explore the effect of L on the HF diet regulation of immune cell infiltration in VAT, we assessed the phenotype of macrophages, the most relevant immune cells resident in adipose tissue, which can cross-interact with adipocytes. The HF diet induced the upregulation of the overall macrophage marker *CD68* (fold regulation +1.80 vs. NC, Figure 5, panel A), *ITAGX*, which encodes *CD11C*, a marker of M1-polarized macrophages (fold regulation +3.08 vs. NC, Figure 5, panel B) and promoted the downregulation of *SLC2A4*, the gene encoding the glucose transporter Type 4 (fold regulation −2.86 vs. NC, Figure 5, panel C). *MRC1*, which encodes the M2-polarized macrophage marker *CD206*, was slightly decreased by HF diet (fold regulation −1.40 vs. NC, Figure 5, panel D), whereas IL-10 was not affected (Figure 5, panel E).

The addition of L to the HF diet induced a slight increase in *CD68* expression (fold regulation +1.40 vs. HF) and promoted the expression of *SLC2A4*, which returned to expression level close to NC diet (fold regulation +2.30 vs. HF, Figure 5, panel C), and *MRC1* (fold regulation +2.06 vs. HF). The HF diet-induced expression of *ITAGX* was unaffected by L addition, whereas *IL-10* expression slightly increased (fold regulation +1.3 vs. HF). These results show a relative increase in macrophages infiltrating VAT upon HF diet, primarily with M1 phenotype. L supplementation improved local insulin sensitivity and promoted the expression of transcript related to M2 phenotype.

**Figure 6:**
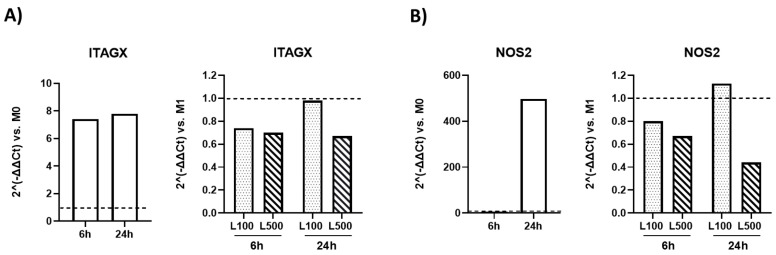
Effect Linagliptin (L) on macrophage polarization in vitro. The figure shows the effects of two L doses (100 and 500 nM) on the expression of markers associated to M1-phenotype macrophage polarization in vitro at 6 h and 24 h. Data are expressed as 2^(-ΔΔCt) vs. M0 and M1-polarized macrophages (dotted lines). ITAGX (Integrin Subunit Alpha X, panel **A**) and NOS2 (Nitric Oxide Synthase 2, panel **B**) are markers of M1-polarized macrophages.

To confirm L capability to modulate macrophages phenotype, murine RAW monocyte/macrophage-like cells were polarized toward an M1 phenotype, as confirmed by the increased expression of M1-related genes, *ITAGX* (Figure 6, panel A), and *NOS2* (Figure 6, panel B) at both 6 and 24 h. The response of NOS2 was higher at 24 h. At both time points, 500 nM was the most effective L dose in reducing *ITAGX* expression (fold regulation −1.4 vs. M1, 6h; fold regulation −1.5 vs. M1, 24 h) (Figure 6, panel A) and *NOS2* expression (fold regulation −1.5 vs. M1, 6 h; fold regulation −2.27 vs. M1, 24 h) (Figure 6, panel B). At times and doses tested in vitro, we can confirm the ability of L to directly modulate macrophage phenotype.

### 3.7. Effect of HF Diet and HF Combined with Linagliptin Diet (HFL) on Circulating Chemokines and Cytokines

**Figure 7:**
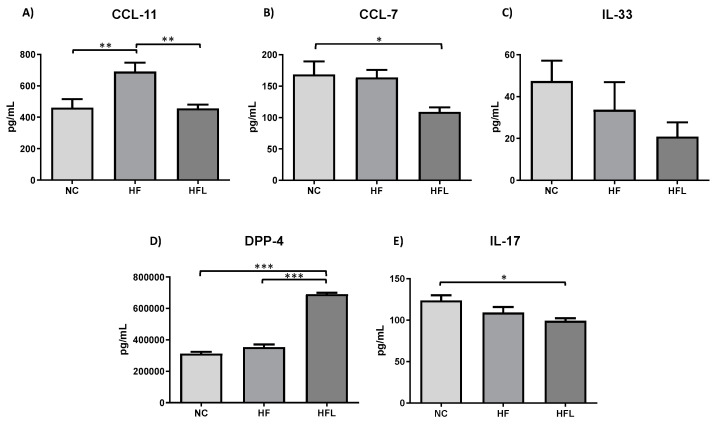
Effect of high-fat diet (HF) and high-fat diet combined with Linagliptin (HFL) on circulating chemokines and cytokines. (**A**–**E**) shows the effect of HF diet and HFL diet on plasma levels of different cytokines and chemokines. * *p* < 0.05, ** *p* < 0.01, and *** *p* < 0.001. CCL11, C-C Motif Chemokine Ligand 11; CCL7, chemokine CC motif ligand 7; IL-33, interleukin-33; DPP-4; IL-17A, interleukin-17A.

To investigate the role of L in regulating HF-diet-induced systemic inflammation, circulating levels of small molecules involved in inflammatory processes have been assessed by Luminex assay (Figure 7). L significantly inhibited the increase in CCL-11 induced by HF diet (*p* < 0.01 HF vs. NC; *p* < 0.01 HFL vs. HF, panel A) and exacerbated the lowering effect of HF diet on CCL-7 and IL-17A levels (*p* < 0.05 for all, panel B, E). A trend of decrease was observed for IL-33 (panel C) but at a limit of statistical significance. L also significantly increased the circulating levels of DPP-4 both respect to NC (*p* < 0.001) and HF (*p* < 0.001) (panel D). CCL-5, IL-10, IL-1β, and TNFα were below the test sensitivity. IFNγ levels were below 8 pg/mL in all groups; therefore, IFNγ hadno clinical relevance.

## 4. Discussion

The main results of this study can be stated as follows: the addition of L to the HF diet had no effect on body weight but decreased VAT inflammation by lowering endotoxemia, endotoxemia-induced NLRC4 inflammasome activation, inflammation severity, and fat cell hypertrophy. L can regulate inflammation by directly affecting pro-inflammatory macrophage phenotype, as demonstrated in vitro. Although the SAT response differed from VAT, a small reduction in inflammation was observed also in this fat depot.

Mice fed the HF diet for 15 W gained weight, had hypertrophic adipocytes in both VAT and SAT, and had more VAT-infiltrating macrophages and lymphocytes than mice fed the NC diet. These resident immune cells were organized into crown-like structures, which are commonly found around dying adipocytes [2,39]. The addition of L to the HF diet had no significant influence on weight gain or the number of infiltrating macrophages, as previously found [40,41]. However, by performing a detailed morphological analysis, we observed less-flattened nuclei in some samples of HFL group, a broader dimensional range but less variability of HFL adipocyte area and diameter than HF adipocytes, and a trend of a decrease in both cell area and diameter at a limit of statistical significance. All together, these data suggested a partial, though not yet statistically significant, reduction in adipocyte hypertrophy. Although the number of macrophages localized in VAT did not differ between the HF and HFL groups, the shift between pro- and anti-inflammatory macrophage phenotype can be an intriguing mechanism reducing tissue dysfunction.

Obesity and insulin resistance have been associated with increased NLRP3 expression in adipose tissue [42,43,44,45]. In this study, contrary to expectations, the HF diet had a smaller effect on the *NLRP3* inflammasome in VAT. We only appreciated the increased expression of *PANX1*, a channel-forming glycoprotein that can activate NLRP3 during apoptosis [33]. The strength of this study is the discovery that *NLRC4* was the most affected inflammasome in VAT. Indeed, the expression of multiple *NLRC4* components, including the sensor proteins *NAIP1* and *NAIP5*, as well as the adaptor protein *NLRC4*, rose in response to the HF diet. Among the several inflammasomes, NLRC4 activation has received relatively little attention in obesity. Given that the only known triggers of this inflammasome are components of the bacterial secretory system, such as LPS and flagellins, most of the NLRC4-related research has focused on its role in infections [46]. Given that HF diet and NLRC4 affect gut permeability and microbiota homeostasis, therefore contributing to endotoxemia and endotoxemia-related inflammation [47,48], the investigation of NLRC4 activation in obesity appears to be intriguing as well, but few data are available right now [49,50]. Kolb R. et al. proposed NLRC4 activation as a causative mechanism for obesity-related breast cancer development [49]. Herrero-Aguayo V. et al. discovered that the expression of several NLRC4 inflammasome components in peripheral blood mononuclear cells decreased 6 months after bariatric surgery [50]. By quantifying LPB, we confirmed that HF diet caused endotoxemia in mice. LBP is an acute phase protein mostly produced by the liver, which binds to LPS and stimulates an immune response via interactions with some macrophage receptors such as CD14 (cluster differentiation factor 14), TLR2 (toll-like receptor 2), and TLR4. LBP quantification is preferable over LPS quantification due to its longer half-life. Obesity, as previously demonstrated, can increase intestinal permeability, induce endotoxemia, and activate other inflammatory pathways that can lead to adipocyte dysfunctions [50,51]. For the first time, we found that L attenuated both HF diet-induced endotoxemia and NLRC4 activation in VAT. It is worth noting that the NLRC4 inflammasome was predominantly upregulated in VAT rather than SAT or other organs (kidney and liver—data not shown). In obesity, VAT is known to be infiltrated by more pro-inflammatory macrophages than SAT [52], and these cells express a greater number of LBP-interacting receptors. Bacteria-derived compounds may thus easily target resident VAT immune cells and induce the production of NLRC4 inflammasome components. The result is the local activation of an inflammatory pathway, which can exacerbate local VAT inflammation. L might reverse these effects by acting in the gut, regulating permeability and endotoxemia, as well as modifying local macrophage infiltration. L did not inhibit macrophage infiltration, but the enhanced expression of macrophage markers normally produced by M2-polarized macrophages suggested a direct modulatory effect on cell phenotype. In vitro investigations further confirmed that L may directly affect macrophage polarization, thus lowering the pro-inflammatory response, regardless of the presence of LBP [20].

The response to the HF diet was tissue-specific. This is supported by the finding that numerous inflammasome-related transcripts were differentially modulated in VAT and SAT. *IL-33*, *MAPK13*, *MAPK3*, and *NAIP1* were the only transcripts affected by HF diet in both tissues. However, with the exception of *MAPK3*, all transcripts in SAT were downregulated rather than increased, as in VAT. Looking at the genes that were differently regulated in SAT, we can see a balanced response in which both pro-inflammatory and anti-inflammatory pathways appear to be engaged at the same time. The sensor protein of the NLRC4 inflammasome, *NAIP1*, was found to be downregulated in SAT as compared to VAT. The decrease in *IL-12* expression in SAT may indicate a non-canonical macrophage activation pattern and compensatory anti-inflammatory response developed simultaneously, as further suggested by the decreased levels of other pro-inflammatory signals and mediators of the inflammatory response (*CXCL3*, *CD40L*, *CCL11*, *CCL-5*, *MEVF, NAIP1*, *PEA15G*, *and PTGS2*). Because we did not investigate any regulatory mechanism to explain the differential expression of multiple transcripts in the two tissues, we can only conclude that they are regulated by different signals. Currently, we do not have any molecular data to explain why the two tissues differently responded to L. In our opinion, this may be related to the difference in inflammatory conditions between VAT and SAT. VAT accumulation is mostly due to a hypertrophic reaction. However, hypertrophic visceral adipocytes have a lower plasticity than subcutaneous adipocytes; they experience apoptosis, leak lipids into the environment, and stimulate an immunological response. Given these assumptions, the absence of any effect of L on SAT could reflect a pharmacological activity limited to pathways that are engaged differentially in the two depots.

The effect of L on obesity-related inflammation was also studied with serum biomarkers. Several chemokines and cytokines, such as CCL-5, IL-10, IL-1β, and TNFα, were below the assay’s detection limit. These findings do not rule out the possibility of a low-grade inflammatory state generated by the HF diet, as seen in obesity, but more sensitive tests would be required to detect any potential difference between groups. L was shown to reduce CCL-11 and CCL-7 levels. These molecules have an important role in obesity-induced insulin resistance and obesity-related complications [53,54]. Lower serum levels of these molecules, together with the increased VAT expression of *SLC2A4*, suggested an overall improvement in insulin sensitivity, which could be due to effects of L on macrophage polarization and inflammation. In fact, inflammatory cytokines are well documented for their involvement in lowering *SLC2A4* levels and increasing insulin resistance [55]. Although obesity had no effect on IL-17 levels in our model, the addition of L lowered it, implying that L could also influence the development of T helper 17 cells. Like other compounds, sustained DPP-4 inhibition by L increased DPP-4 levels. Despite its putative pro-inflammatory action [56,57], soluble DPP-4 does not appear to interfere with the beneficial anti-inflammatory effects of L. Collectively, L seems to reduce the circulating levels of pro-inflammatory mediators, including those initiating signaling cascades, such as IL-33, that activate immune cells and stimulate the secretion of other pro-inflammatory cytokines, which can amplify the immune response.

## 5. Conclusions

NLRC4 is an emerging inflammasome that should be evaluated as a possible therapeutic target in the field of obesity. Endotoxemia and VAT inflammation may be just linked to increased *NLRC4* expression. L protected against endotoxemia possibly by affecting gut permeability and VAT responses. The decrease in the polarization of macrophages toward a pro-inflammatory phenotype and the reduction in adipocyte hypertrophy are two critical mechanisms involved in the response to L. Future research will address more in detail the mechanisms leading to the findings of this study.

## Figures and Tables

**Table 1:** Name, function, and fold regulation of genes affected by HF diet vs. NC diet in VAT. Gene transcripts are indicated according to their fold change, from least to most regulated.

Name	Function	Fold Regulation
*CASP8*	Pro-inflammatory thiol protease	+1.71
*MAPK8*	Protein kinase, which acts as a signaling downstream of NOD-like receptors	+1.72 *
*IRF1*	Transcriptional regulator of interferon and interferon-inducible genes	+1.85
*IL-33*	Alarmin: activator of both type 1 and 2 immune responses	+1.91
*TNF*	Proinflammatory cytokine	+2.22
*IL-18*	Pro-inflammatory cytokine	+2.26
*MAPK3*	Protein kinase, which acts as a signaling downstream of NOD-like receptors	+2.48 **
*PTGS2*	The inducible isoenzyme involved in prostaglandin synthesis	+2.64 *
*PYCARD*	Adapter in the assembly of various inflammasomes	+3.08
*PANX1*	Component of membrane channels involved in apoptosis and promoter of NLRP3 inflammasome [33]	+3.13
*CASP1*	Pro-inflammatory thiol protease	+3.18 *
*NLRC4*	Protein of NLRC4 inflammasome complex that activates CASP1	+5.89 *
*NAIP5*	Sensor protein of the NLRC4 inflammasome	+6.97
*NLRP9B*	Sensor protein of NLRP9B inflammasome that activates CASP1	+8.80
*NAIP1*	Sensor protein of the NLRC4 inflammasome	+9.04
*MAPK13*	Protein kinase, which acts as a signaling downstream of NOD-like receptors	+11.50 *
*NLRP6*	Sensor protein of NLRP6 inflammasome that activates CASP1 and CASP4	+11.64 *
*TNFSF4*	Involved in T-cell-dependent negative regulation of inflammasomes [34]	−1.81
*CCL7*	Chemokine for monocytes and eosinophils	−2.03

HF, high-fat diet; NC, normal chow; *CASP1,* caspase-1; *CASP4*, caspase-4; *CASP8*, caspase-8; *IL-18,* interleukin-18; *IRF1*, interferon regulatory factor 1; *MAPK13*, mitogen-activated protein kinase 13; *MAPK3*, mitogen-activated protein kinase 3; *NOD*, nucleotide-binding oligomerization domain; *MAPK8*, mitogen-activated protein kinase 8; *NAIP1*, nucleotide-binding domain and leucine-rich repeat containing protein (NLR) family apoptosis inhibitory protein 1; *NAIP5*, NLR family apoptosis inhibitory protein 5; *NLRC4*, NLR family CARD domain containing 4; *NLRP6*, NLR family pyrin domain containing 6; *NLRP9B*, NLR family pyrin domain containing 9; *PANX1*, pannexin 1; *PTGS2*, prostaglandin-endoperoxide synthase 2; *PYCARD*, PYD and CARD domain containing; *TNF*, tumor necrosis factor; *CCL7*, monocyte chemoattractant protein 3; *TNFSF4*, TNF superfamily member 4. * *p* < 0.05 and ** *p* < 0.01 vs. NC. GeneCrads was used as a source of information on the functions of genes (https://www.genecards.org/, (accessed on 12 october 2024)).

**Table 2:** Name, function and fold regulation of genes affected by HF combined with Linagliptin diet (HFL) vs. HF diet in VAT. Gene transcripts are indicated according to their fold change, from least to most regulated.

Gene Name	Gene Role	Fold Regulation
*NLRP6*	Sensor protein of NLRP6 inflammasome that activates CASP1 and CASP4	−1.70
*CASP1*	Pro-inflammatory thiol protease	−1.75
*MAPK13*	Protein kinase, which acts as a signaling downstream of NOD-like receptors	−1.94
*PYCARD*	Adapter in the assembly of various inflammasomes	−1.95
*NAIP5*	Sensor protein of the NLRC4 inflammasome	−2.29
*NAIP1*	Sensor protein of the NLRC4 inflammasome	−2.31
*NLRC4*	Protein of NLRC4 inflammasome complex that activates CASP1	−2.44
*NLRP9B*	Sensor protein of NLRP9 inflammasome that activates CASP1	−2.80
*CCL-7*	Chemokine for monocytes and eosinophils	+2.34

HF, high-fat diet; HFL, high-fat Linagliptin diet; *CASP1*, caspase-1; *MAPK13*, mitogen-activated protein kinase 3; *NAIP1*, nucleotide binding domain and leucine-rich repeat containing protein (NLR) family apoptosis inhibitory protein 1; *NAIP5*, NLR family apoptosis inhibitory protein 5; *NLRC4*, NLR family CARD domain containing 4; *NLRP6*, NLR family pyrin domain containing 6; *NLRP9B*, NLR family pyrin domain containing 9; *PYCARD*, PYD and CARD domain containing; *CCL7*, monocyte chemoattractant protein 3. GeneCrads was used as a source of information on the functions of genes (https://www.genecards.org/).

**Table 3:** Name, function, and fold regulation of genes affected by HF diet vs. NC diet in SAT. Gene transcripts are indicated according to their fold change, from least to most regulated.

Gene Name	Gene Role	Fold Regulation
*BIRC2*	Apoptosis inhibitor	−1.84
*IRF4*	Negative regulator of inflammation	−1.85
*PTGS2*	The inducible isoenzyme involved in prostaglandin synthesis	−2.10
*IFNB1*	Suppressor of immune cell infiltration into adipose tissue and cytokine production	−2.13
*MAPK13*	Protein kinase, which acts as a signaling downstream of NOD-like receptors	−2.23
*CXCL3*	Chemoattractant for neutrophils	−2.26 *
*NAIP1*	Sensor protein of the NLRC4 inflammasome	−2.28
*CCL-12*	Chemotactic factor for eosinophils	−2.30
*IL-33*	Alarmin: activator of both type 1 and 2 immune responses	−2.30
*CARD6*	Controversial reports in modulating NF-κB. Protects against steatosis and insulin resistance by suppressing apoptosis-signal-regulating kinase [35]	−2.35
*IL-12B*	Involved in the differentiation of Th1 and Th2 cells	−2.44
*CD40LG*	Inflammasome activator, expressed by T cells	−2.65
*IL-1B*	Proprotein, proteolytically processed to its active form by caspase 1	−2.78 *
*MEFV*	Involved in the degradation of several inflammasome components [36] It can also act as an innate immune sensor that triggers PYCARD/ASC specks formation, caspase-1 activation, and IL1B and IL18 production [37]	−3.25 *
*CCL-5*	Chemotactic factor for blood monocytes, memory T helper cells, and eosinophils	−3.44
*TNFSF14*	Involved T-cell-dependent negative regulation of inflammasomes	−3.74
*CIITA*	Positive regulator of MHCII, which can promotes immune activation of Th1 cells [38]	−4.36
*NFKBIB*	Inhibits NF-kappa-B by complexing with and trapping it in the cytoplasm	−5.02
*IL-12A*	Involved in the differentiation of Th1 and Th2 cells	−6.49
*TNFSF11*	Involved T-cell-dependent negative regulation of inflammasomes	−11.95 **
*NLRP1A*	Sensor component of the NLRP1 inflammasome	+1.99 *
*P2RX7*	The activation of this ATP receptor induces NLRP3 inflammasome	+2.19 *
*PEA15A*	Negative regulator of apoptosis	+2.46 **
*CTSB*	Increases autophagy in adipocytes and enhances inflammation and macrophage infiltration	+2.58 **
*MAPK3*	Protein kinase, which acts as a signaling downstream of NOD-like receptors	+2.37 **

HF, high-fat diet; NC, normal chow; *BIRC2*, Baculoviral IAP Repeat Containing 2; *CARD6*, Caspase Recruitment Domain Family Member 6; *NOD*, Nucleotide Binding Oligomerization Domain Containing; *CCL12*, C-C Motif Chemokine Ligand 12; *CCL-5*, C-C Motif Chemokine Ligand 5; *CD40LG*, CD40 Ligand; *CIITA*, Class II Major Histocompatibility Complex Transactivator; *CTSB*, Cathepsin B; *CXCL3*, C-X-C Motif Chemokine Ligand 3; *IFNB1*, Interferon Beta 1; *IL-12A*, interleukin-12A; *IL-12B*, interleukin-12B; *IL-1B*, interleukin-1B; *IL-33*, Interleukin 33; *IRF4*, Interferon Regulatory Factor 4; *MAPK13*, Mitogen-Activated Protein Kinase 13; *MAPK3*, Mitogen-Activated Protein Kinase 3; *MEFV*, Mediterranean fever; *NAIP1*, nucleotide binding domain and leucine-rich repeat containing protein—NLR—family apoptosis inhibitory protein 1; *NFKBIB*, NFKB inhibitor beta; *NLRP1A*, NLR family pyrin domain containing 1; *P2RX7*, Purinergic Receptor P2X7; *PEA15A*, Proliferation and Apoptosis Adaptor Protein 15; *PTGS2*, Prostaglandin-Endoperoxide Synthase 2—COX2; *TNFSF11*, tumor necrosis factor (ligand) superfamily, member 11; *TNFSF14*, tumor necrosis factor (ligand) superfamily, member 14. * *p* < 0.05 and ** *p* < 0.01 vs. NC. GeneCrads was used as a source of information on the functions of genes (https://www.genecards.org/).

## Data Availability

The raw data supporting the conclusions of this article will be made available by the authors on request.

## Supplementary Materials

The following supporting information can be downloaded at https://www.mdpi.com/article/10.3390/biom15030333/s1. Supplementary File S1: List of all transcripts included in the array panel; Supplementary File S2: Histological assessment of sections of adipocytes of VAT and SAT of mice fed NC, HF, and HFL diets; Supplementary File S3: Images of VAT sections of mice fed NC diet with the manual count of adipocytes on one 400× magnification field; Supplementary File S4: Images of VAT sections of mice fed HF diet with the manual count of adipocytes on one 400× magnification field; Supplementary File S5: Images of VAT sections of mice fed HFL diet with the manual count of adipocytes on one 400× magnification field; Supplementary File S6: Images of VAT sections of mice fed NC diet with the manual count of adipocytes on one 400× magnification field; Supplementary File S7: Images of SAT sections of mice fed HF diet with the manual count of adipocytes on one 400× magnification field; Supplementary File S8: Images of SAT sections of mice fed HFL diet with the manual count of adipocytes on one 400× magnification field.
