# Supplementary material for: Inhibition of DPP-4 Attenuates Endotoxemia-Induced NLRC4 Inflammasome and Inflammation in Visceral Adipose Tissue of Mice Fed a High-Fat Diet
Source: Biomolecules. 2025 Feb 25;15(3):333. doi: 10.3390/biom15030333 (PMC11940500; doi:10.3390/biom15030333)
Supplement: Supplementary file 1 [file biomolecules-15-00333-s001.zip › Supplementary file 1.pdf]

S1. Supplementary file 1

INFLAMMASOMES

AIM2: AIM2, CASP1 (ICE), PYCARD (TMS1, ASC)
IPAF: CASP1 (ICE), NAIP1 (BIRC1), NAIP5, NLRC4 (IPAF), PYCARD (TMS1, ASC)
NLRP1: CASP1 (ICE), NLRP1A
NLRP3: CASP1 (ICE), NLRP3, PYCARD (TMS1, ASC)
NEGATIVE REGULATION OF INFLAMMASOMES: BCL2, BCL2L1 (BCLXL), CD40LG, CTSB, HSP90AA1, HSP90B1, MEFV, PSTPIP1, SUGT1, TNF, TNFSF11 (RANKL), TNFSF14, TNFSF4 (OX40L)
SIGNALING DOWNSTREAM OF INFLAMMASOMES: IFNG, IL12A, IL12B, IL18, IL1B, IL33, IRAK1, IRF1, MOK, MYD88, P2RX7, PANX1, PTGS2 (COX2), RIPK2, TXNIP

NOD-LIKE RECEPTORS

NOD-LIKE RECEPTORS: CIITA, NAIP1 (BIRC1), NAIP5, NLRC4 (IPAF), NLRC5, NLRP12 (NALP12, PYPAF7), NLRP1A, NLRP3, NLRP4B, NLRP4E, NLRP5, NLRP6, NLRP9B, NLRX1, NOD1, NOD2
SIGNALING DOWNSTREAM OF NOD-LIKE RECEPTORS: BIRC2 (CIAP1, CIAP2), BIRC3 (CIAP1, CIAP2), CARD6, CASP8 (FLICE), CCL12 (MCP-5, SCYA12), CCL5 (RANTES), CCL7 (MCP3), CFLAR (CASPER), CHUK (IKBKA), CXCL1 (GRO1), CXCL3, FADD, IFNB1, IKBKB (IKKBETA), IKBKG, IL6, IRF1, IRF3, IRF4, MAP3K7 (TAK1), MAPK1 (ERK2), MAPK11 (P38BETA), MAPK12 (P38GAMMA), MAPK13 (SAPK4, SERK4), MAPK3 (ERK1), MAPK8 (JNK1), MAPK9 (JNK2), NFKB1, NFKBIA (IKBA, MAD3), NFKBIB (TRIP9), PEA15A, RELA, RIPK2, SUGT1, TAB1, TAB2, TIRAP, TNF, TRAF6, XIAP

PRO-INFLAMMATORY CASPASES

CASP1 (ICE), CASP12
