# Supplementary material for: Inhibition of DPP-4 Attenuates Endotoxemia-Induced NLRC4 Inflammasome and Inflammation in Visceral Adipose Tissue of Mice Fed a High-Fat Diet
Source: Biomolecules. 2025 Feb 25;15(3):333. doi: 10.3390/biom15030333 (PMC11940500; doi:10.3390/biom15030333)
Supplement: Supplementary file 1 [file biomolecules-15-00333-s001.zip › Supplementary file 2.pdf]

Supplementary file 2. Histological assessment of sections of adipocytes of VAT and SAT of mice fed NC, HF and HFL diets.

In VAT, NC adipocytes were of normal size, with visible plump nuclei. Perivascular inflammation was observed only in 1 case (**Supplementary file 3, #7**) with a severity score of 1 for macrophages and for small mature lymphocytes. No granulomas were observed. HF adipocytes were approximately 2 to 4 times larger than NC adipocytes, with mostly flat and hyperchromatic nuclei compressed at the periphery. Inflammation was observed in all mice and consisted of reactive foamy macrophages and small mature lymphocytes scoring 1 to 2 in severity, with a septal to perilobar arrangement (**Supplementary file 4**) and occasionally organized in granulomas (**Supplementary file 4, #2 and #5**). HFL adipocytes were like HF cells, although HFL adipocytes had often some plump nuclei and a variable, but generally mild inflammatory response in all mice. Inflammation in HFL diet group consisted of accumulation of septal to lobular reactive foamy macrophages with or without small mature lymphocytes scoring 1-3 in severity (**Supplementary file 6**) and occasionally organized in granulomas (2/8 mice) (**Supplementary file 5, #3 and #8**) that developed around degenerated fat material. Qualitative morphological findings were supported by the manual counts of the same adipocytes (**Supplementary file 2**).

Results of the morphological assessment of SAT samples paralleled results of VAT samples. Briefly, NC adipocytes were of normal size, with visible plump to flat nuclei and septal lymphocytic aggregates with a severity score of 1 in 2/8 mice (**Supplementary figure 6, #7 and #8**). No granulomas were observed. HF adipocytes were 2 to 4 times larger than NC cells, with mostly flat and hyperchromatic nuclei compressed at the periphery. Inflammation was observed in 3/5 mice consisting of septal accumulation of reactive foamy macrophages (2/5) (**Supplementary figure 7, #1 and #3**) or small mature lymphocytes (1/5) (**Supplementary figure 7, #5**) with a severity score of 1 to 2. The size HFL adipocytes was like HF adipocytes. Inflammation was observed in 6/8 mice and consisted of accumulation of septal reactive foamy macrophages and small mature lymphocytes, with a severity score of 1-2 in 6/8 mice (**Supplementary figure 8, #1, #3, #5, #6, #7, #8**). Granulomas with septal to lobular arrangement were observed only in one mouse (**Supplementary figure 8, #8**). Qualitative morphological findings were supported by the manual counts of the same adipocytes.

**GROUP 1. VAT NC**						
**SAMPLE**	**ADIPOCYTE MORPHOLOGY**	**ADIPOCYTE COUNTS**	**INFLAMMATORY CELL SCORE**			
			**Macrophages**	**Neutrophils**	**Lymphocytes**	**Plasmacells**
1	Normal size; plump nuclei	37	0	0	0	0
2	Normal size; plump nuclei	27	0	0	0	0
3	Normal size; plump nuclei	35	0	0	0	0
4	Normal size; plump nuclei	31	0	0	0	0
5	Normal size; plump nuclei	37	0	0	0	0
6	Larger size compared to other mice of the group; plump nuclei	23	0	0	0	0

7	Normal size, plump nuclei	34	1 Perivascular	0	1 Perivascular	0
8	Larger size compared to other mice of the group; plump nuclei	25	0	0	0	0
**GROUP 2. VAT HF**						
**SAMPLE**	**ADIPOCYTE MORPHOLOGY**	**ADIPOCYTE COUNTS**	**INFLAMMATORY CELL SCORE**			
			**Macrophages**	**Neutrophils**	**Lymphocytes**	**Plasmacells**
1	Very large; nuclei are flat, hyperchromatic, compressed at the periphery	8	1 Septal and Lobular; Granulomas	0	1 Septal	0
2	Very large; nuclei are flat, hyperchromatic, compressed at the periphery	12	1 Septal	0	1 Septal	0
3	Very large; nuclei are flat, hyperchromatic, compressed at the periphery	10	1 Septal	0	1 Septal	0
4	Very large; nuclei are flat, hyperchromatic, compressed at the periphery	15	1 Septal and Perilobular	0	0	0
5	Very large; nuclei are flat, hyperchromatic, compressed at the periphery	12	2 Septal and Lobular; Granulomas	0	2 Septal and Perilobular	0
**GROUP 3. VAT HFL**						
**SAMPLE**	**ADIPOCYTE MORPHOLOGY**	**ADIPOCYTE COUNTS**	**INFLAMMATORY CELL SCORE**			
			**Macrophages**	**Neutrophils**	**Lymphocytes**	**Plasmacells**
1	Very large; nuclei are flat, hyperchromatic, and compressed at the periphery; some plump nuclei	14	1 Septal	0	2 Septal	0
2	Very large; nuclei are flat, hyperchromatic, and compressed at the periphery; some plump nuclei	6	0	0	1 Septal	0
3	Very large; nuclei are flat, hyperchromatic, and compressed at the periphery; some plump nuclei	10	3 Septal and perilobular; Granulomas with central	1 Septal	2	0

			degenerated fat			
4	Very large; nuclei are flat, hyperchromatic, and compressed at the periphery; some plump nuclei	12	0	0	2	0
5	Very large; nuclei are flat, hyperchromatic, and compressed at the periphery; some plump nuclei	9	1 Septal	0	1 Septal	0
6	Very large; nuclei are flat, hyperchromatic, and compressed at the periphery; some plump nuclei	10	1 Septal	0	1 Septal	0
7	Adipocytes are larger than control, but with the smallest adipocytes of group; nuclei are flat hyperchromatic and compressed at the periphery; some plump nuclei	15	1 Septal	0	1 Septal	0
8	Very large; nuclei are flat, hyperchromatic, and compressed at the periphery; some plump nuclei	10	2 Septal and perilobular to lobular; Granulomas	0	1 Septal	0

GROUP 4. SAT NC						
**SAMPLE**	**ADIPOCYTE MORPHOLOGY**	**ADIPOCYTE COUNTS**	**INFLAMMATORY CELL SCORE**			
			**Macrophages**	**Neutrophils**	**Lymphocytes**	**Plasmacells**
1	Normal but variable size; nuclei are flat and plump in similar proportions	38	0	0	0	0
2	Normal but variable size; nuclei are flat and plump in similar proportions	41	0	0	0	0
3	Normal but variable size; nuclei are flat and plump in similar proportions	33	0	0	0	0
4	Normal but variable size; nuclei are flat, hyperchromatic, and	16	0	0	0	0

	compressed at the periphery; some plump nuclei					
5	Normal but variable size; nuclei are flat, hyperchromatic, and compressed at the periphery; some plump nuclei	15	0	0	0	0
6	Normal but variable size; nuclei are flat, hyperchromatic, and compressed at the periphery; some plump nuclei	26	0	0	0	0
7	Normal but variable size; nuclei are flat and plump in similar proportions	36	0	0	1<br>Septal	0
8	Normal but variable size; nuclei are flat, hyperchromatic, and compressed at the periphery; some plump nuclei	29	0	0	1<br>Septal	0

GROUP 5. SUBCUTANEOUS FAT HIGH FAT DIET

SAMPLE	ADIPOCYTE MORPHOLOGY	ADIPOCYTE COUNTS	INFLAMMATORY CELL SCORE			
			Macrophages	Neutrophils	Lymphocytes	Plasmacells
1	Large but variable size. Nuclei are flat, hyperchromatic, and compressed at the periphery.	12	1<br>Septal	0	0	0
2	Large but variable size. Nuclei are flat, hyperchromatic, and compressed at the periphery.	8	0	0	0	0
3	Large but variable size. Nuclei are flat, hyperchromatic, and compressed at the periphery.	19	1<br>Septal	0	0	0
4	Large but variable size. Nuclei are flat, hyperchromatic, and compressed at the periphery.	10	0	0	0	0

5	Large but variable size. Nuclei are flat, hyperchromatic, and compressed at the periphery.	11	0	0	2 Septal	0

GROUP 6. SUBCUTANEOUS FAT HIGH FAT DIET WITH LINAGLIPTIN ADMINISTRATION

SAMPLE	ADIPOCYTE MORPHOLOGY	ADIPOCYTE COUNTS	INFLAMMATORY CELL SCORE			
			Macrophages	Neutrophils	Lymphocytes	Plasmacells
1	Large but variable size. Nuclei are flat, hyperchromatic, and compressed at the periphery.	18	1 Septal	0	1 Septal	0
2	Large but variable size. Nuclei are flat, hyperchromatic, and compressed at the periphery. Some plump nuclei	8	0	0	0	0
3	Adipocytes are large. Nuclei are mostly flat, compressed at the periphery hyperchromatic.	12	1 Septal	0	0	0
4	Large size. Nuclei are flat, hyperchromatic, and compressed at the periphery.	12	0	0	0	0
5	Large size. Nuclei are flat, hyperchromatic, and compressed at the periphery.	10	1 Septal	0	0	0
6	Large size. Nuclei are flat, hyperchromatic, and compressed at the periphery.	9	1 Septal	0	0	0
7	Large size. Nuclei are flat, hyperchromatic, and compressed at the periphery.	10	1 Septal	0	0	0
8	Large size. Nuclei are flat, hyperchromatic, and compressed at the periphery.	10	2 Septal to lobular; Granulomas	0	Septal	0
