# Supplementary material for: Inhibition of DPP-4 Attenuates Endotoxemia-Induced NLRC4 Inflammasome and Inflammation in Visceral Adipose Tissue of Mice Fed a High-Fat Diet
Source: Biomolecules. 2025 Feb 25;15(3):333. doi: 10.3390/biom15030333 (PMC11940500; doi:10.3390/biom15030333)
Supplement: Supplementary file 1 [file biomolecules-15-00333-s001.zip › Supplementary file 3 VAT_NC.pptx]

## Slide 1
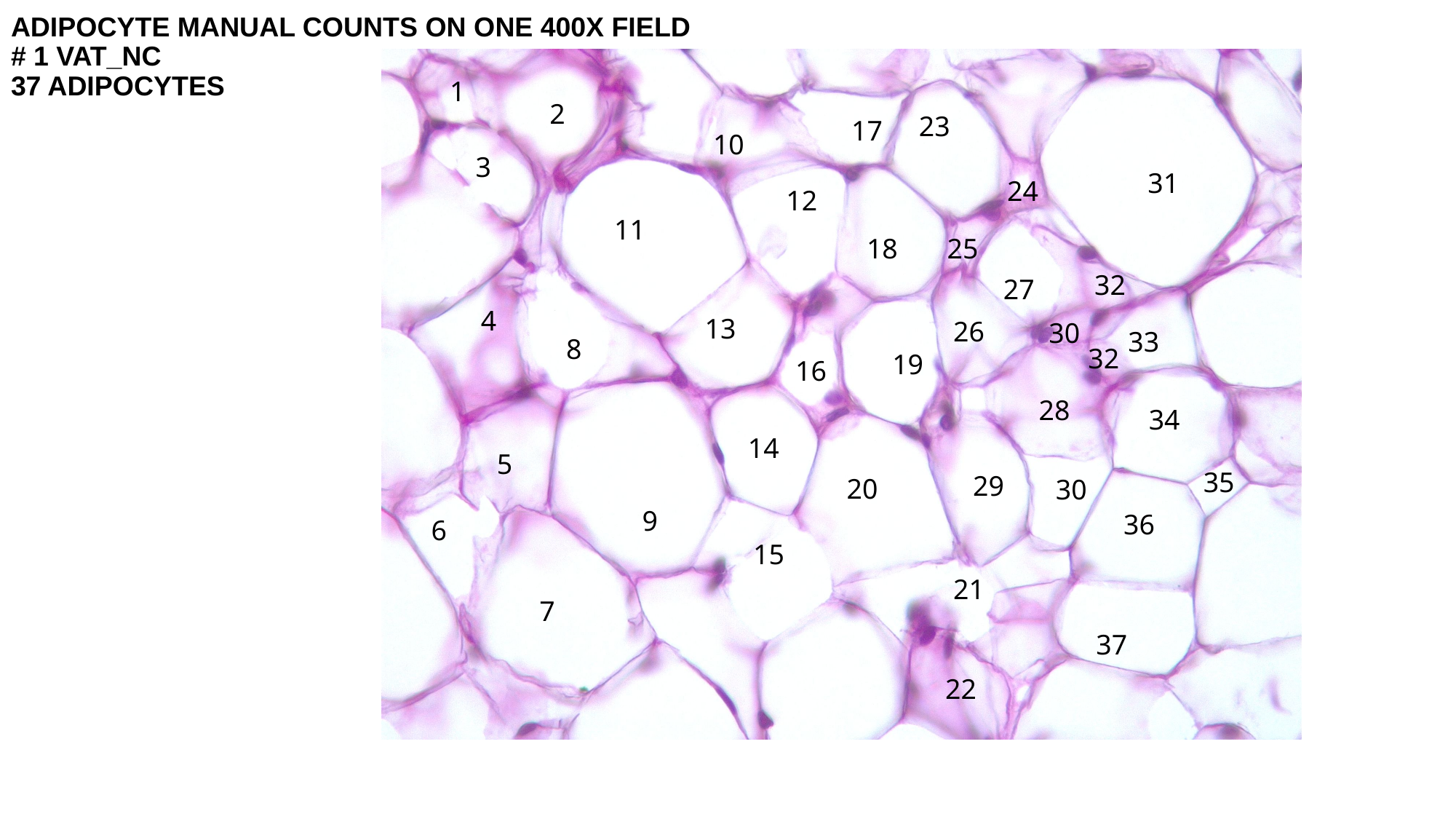

# ADIPOCYTE MANUAL COUNTS ON ONE 400X FIELD # 1 VAT_NC 37 ADIPOCYTES
1
2
23
17
10
3
31
24
12
11
18
25
32
27
4
13
26
30
33
8
32
19
16
28
34
14
5
35
29
20
30
9
36
6
15
21
7
37
22

## Slide 2
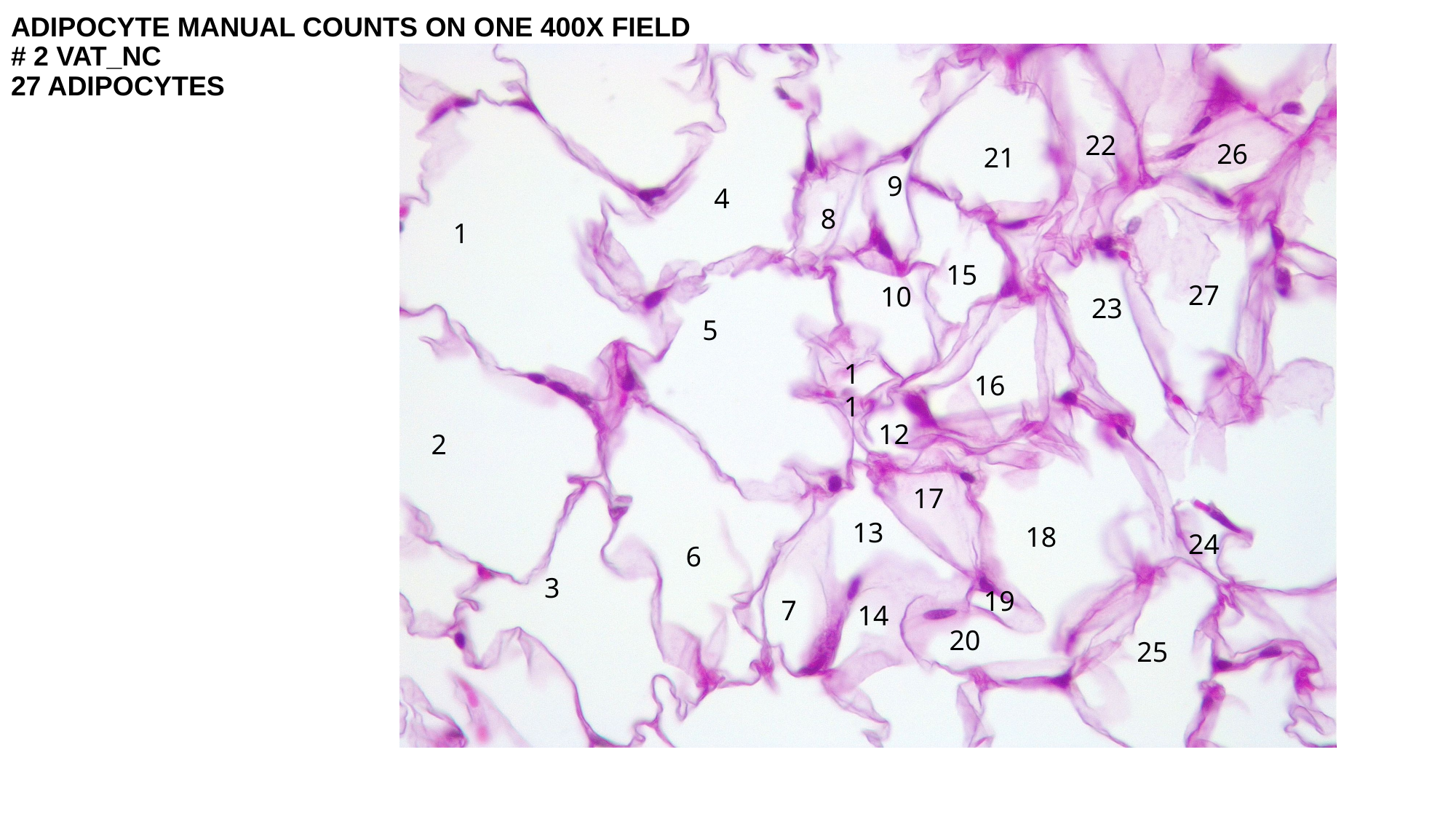

# ADIPOCYTE MANUAL COUNTS ON ONE 400X FIELD # 2 VAT_NC 27 ADIPOCYTES
22
26
21
9
8
1
15
27
10
5
11
12
2
13
18
24
6
3
19
7
14
4
23
16
17
20
25

## Slide 3
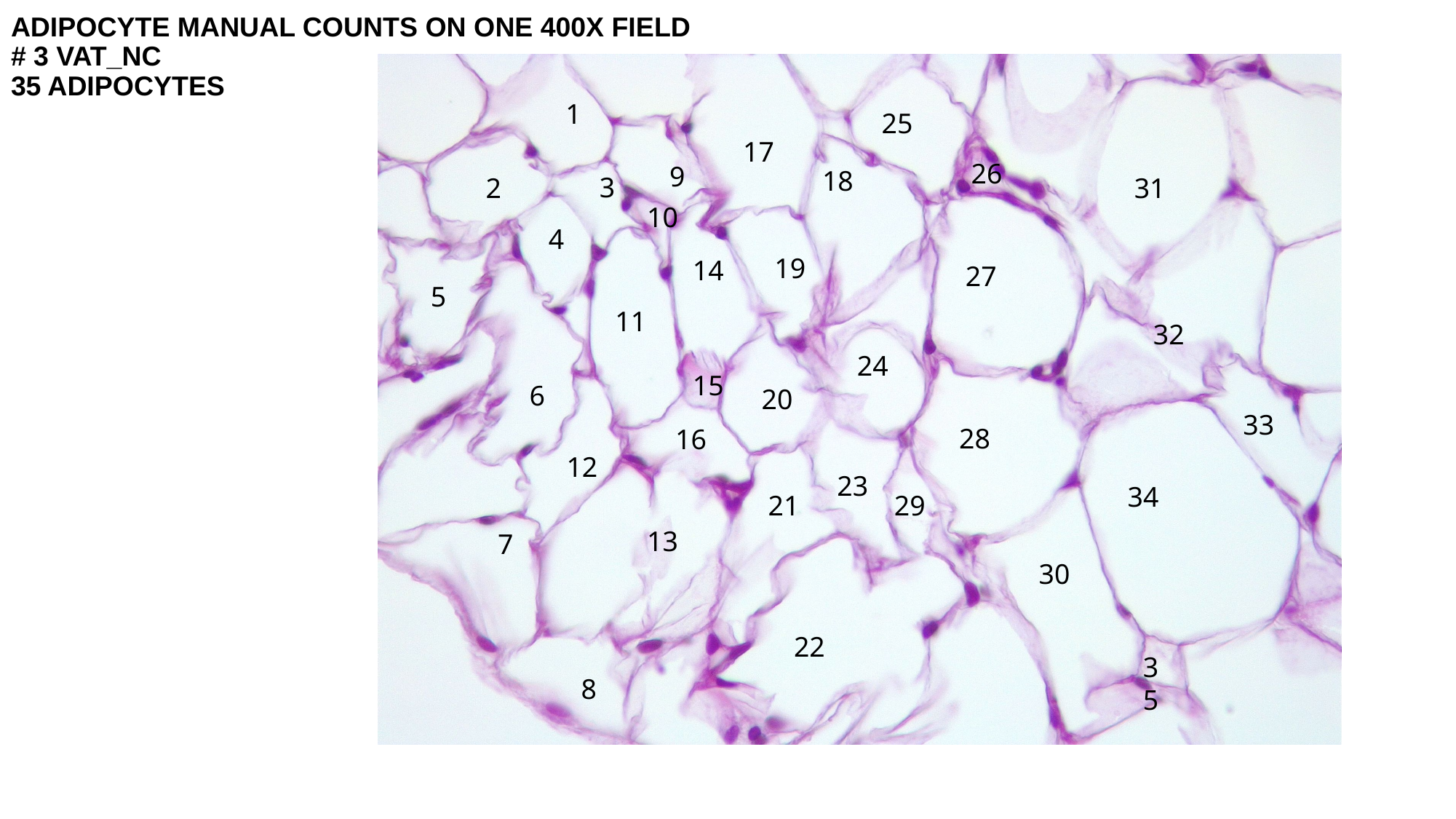

# ADIPOCYTE MANUAL COUNTS ON ONE 400X FIELD # 3 VAT_NC 35 ADIPOCYTES
1
25
17
26
9
18
3
31
2
10
4
19
14
27
5
11
32
24
15
6
20
33
28
16
12
23
34
21
29
13
7
30
22
35
8

## Slide 4
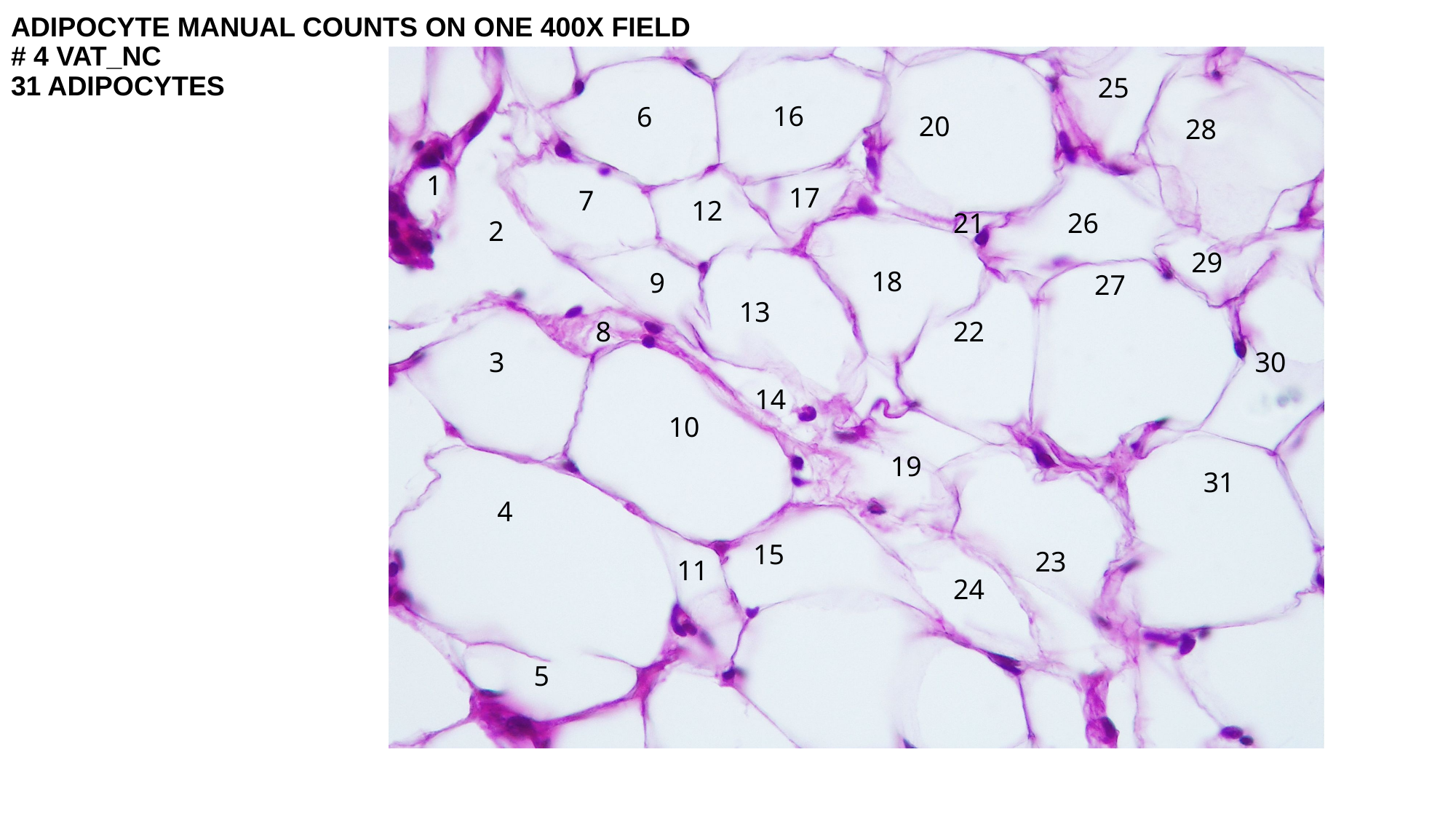

# ADIPOCYTE MANUAL COUNTS ON ONE 400X FIELD # 4 VAT_NC 31 ADIPOCYTES
25
16
6
20
28
1
17
7
12
21
26
2
29
18
9
27
13
8
22
3
30
14
10
19
31
4
15
23
11
24
5

## Slide 5
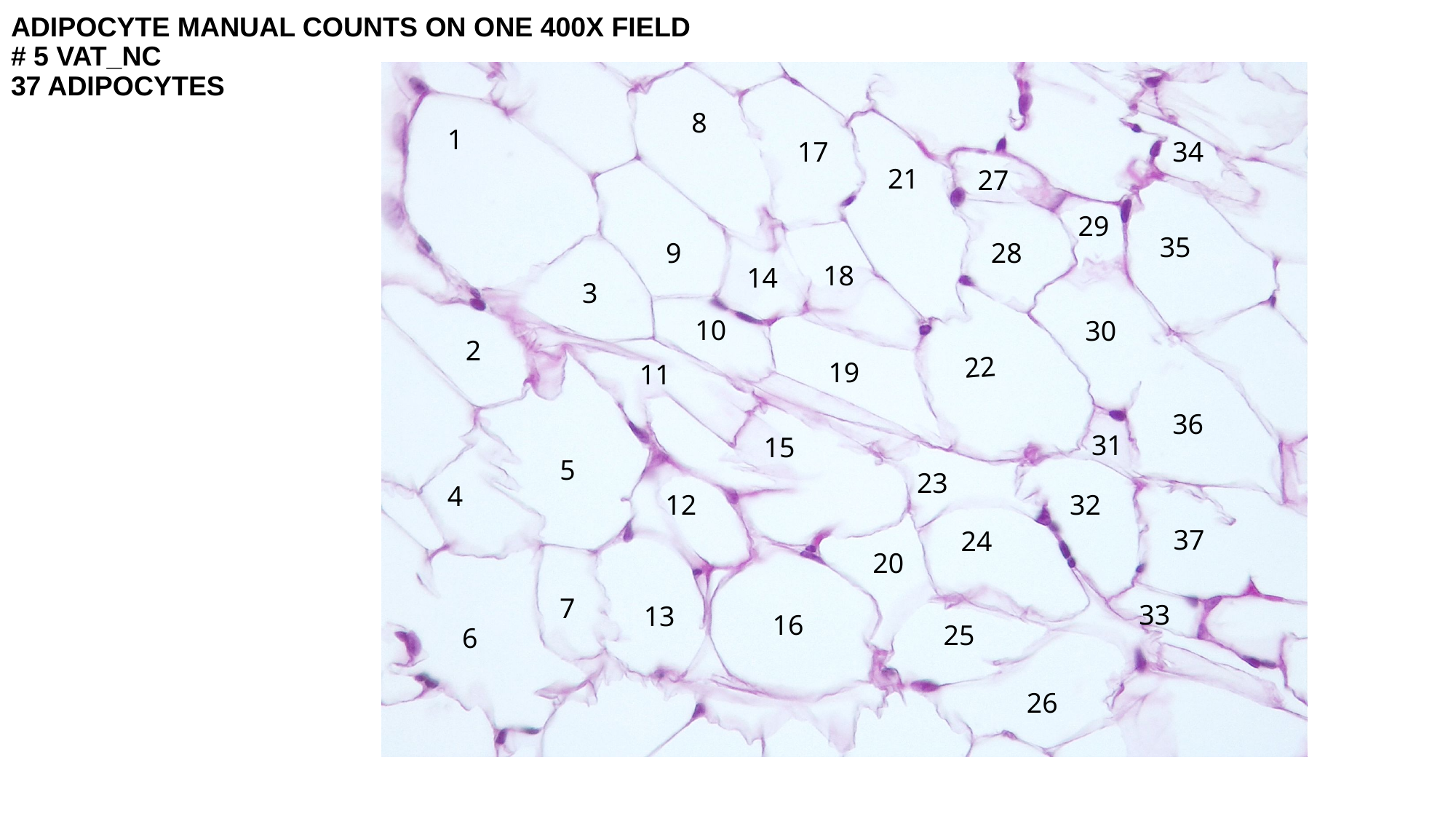

# ADIPOCYTE MANUAL COUNTS ON ONE 400X FIELD # 5 VAT_NC 37 ADIPOCYTES
8
1
17
34
21
27
29
35
28
9
18
14
3
10
30
2
22
19
11
36
31
15
5
23
4
12
32
37
24
20
7
33
13
16
25
6
26

## Slide 6
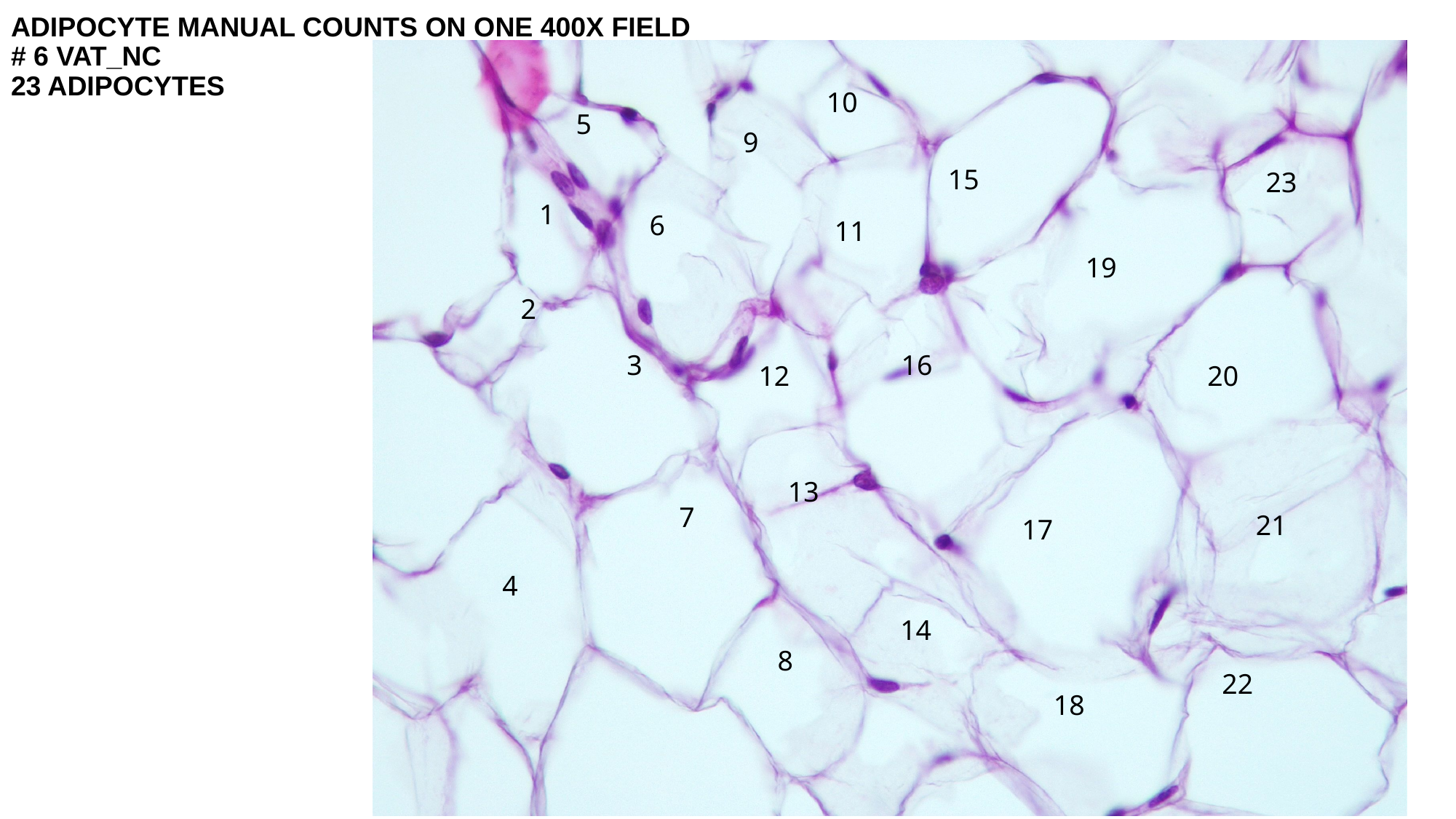

# ADIPOCYTE MANUAL COUNTS ON ONE 400X FIELD # 6 VAT_NC 23 ADIPOCYTES
10
5
9
15
1
6
11
19
2
3
16
12
13
7
17
4
14
8
18
23
20
21
22

## Slide 7
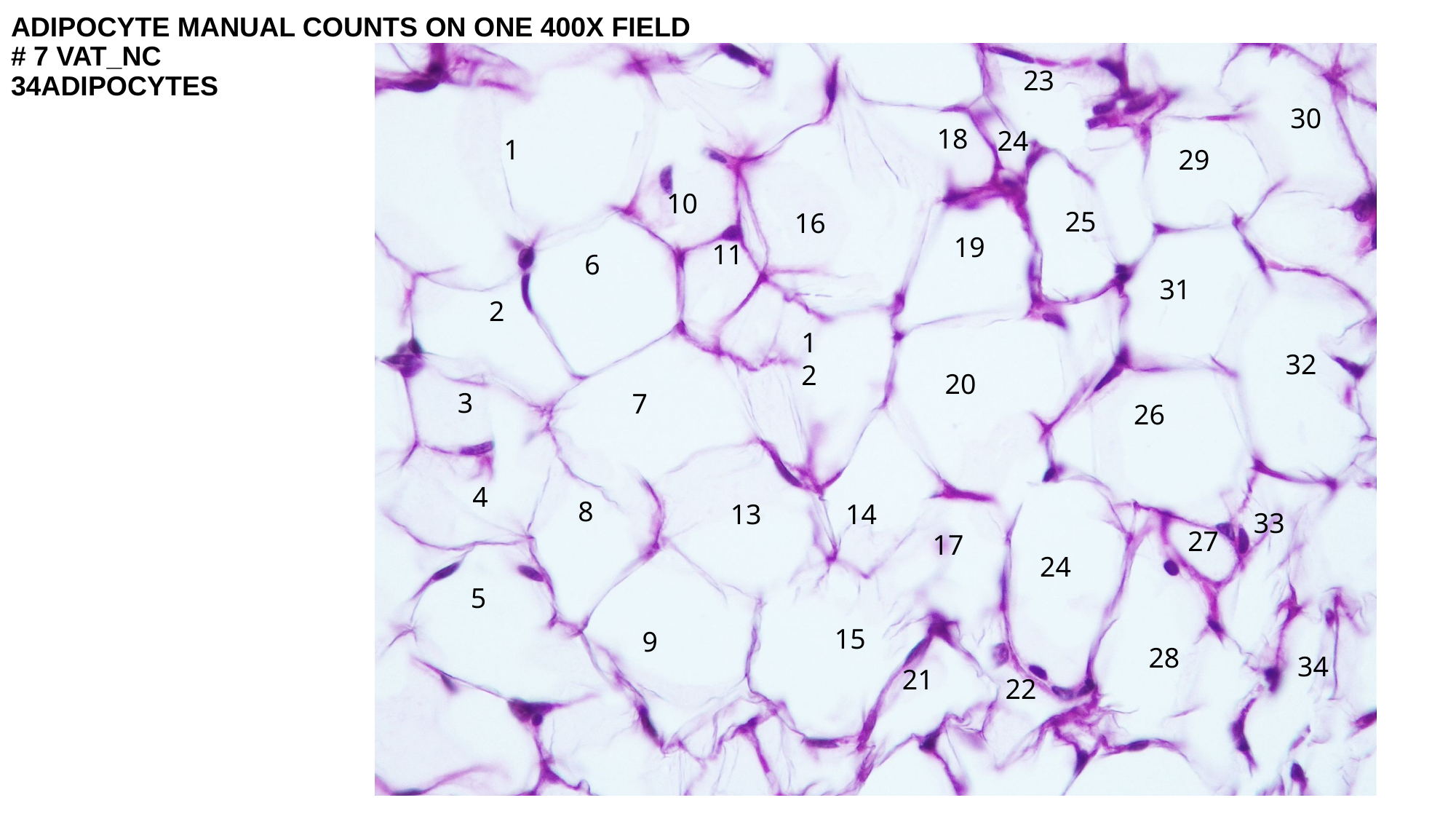

# ADIPOCYTE MANUAL COUNTS ON ONE 400X FIELD # 7 VAT_NC 34ADIPOCYTES
23
30
18
24
1
29
10
25
16
19
11
6
31
2
12
32
20
3
7
26
4
8
13
14
33
27
17
24
5
15
9
28
34
21
22

## Slide 8
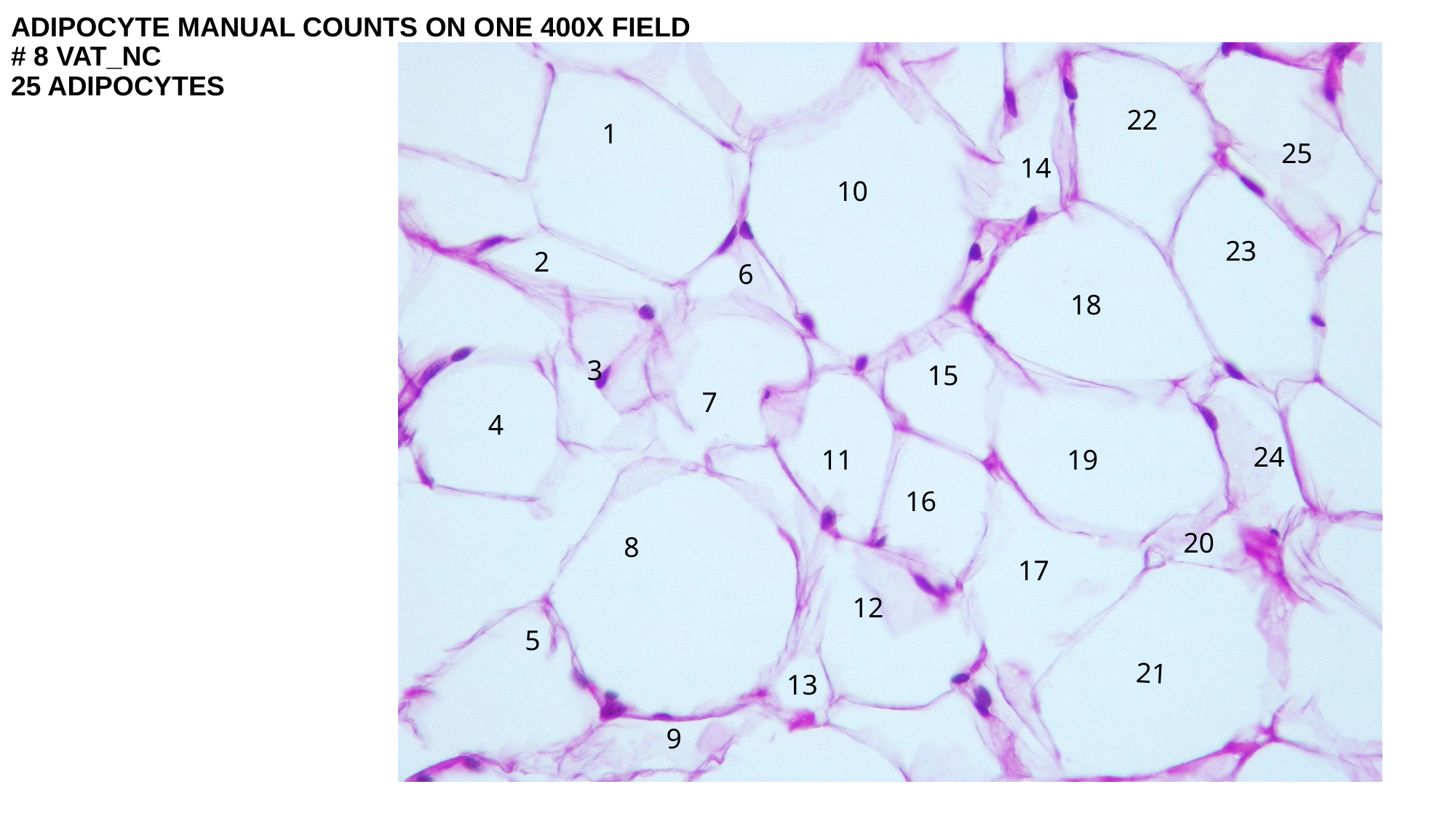

# ADIPOCYTE MANUAL COUNTS ON ONE 400X FIELD # 8 VAT_NC 25 ADIPOCYTES
22
1
25
14
10
23
2
6
18
3
15
7
4
24
11
19
16
20
8
17
12
5
21
13
9
