# Supplementary material for: Inhibition of DPP-4 Attenuates Endotoxemia-Induced NLRC4 Inflammasome and Inflammation in Visceral Adipose Tissue of Mice Fed a High-Fat Diet
Source: Biomolecules. 2025 Feb 25;15(3):333. doi: 10.3390/biom15030333 (PMC11940500; doi:10.3390/biom15030333)
Supplement: Supplementary file 1 [file biomolecules-15-00333-s001.zip › Supplementary file 4 VAT_HF.pptx]

## Slide 1
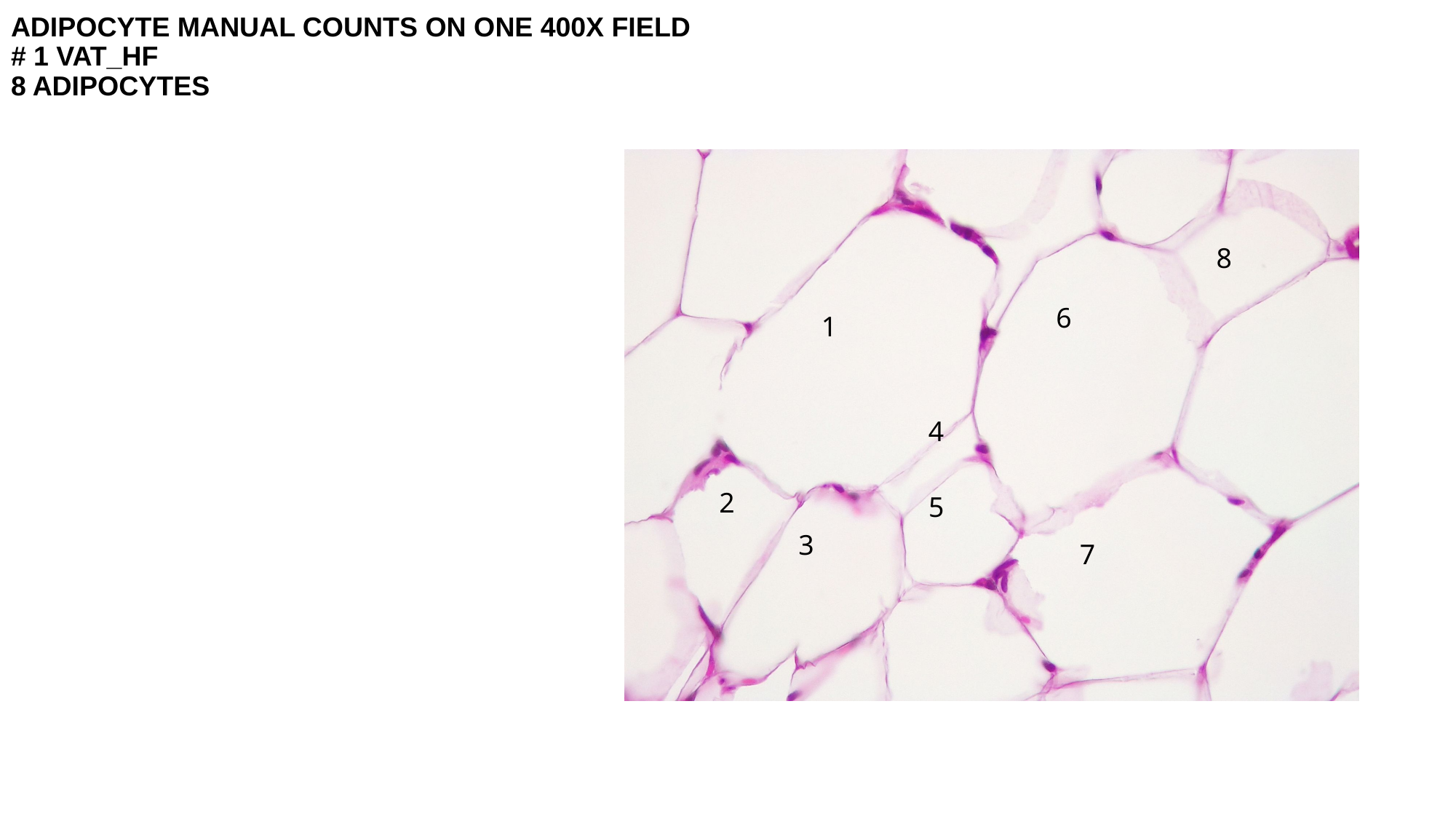

# ADIPOCYTE MANUAL COUNTS ON ONE 400X FIELD # 1 VAT_HF 8 ADIPOCYTES
8
6
1
4
2
5
3
7

## Slide 2
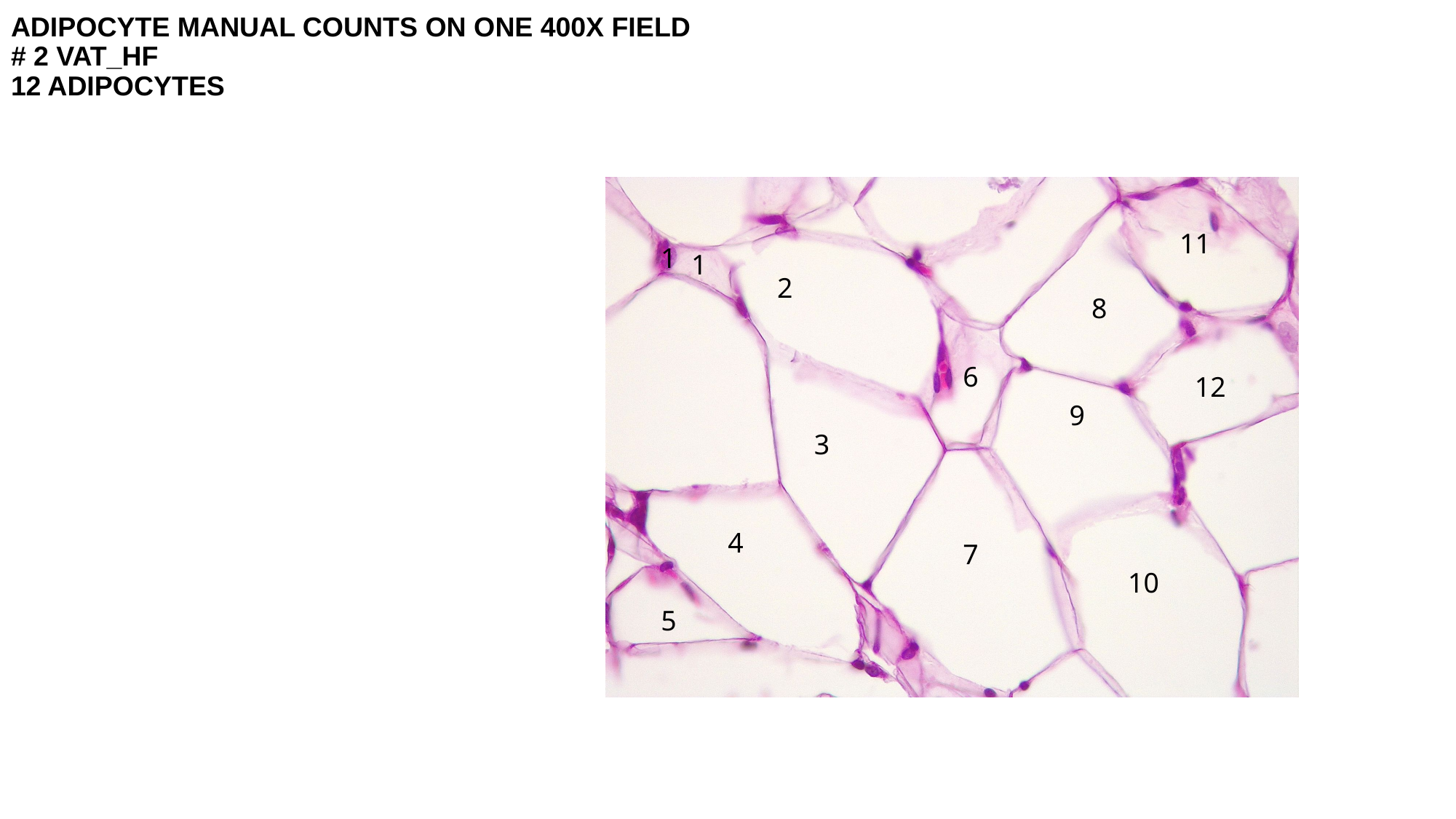

# ADIPOCYTE MANUAL COUNTS ON ONE 400X FIELD # 2 VAT_HF 12 ADIPOCYTES
11
1
1
2
8
6
12
9
3
4
7
10
5

## Slide 3
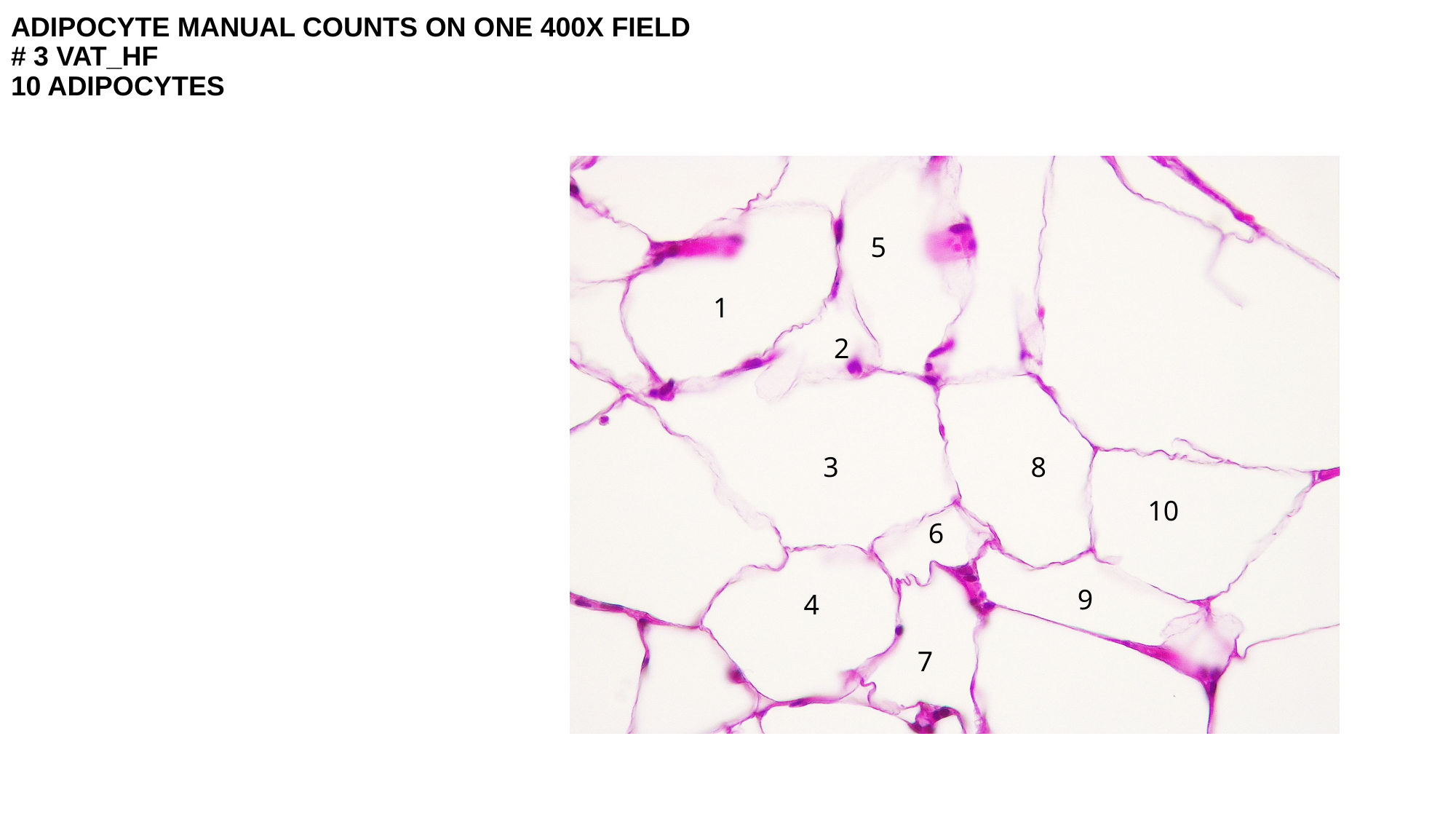

# ADIPOCYTE MANUAL COUNTS ON ONE 400X FIELD # 3 VAT_HF 10 ADIPOCYTES
5
1
2
8
3
10
6
9
4
7

## Slide 4
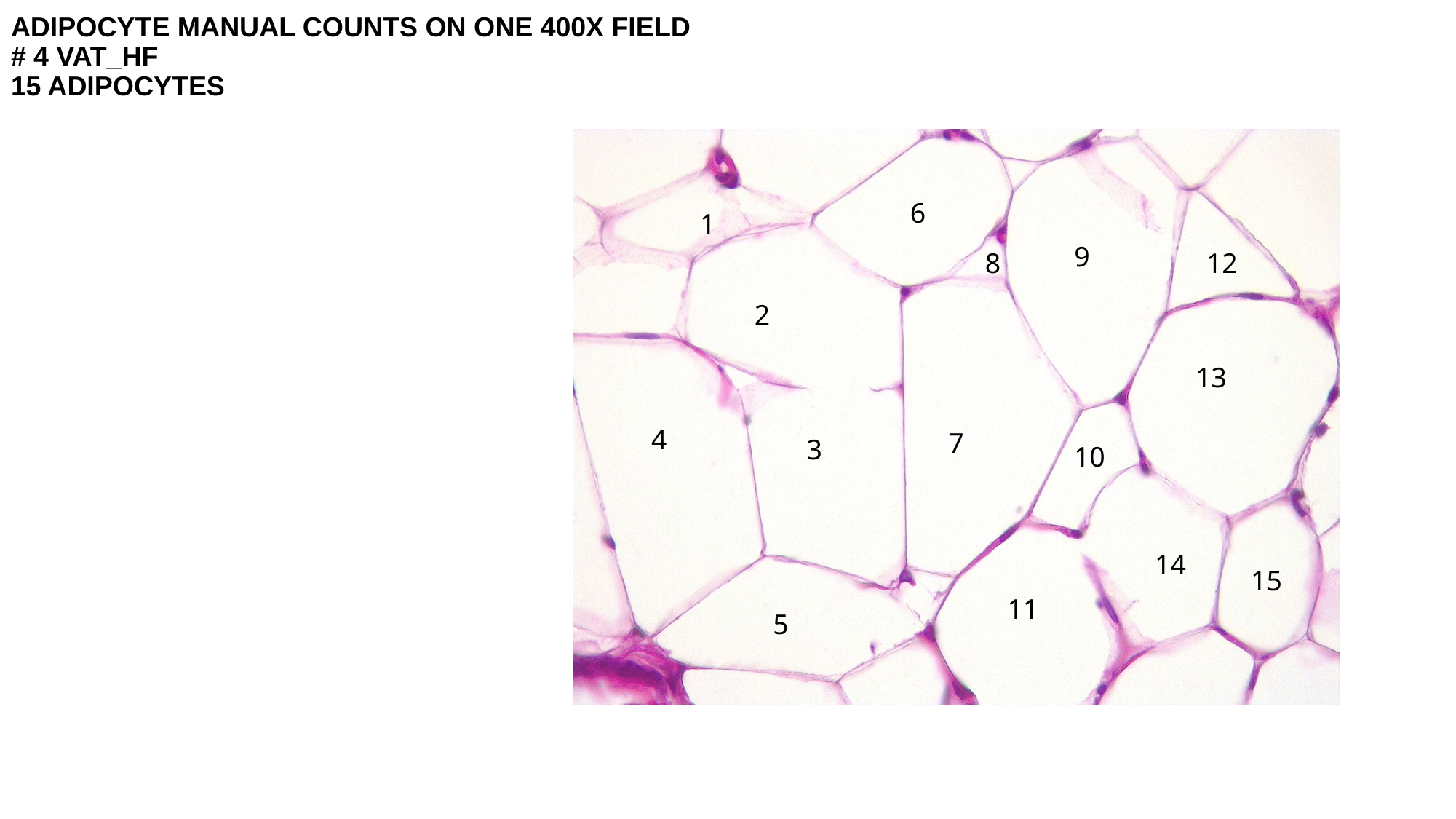

# ADIPOCYTE MANUAL COUNTS ON ONE 400X FIELD # 4 VAT_HF 15 ADIPOCYTES
6
1
9
8
12
2
13
4
7
3
10
14
15
11
5

## Slide 5
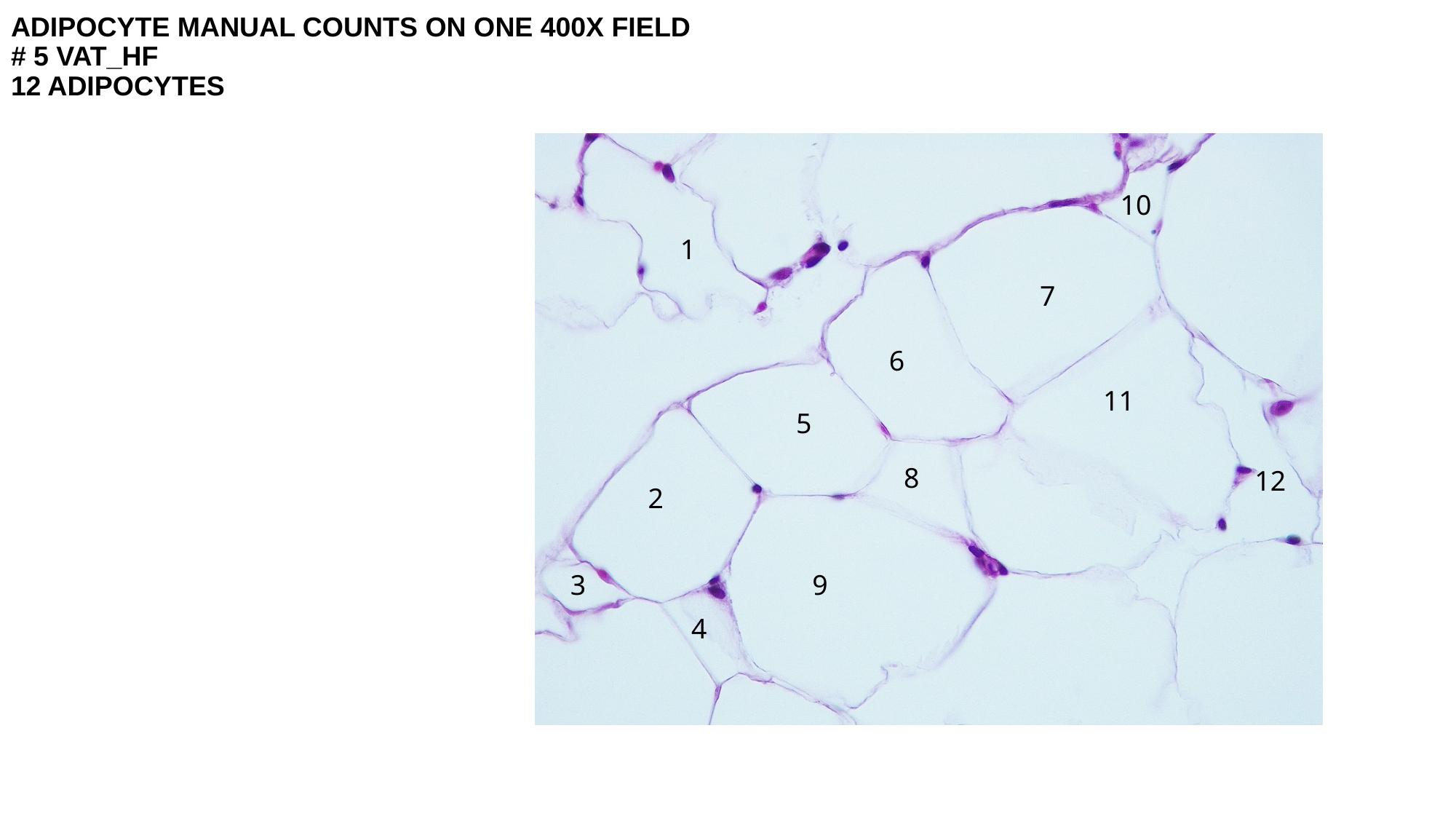

# ADIPOCYTE MANUAL COUNTS ON ONE 400X FIELD # 5 VAT_HF 12 ADIPOCYTES
10
1
7
6
11
5
8
12
2
3
9
4
