# Supplementary material for: Inhibition of DPP-4 Attenuates Endotoxemia-Induced NLRC4 Inflammasome and Inflammation in Visceral Adipose Tissue of Mice Fed a High-Fat Diet
Source: Biomolecules. 2025 Feb 25;15(3):333. doi: 10.3390/biom15030333 (PMC11940500; doi:10.3390/biom15030333)
Supplement: Supplementary file 1 [file biomolecules-15-00333-s001.zip › Supplementary file 5 VAT_HFL.pptx]

## Slide 1
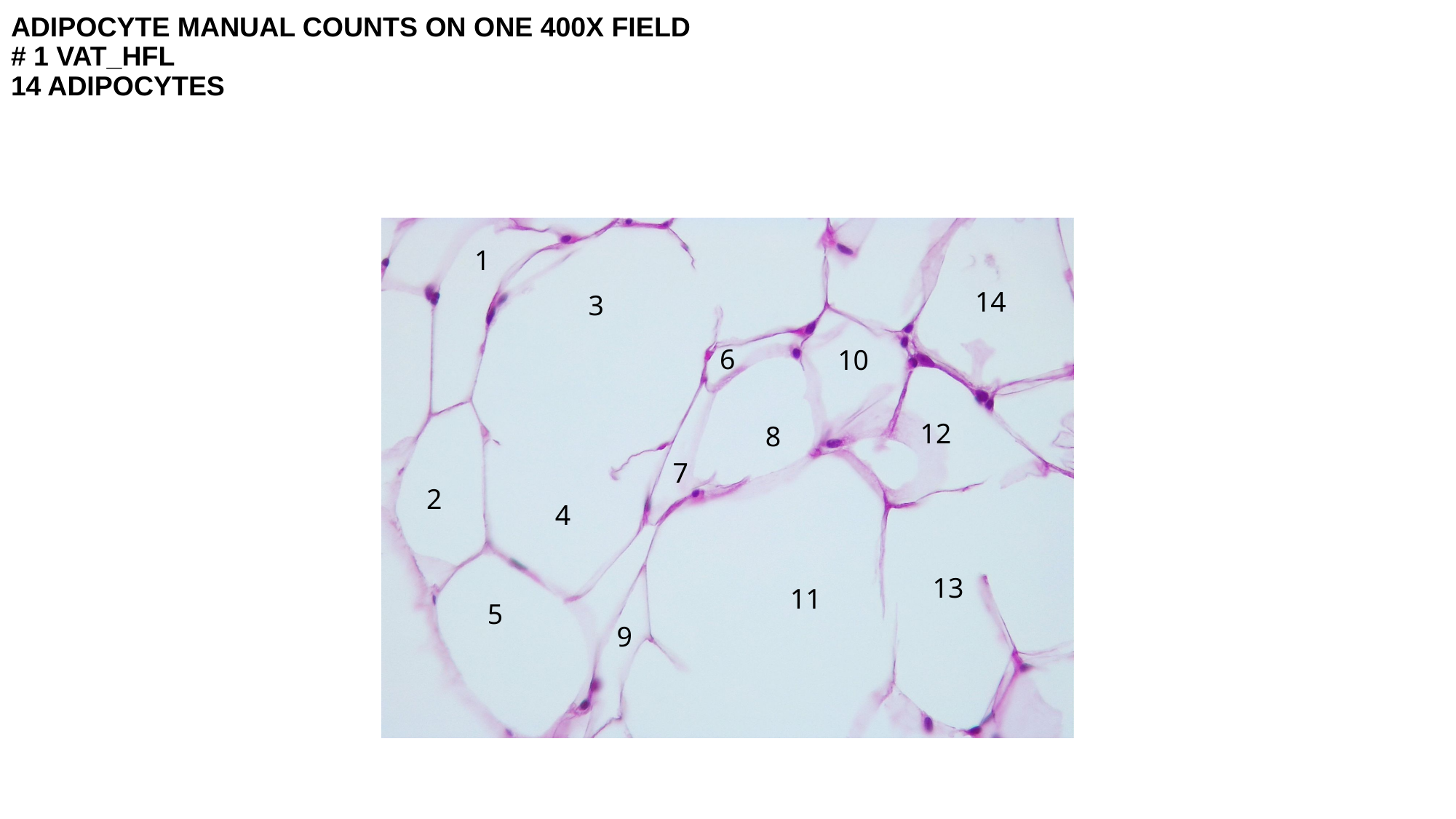

# ADIPOCYTE MANUAL COUNTS ON ONE 400X FIELD # 1 VAT_HFL 14 ADIPOCYTES
1
14
3
6
10
12
8
7
2
4
13
11
5
9

## Slide 2
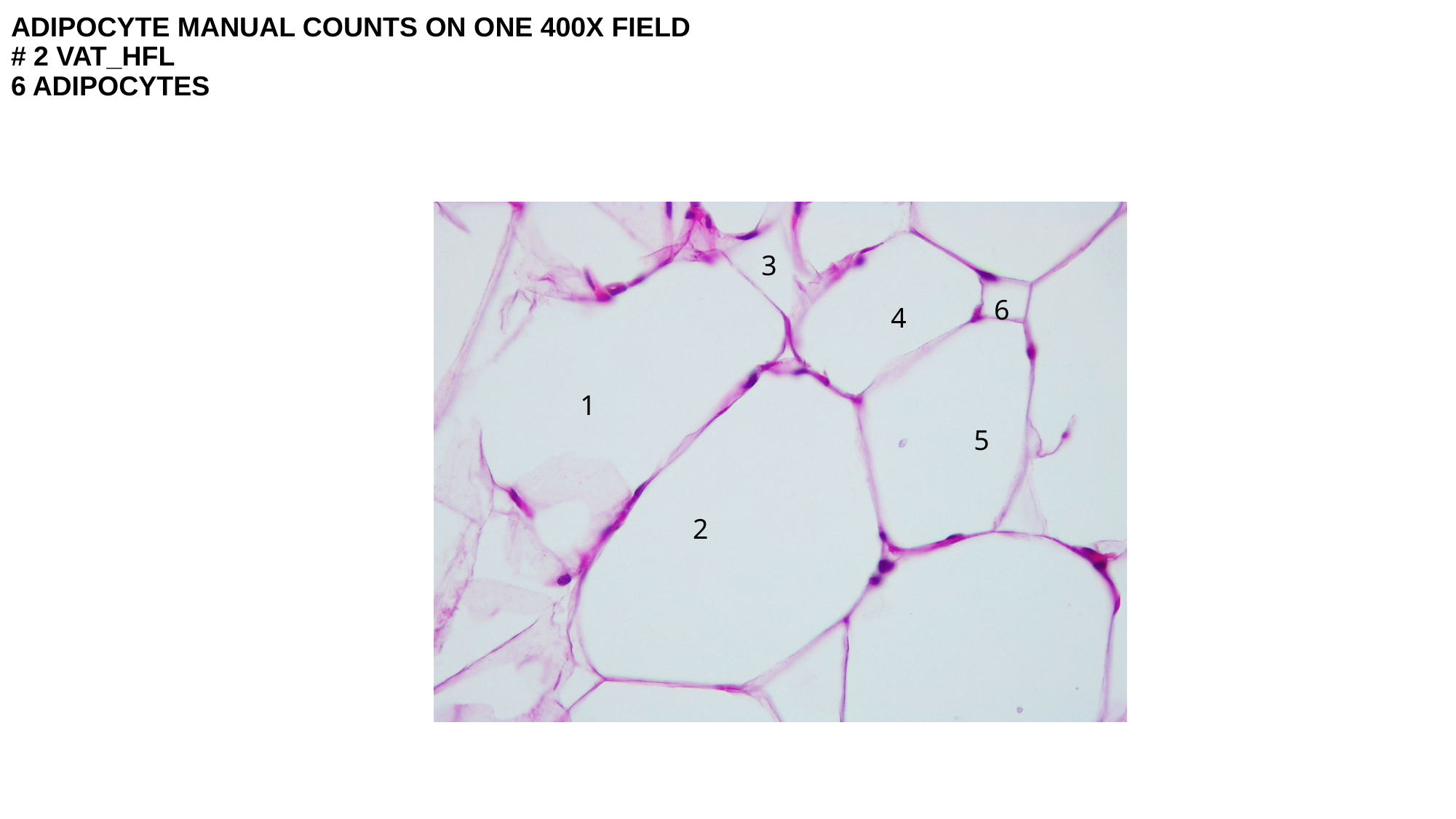

# ADIPOCYTE MANUAL COUNTS ON ONE 400X FIELD # 2 VAT_HFL 6 ADIPOCYTES
3
6
4
1
5
2

## Slide 3
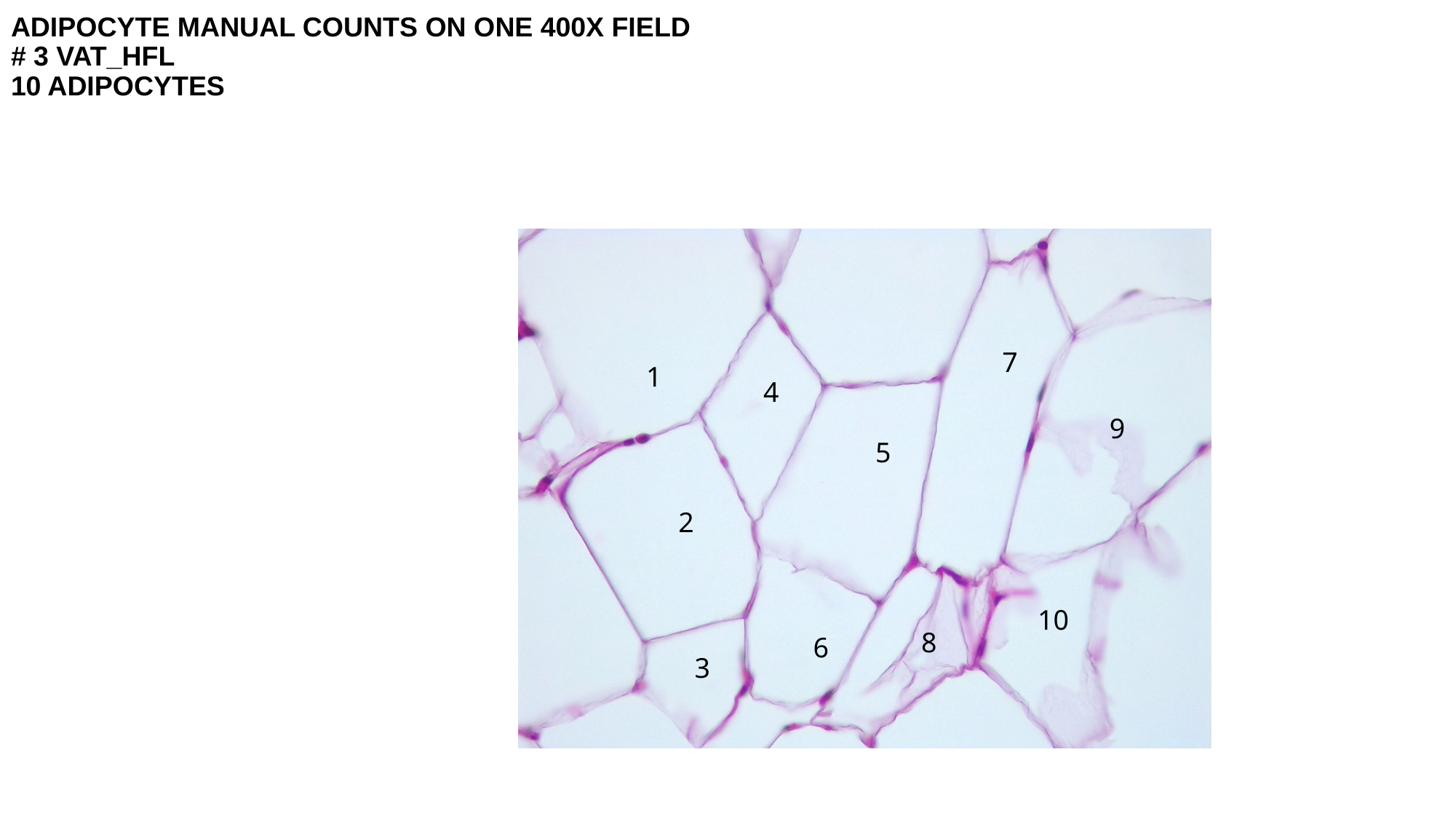

# ADIPOCYTE MANUAL COUNTS ON ONE 400X FIELD # 3 VAT_HFL 10 ADIPOCYTES
7
1
4
9
5
2
10
8
6
3

## Slide 4
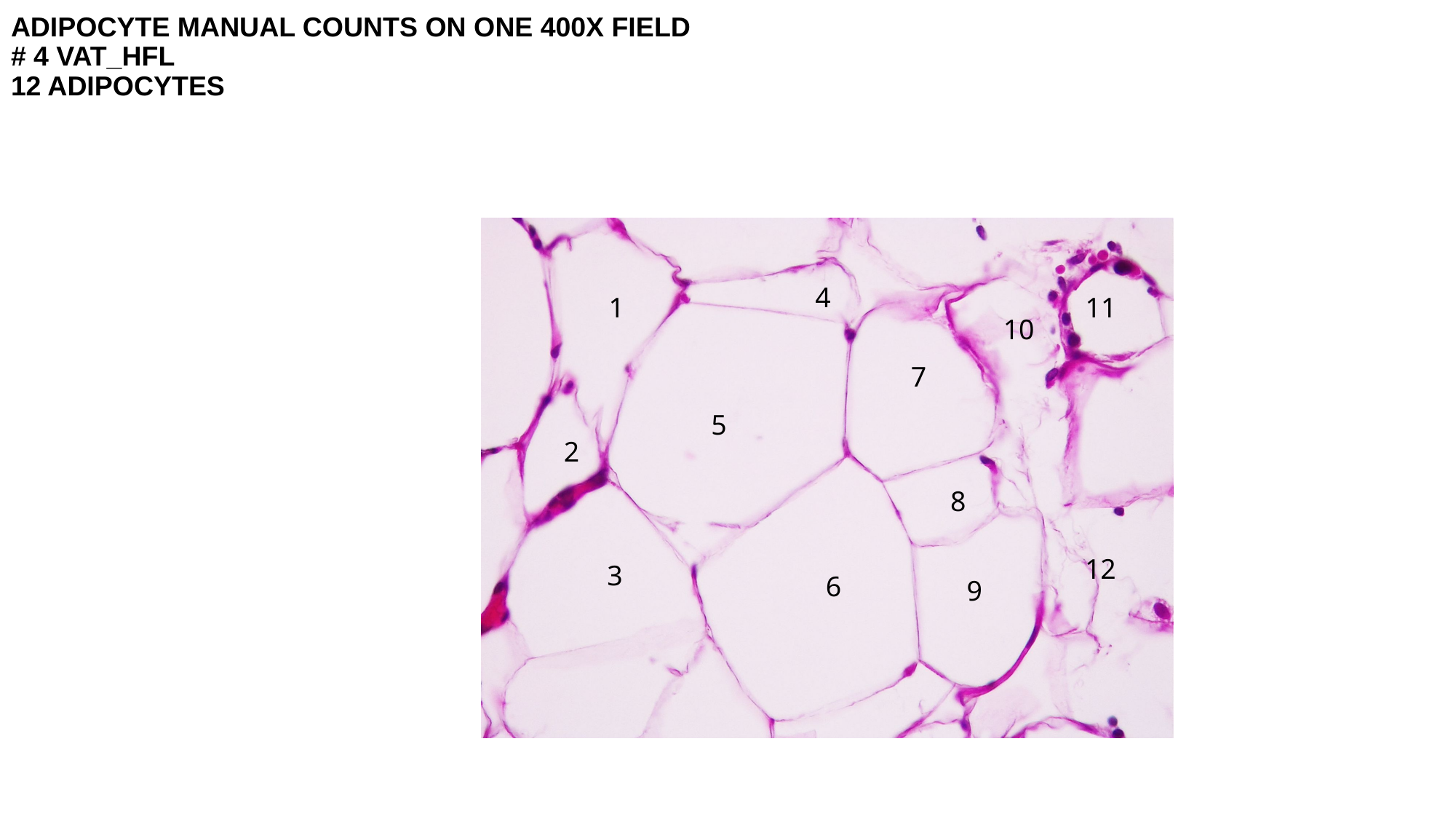

# ADIPOCYTE MANUAL COUNTS ON ONE 400X FIELD # 4 VAT_HFL 12 ADIPOCYTES
4
1
11
10
7
5
2
8
12
3
6
9

## Slide 5
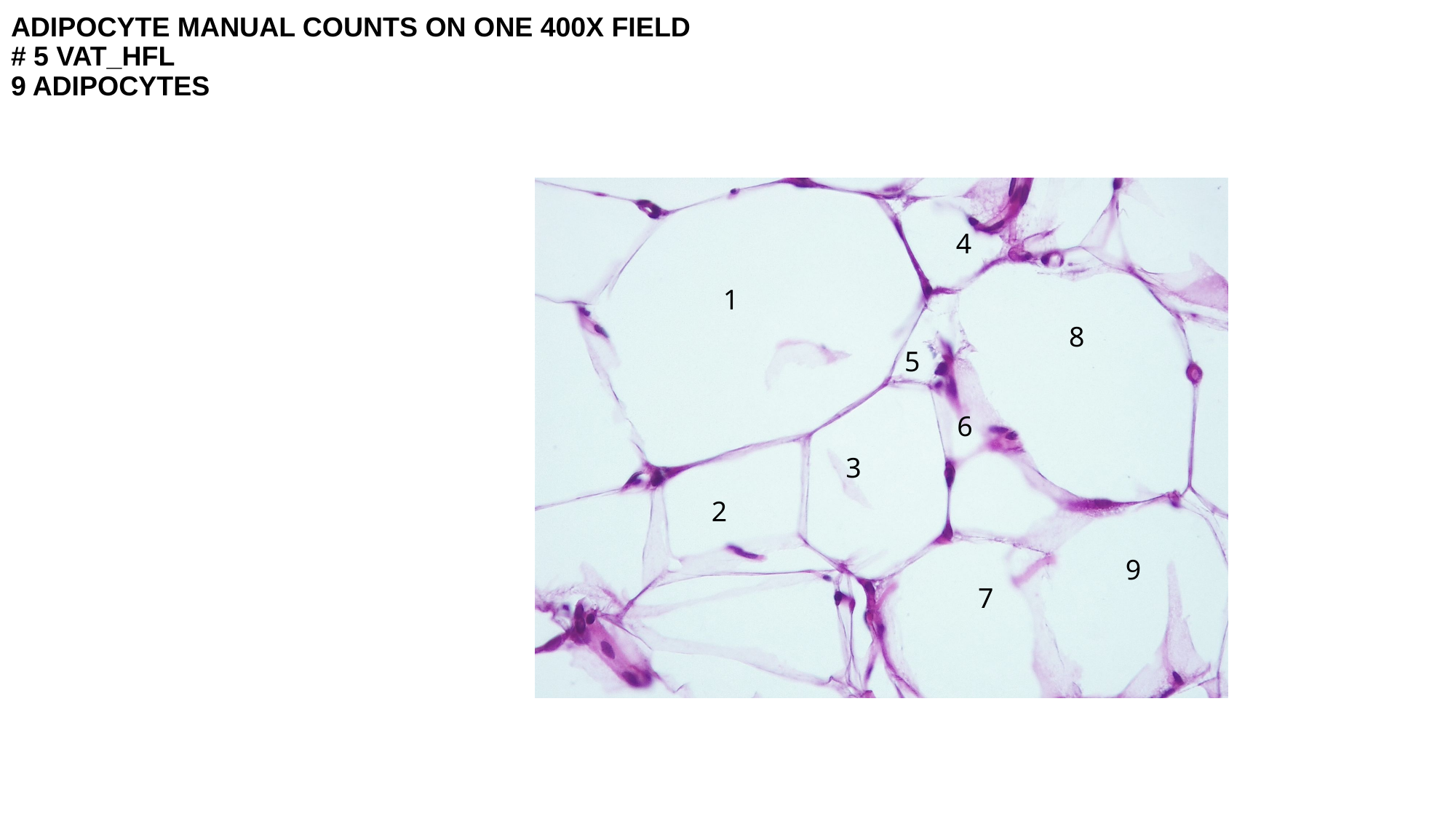

# ADIPOCYTE MANUAL COUNTS ON ONE 400X FIELD # 5 VAT_HFL 9 ADIPOCYTES
4
1
8
5
6
3
2
9
7

## Slide 6
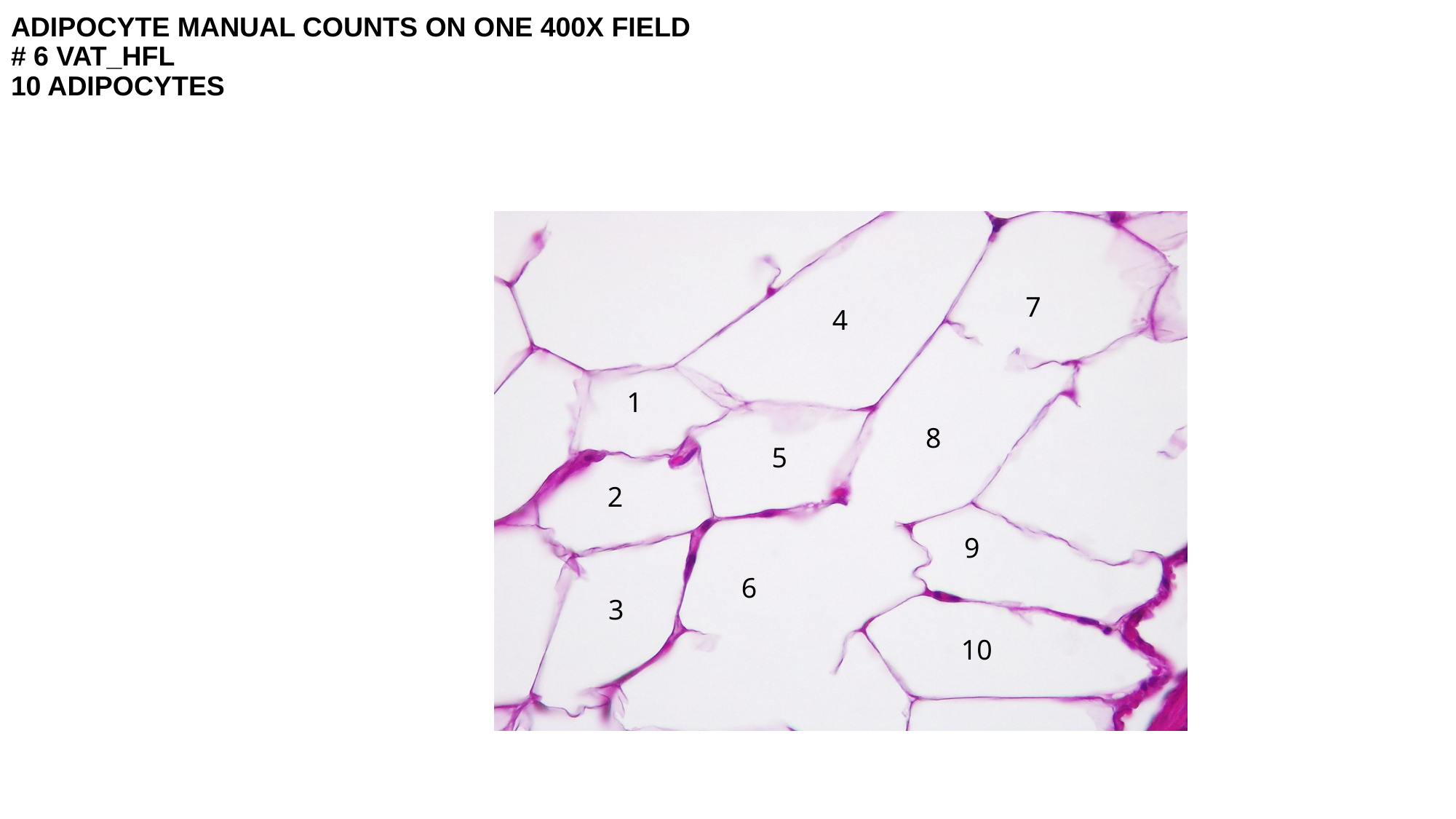

# ADIPOCYTE MANUAL COUNTS ON ONE 400X FIELD # 6 VAT_HFL 10 ADIPOCYTES
7
4
1
8
5
2
9
6
3
10

## Slide 7
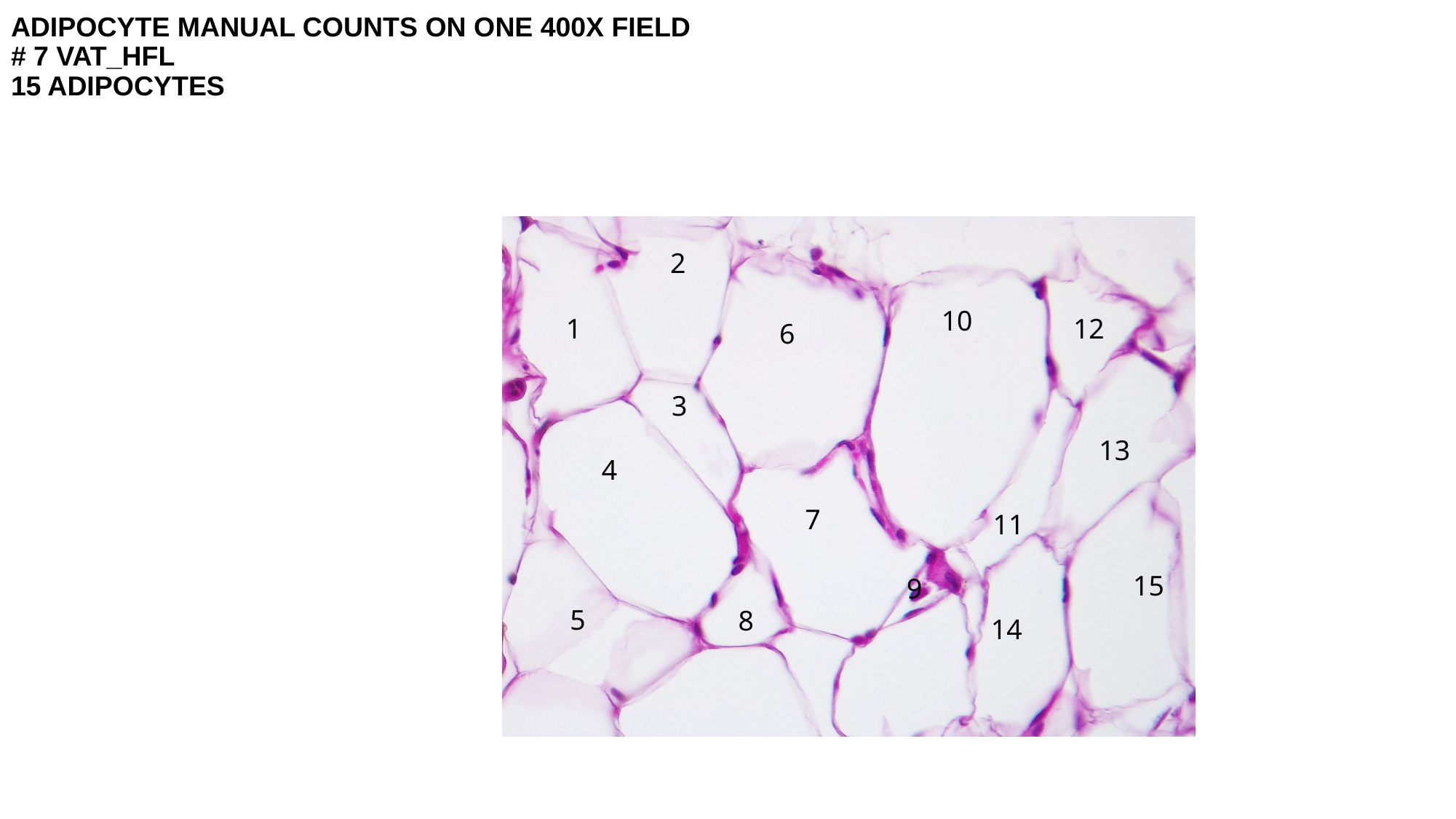

# ADIPOCYTE MANUAL COUNTS ON ONE 400X FIELD # 7 VAT_HFL 15 ADIPOCYTES
2
10
1
12
6
3
13
4
7
11
15
9
5
8
14

## Slide 8
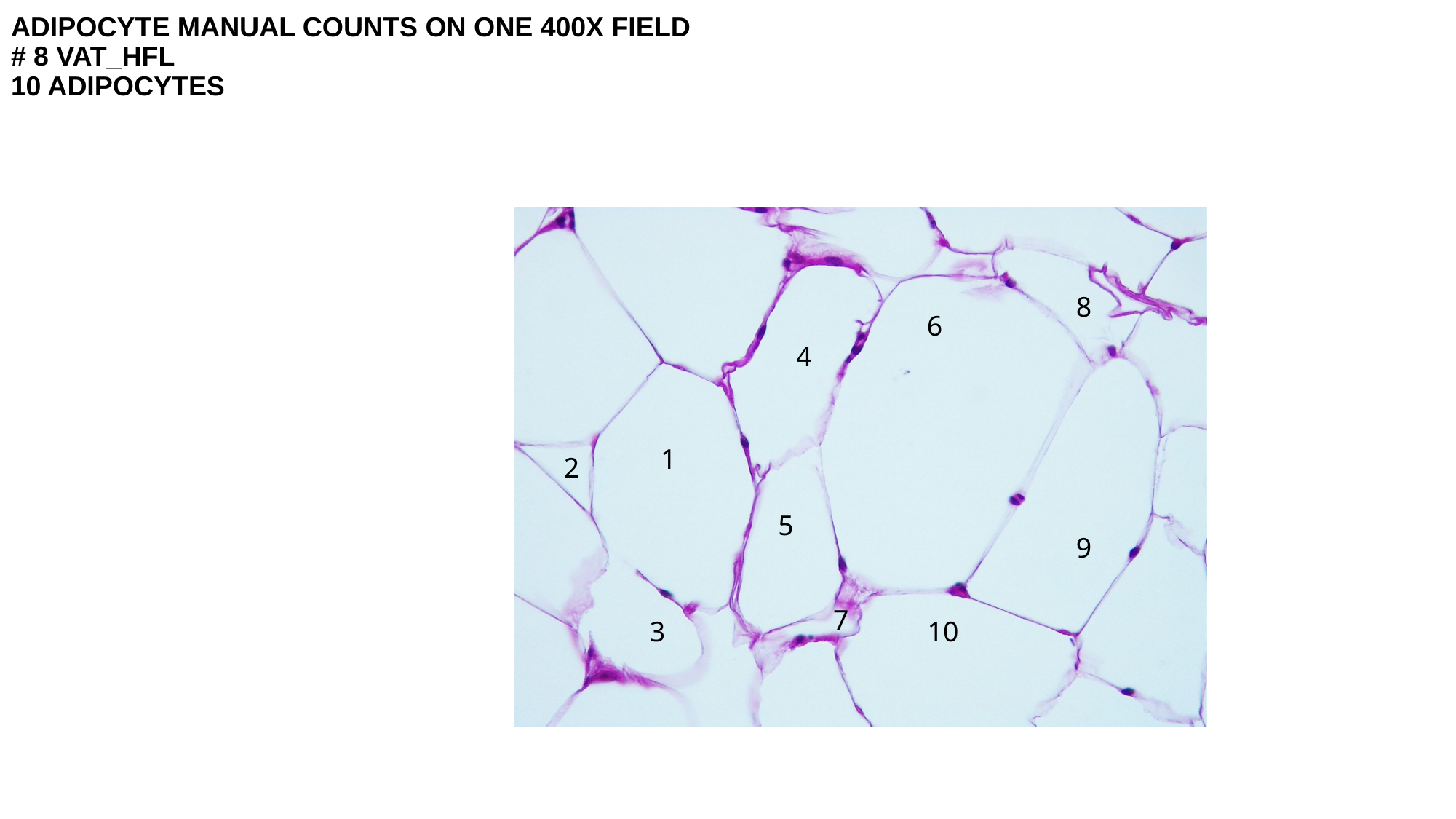

# ADIPOCYTE MANUAL COUNTS ON ONE 400X FIELD # 8 VAT_HFL 10 ADIPOCYTES
8
6
4
1
2
5
9
7
3
10
