# Supplementary material for: Inhibition of DPP-4 Attenuates Endotoxemia-Induced NLRC4 Inflammasome and Inflammation in Visceral Adipose Tissue of Mice Fed a High-Fat Diet
Source: Biomolecules. 2025 Feb 25;15(3):333. doi: 10.3390/biom15030333 (PMC11940500; doi:10.3390/biom15030333)
Supplement: Supplementary file 1 [file biomolecules-15-00333-s001.zip › Supplementary file 6 SAT_NC.pptx]

## Slide 1
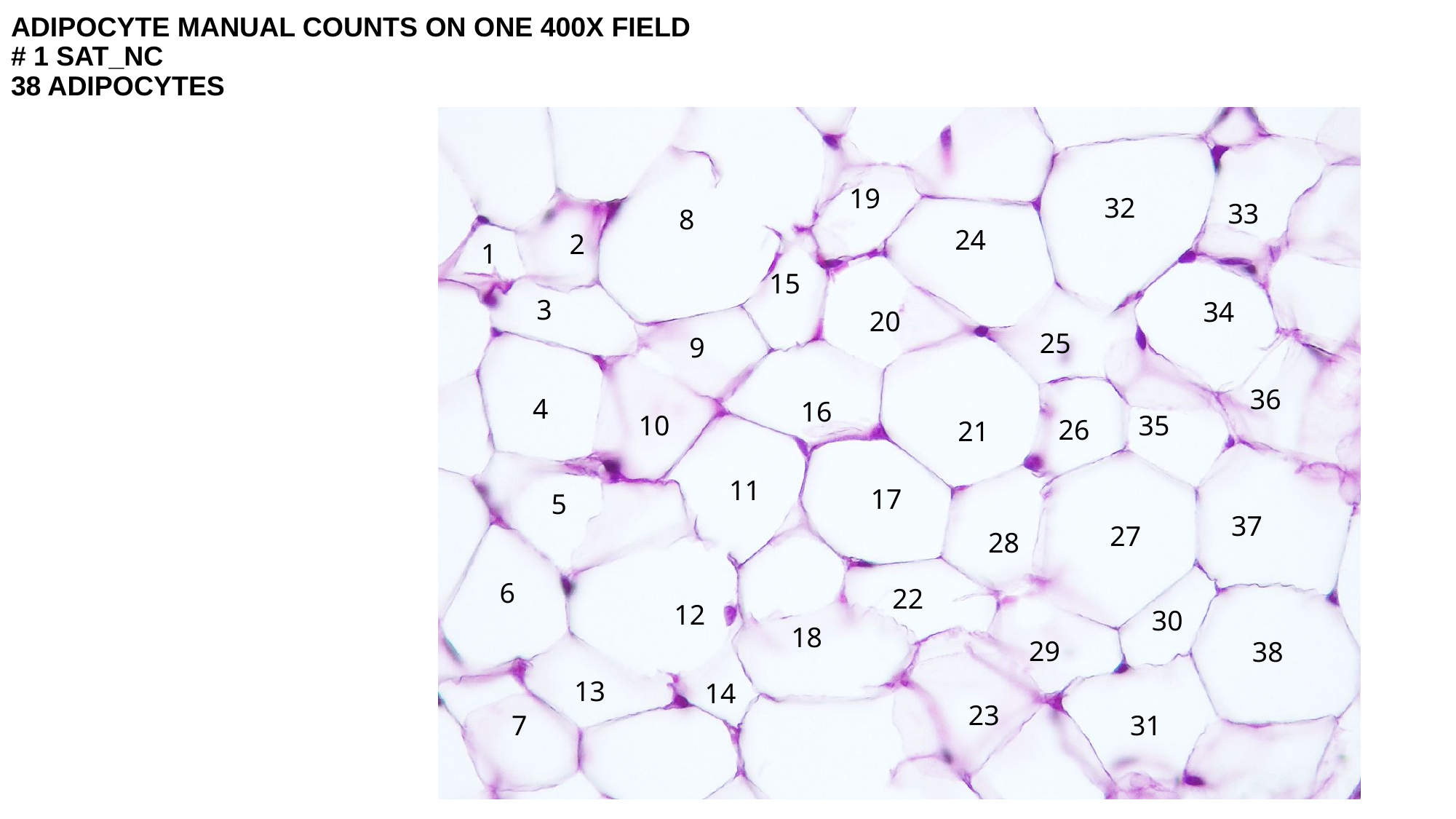

# ADIPOCYTE MANUAL COUNTS ON ONE 400X FIELD # 1 SAT_NC 38 ADIPOCYTES
19
32
33
8
24
2
1
15
3
34
20
25
9
36
4
16
10
35
26
21
11
17
5
37
27
28
6
22
12
30
18
29
38
13
14
23
31
7

## Slide 2
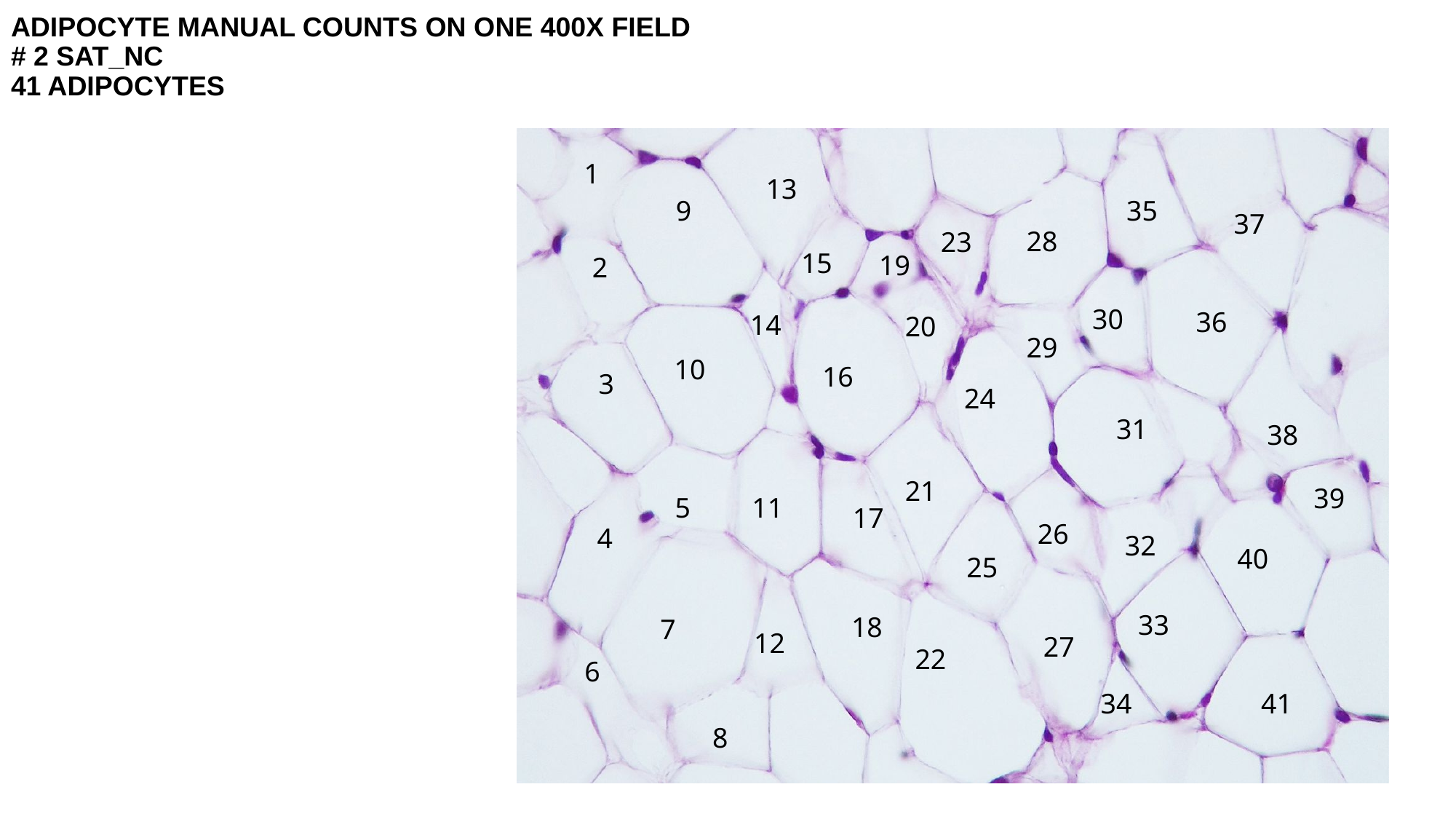

# ADIPOCYTE MANUAL COUNTS ON ONE 400X FIELD # 2 SAT_NC 41 ADIPOCYTES
1
13
9
35
37
28
23
15
19
2
30
36
14
20
29
10
16
3
24
31
38
21
39
5
11
17
26
4
32
40
25
33
18
7
12
27
22
6
34
41
8

## Slide 3
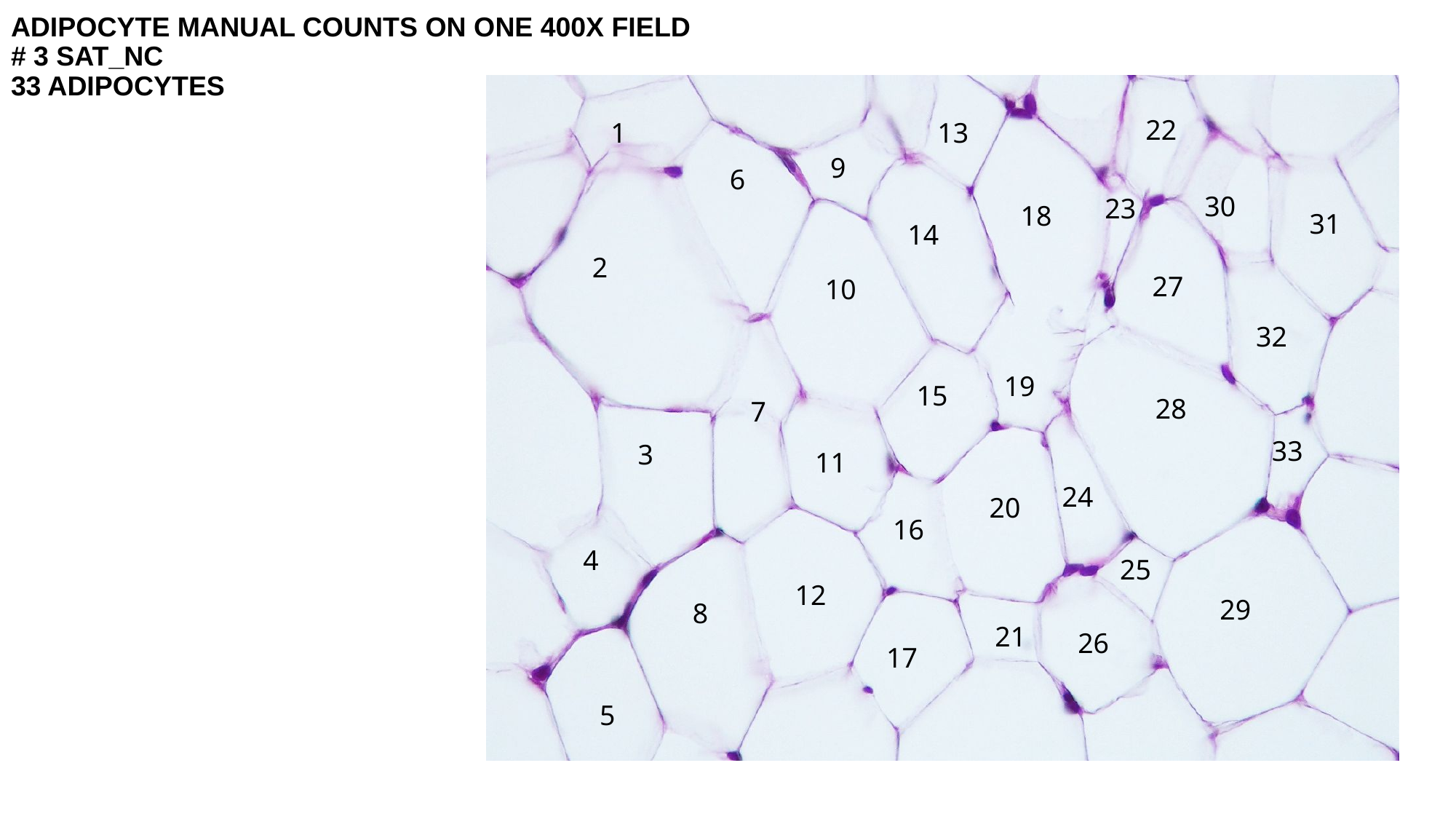

# ADIPOCYTE MANUAL COUNTS ON ONE 400X FIELD # 3 SAT_NC 33 ADIPOCYTES
22
1
13
9
6
30
23
18
31
14
2
27
10
32
19
15
28
7
33
3
11
24
20
16
4
25
12
29
8
21
26
17
5

## Slide 4
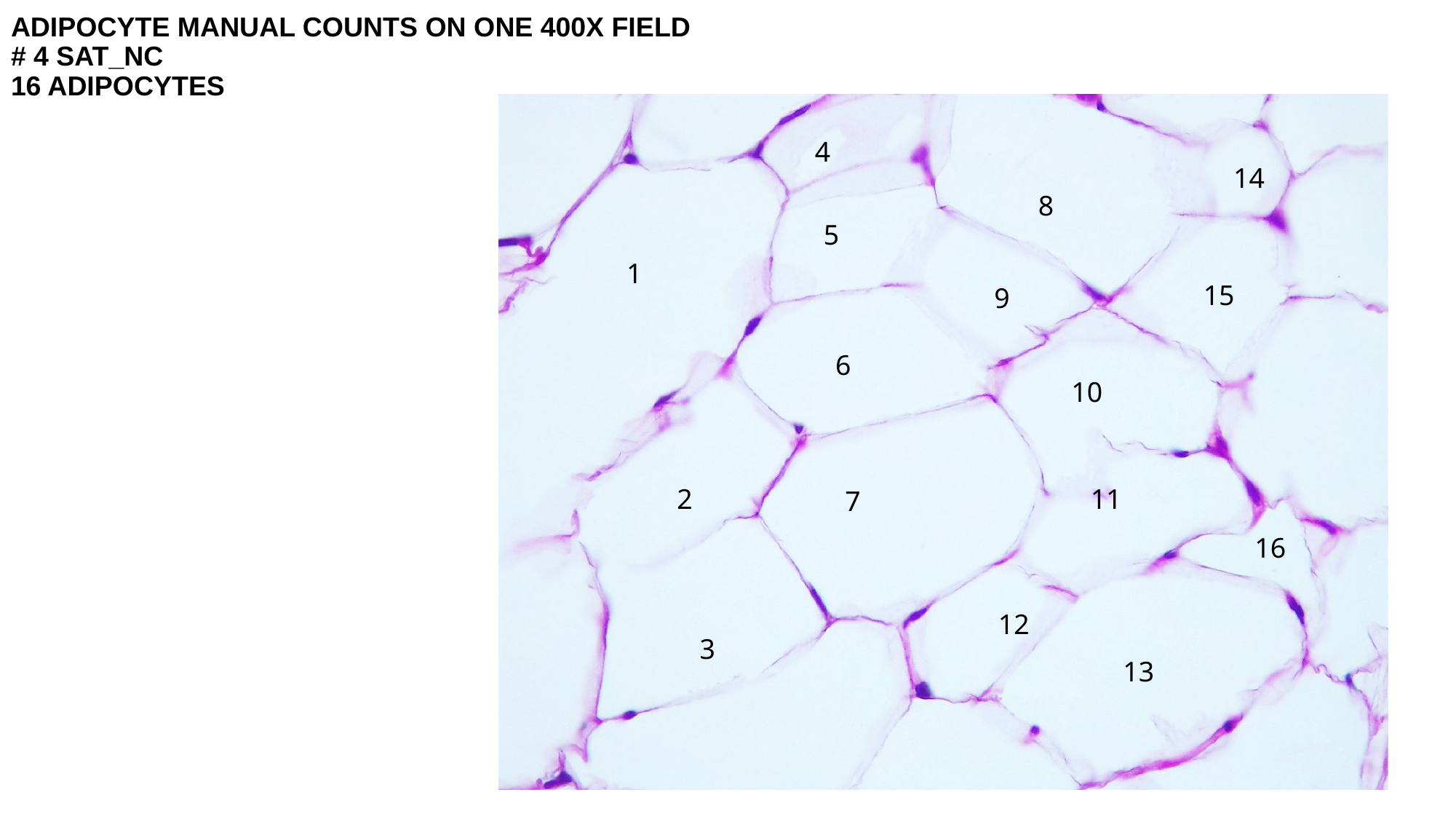

# ADIPOCYTE MANUAL COUNTS ON ONE 400X FIELD # 4 SAT_NC 16 ADIPOCYTES
4
14
8
5
1
15
9
6
10
2
11
7
16
12
3
13

## Slide 5
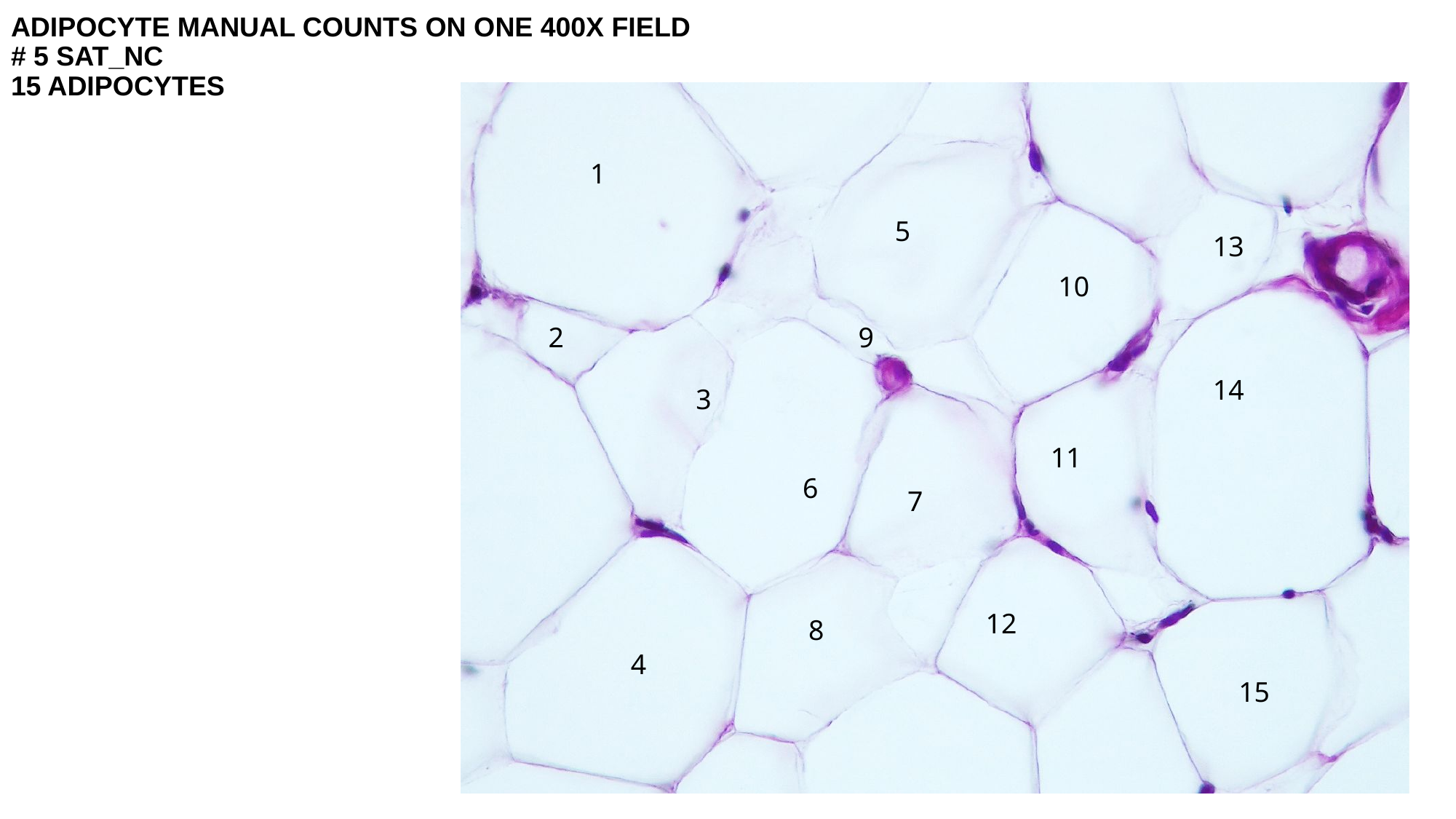

# ADIPOCYTE MANUAL COUNTS ON ONE 400X FIELD # 5 SAT_NC 15 ADIPOCYTES
1
5
13
10
2
9
14
3
11
6
7
12
8
4
15

## Slide 6
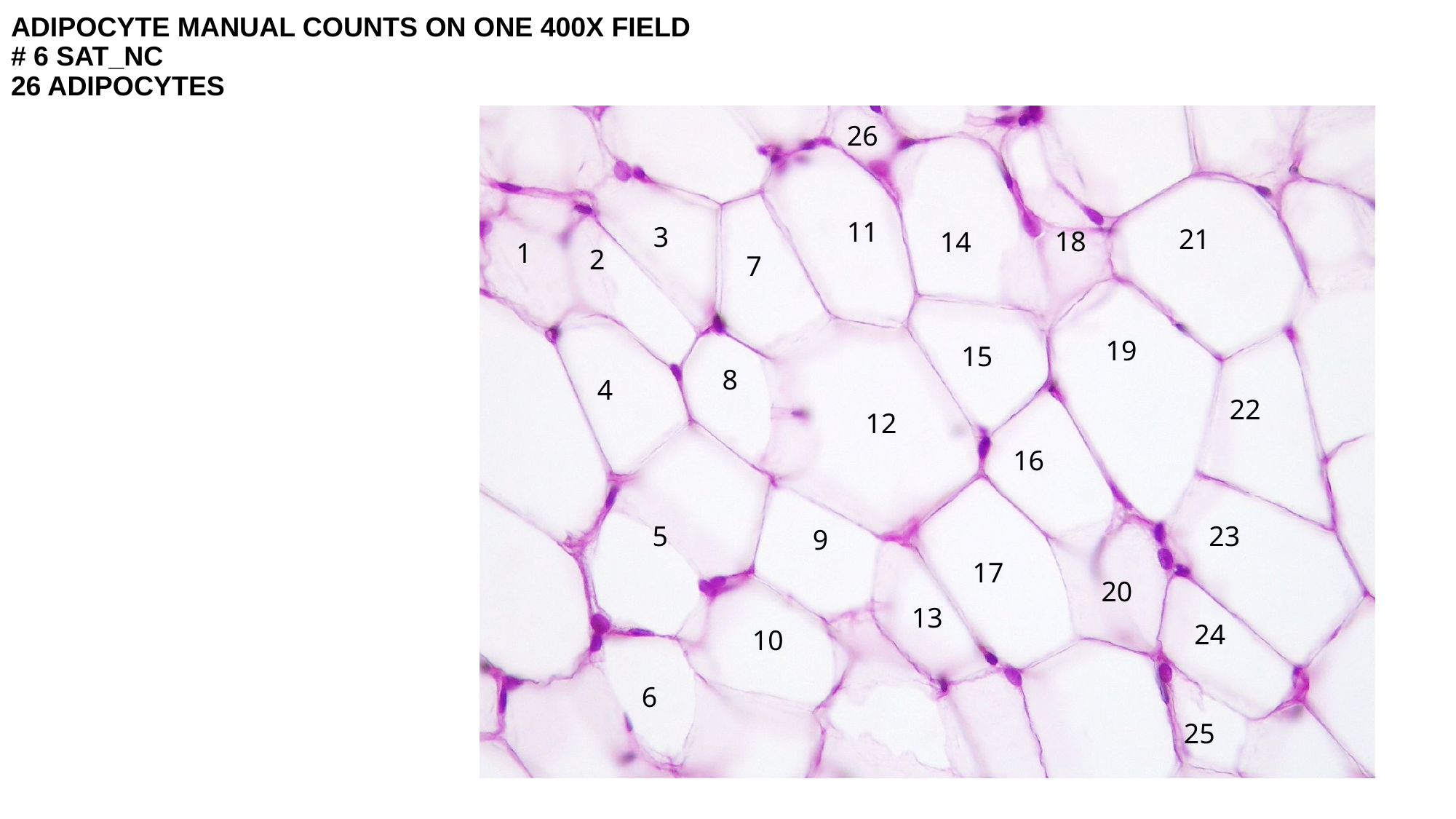

# ADIPOCYTE MANUAL COUNTS ON ONE 400X FIELD # 6 SAT_NC 26 ADIPOCYTES
26
11
3
21
18
14
1
2
7
19
15
8
4
22
12
16
5
23
9
17
20
13
24
10
6
25

## Slide 7
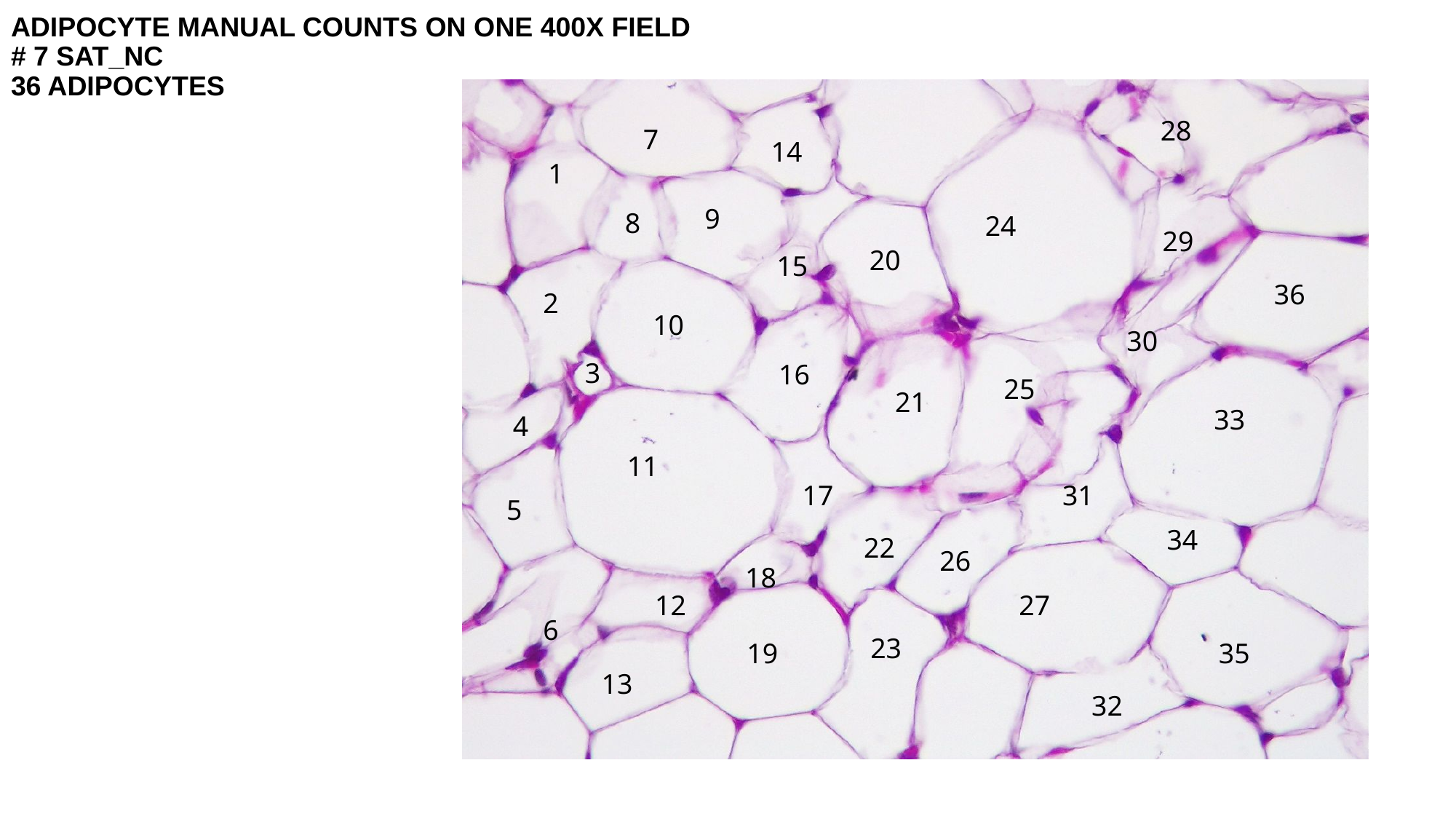

# ADIPOCYTE MANUAL COUNTS ON ONE 400X FIELD # 7 SAT_NC 36 ADIPOCYTES
28
7
14
1
9
8
24
29
20
15
36
2
10
30
3
16
25
21
33
4
11
31
17
5
34
22
26
18
27
12
6
23
19
35
13
32

## Slide 8
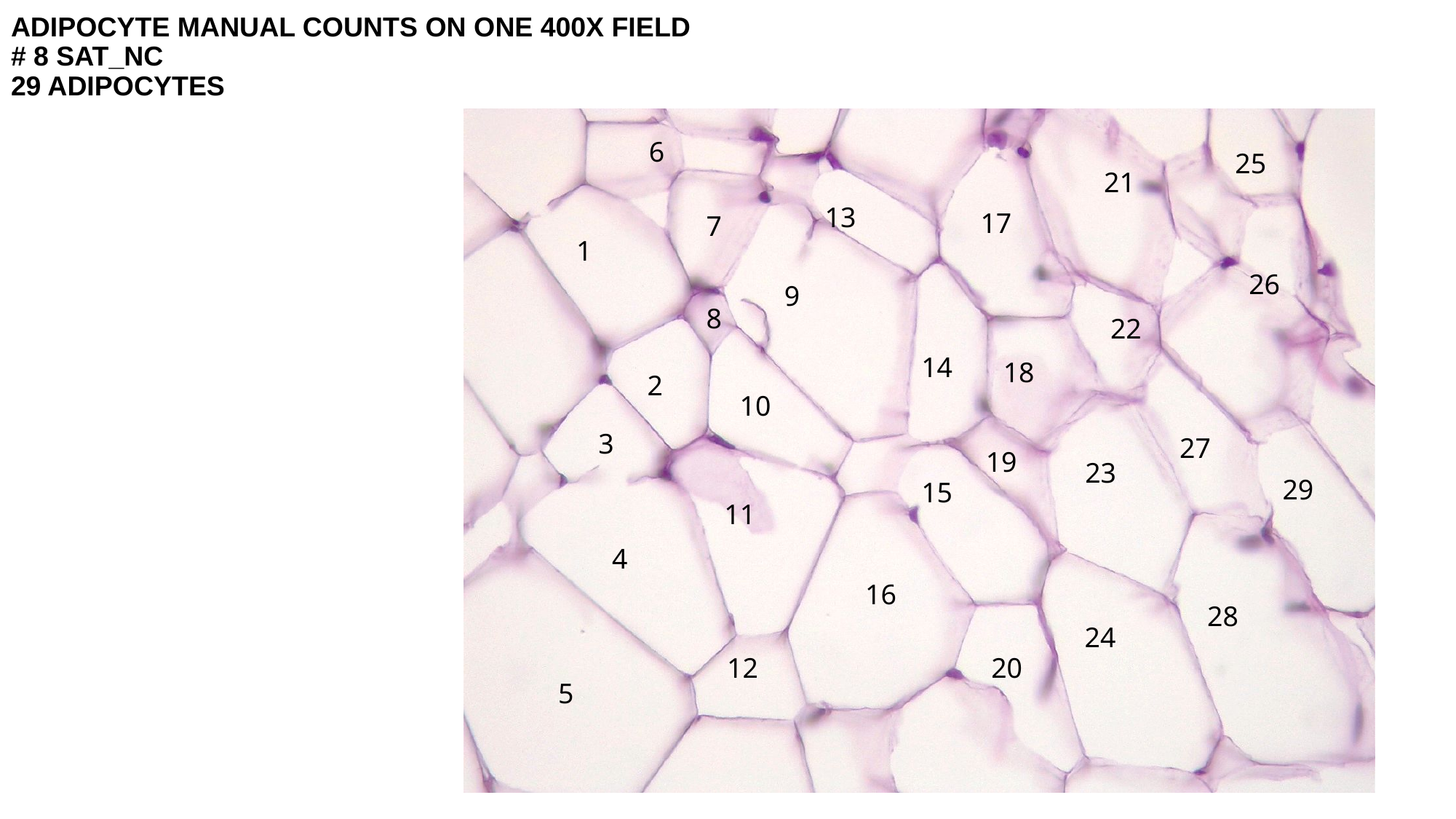

# ADIPOCYTE MANUAL COUNTS ON ONE 400X FIELD # 8 SAT_NC 29 ADIPOCYTES
6
25
21
13
17
7
1
26
9
8
22
14
18
2
10
3
27
19
23
29
15
11
4
16
28
24
12
20
5
