# Supplementary material for: Inhibition of DPP-4 Attenuates Endotoxemia-Induced NLRC4 Inflammasome and Inflammation in Visceral Adipose Tissue of Mice Fed a High-Fat Diet
Source: Biomolecules. 2025 Feb 25;15(3):333. doi: 10.3390/biom15030333 (PMC11940500; doi:10.3390/biom15030333)
Supplement: Supplementary file 1 [file biomolecules-15-00333-s001.zip › Supplementary file 7 SAT_HF.pptx]

## Slide 1
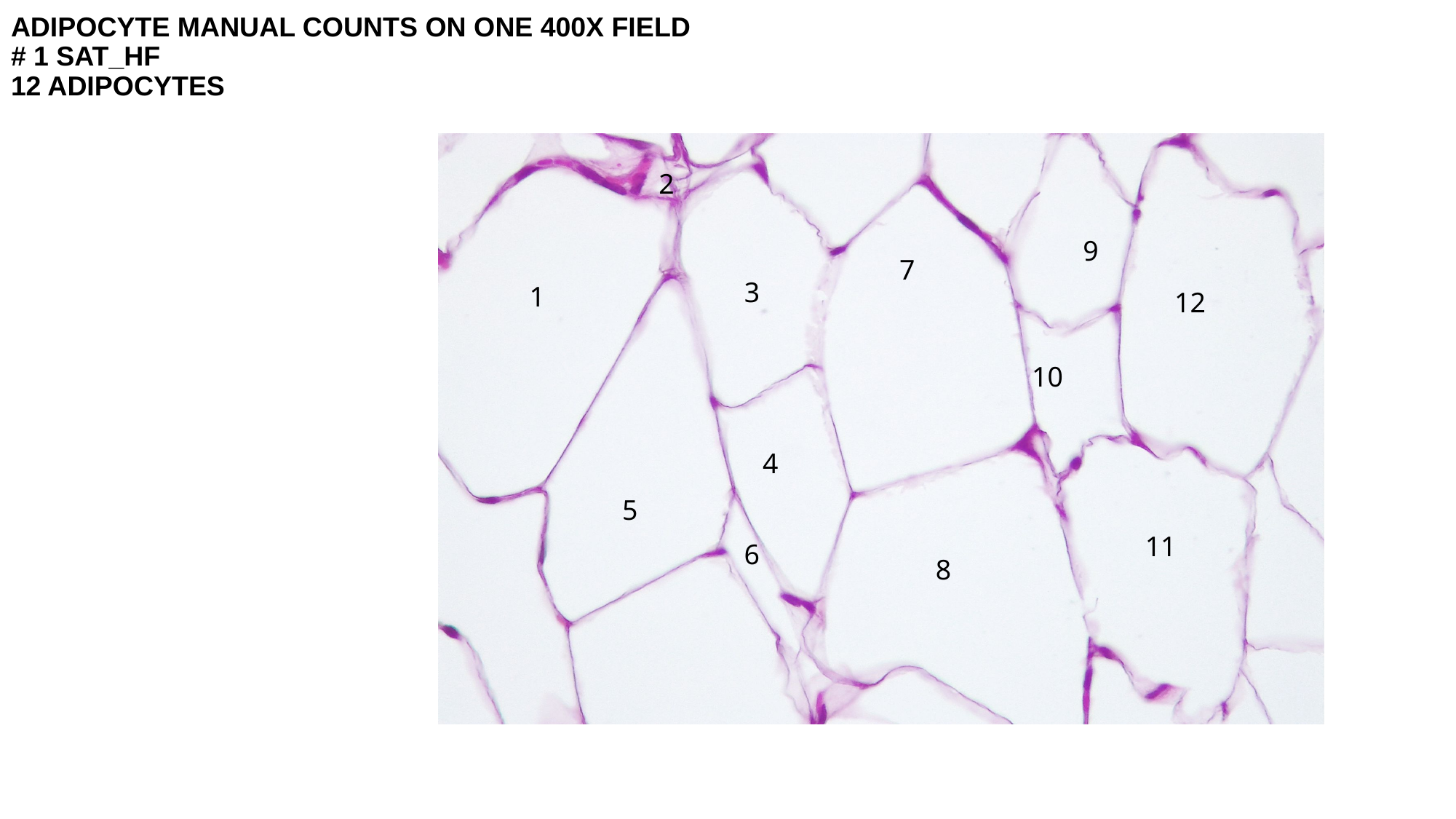

# ADIPOCYTE MANUAL COUNTS ON ONE 400X FIELD # 1 SAT_HF 12 ADIPOCYTES
2
9
7
3
1
12
10
4
5
11
6
8

## Slide 2
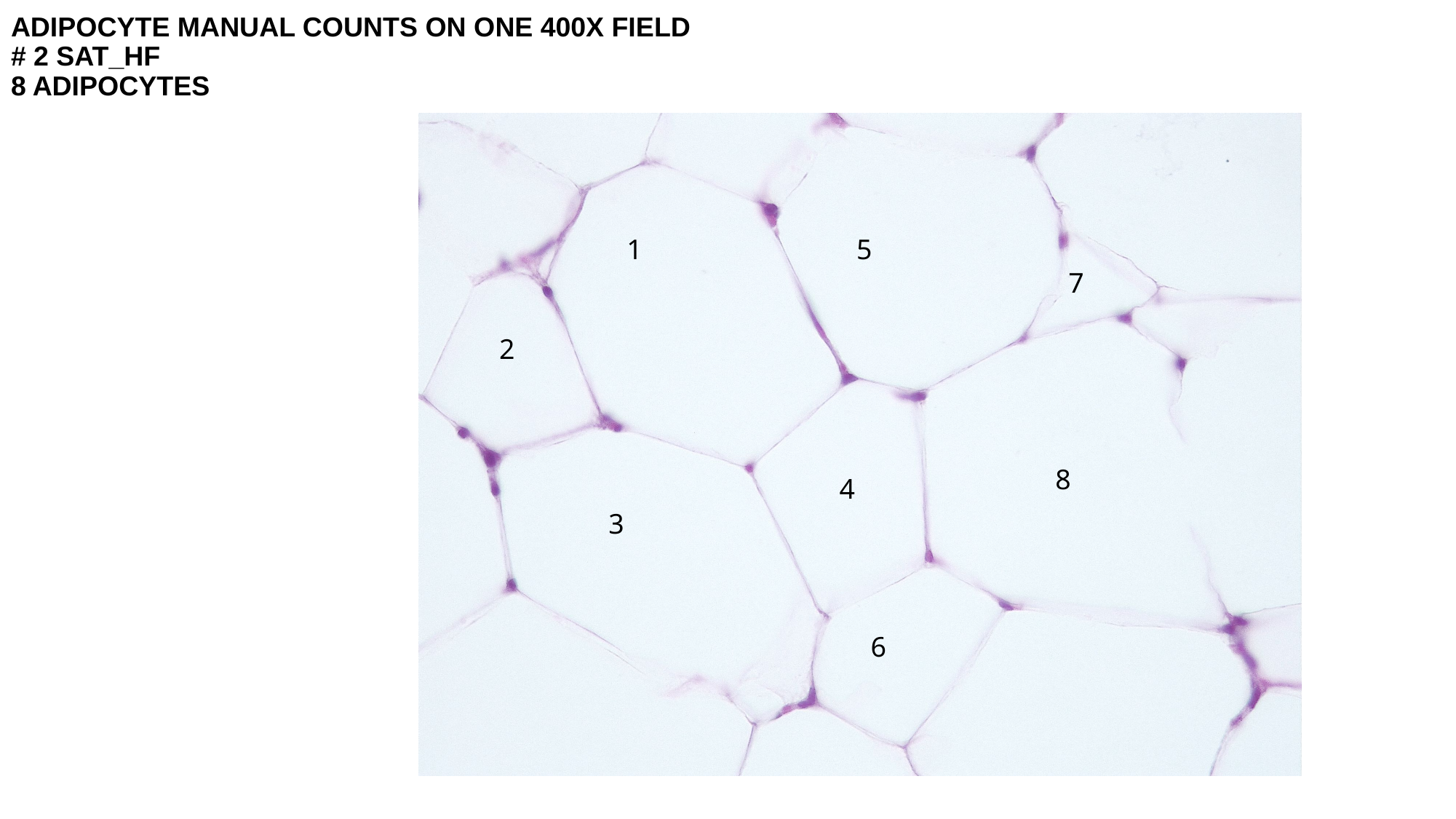

# ADIPOCYTE MANUAL COUNTS ON ONE 400X FIELD # 2 SAT_HF 8 ADIPOCYTES
1
5
7
2
8
4
3
6

## Slide 3
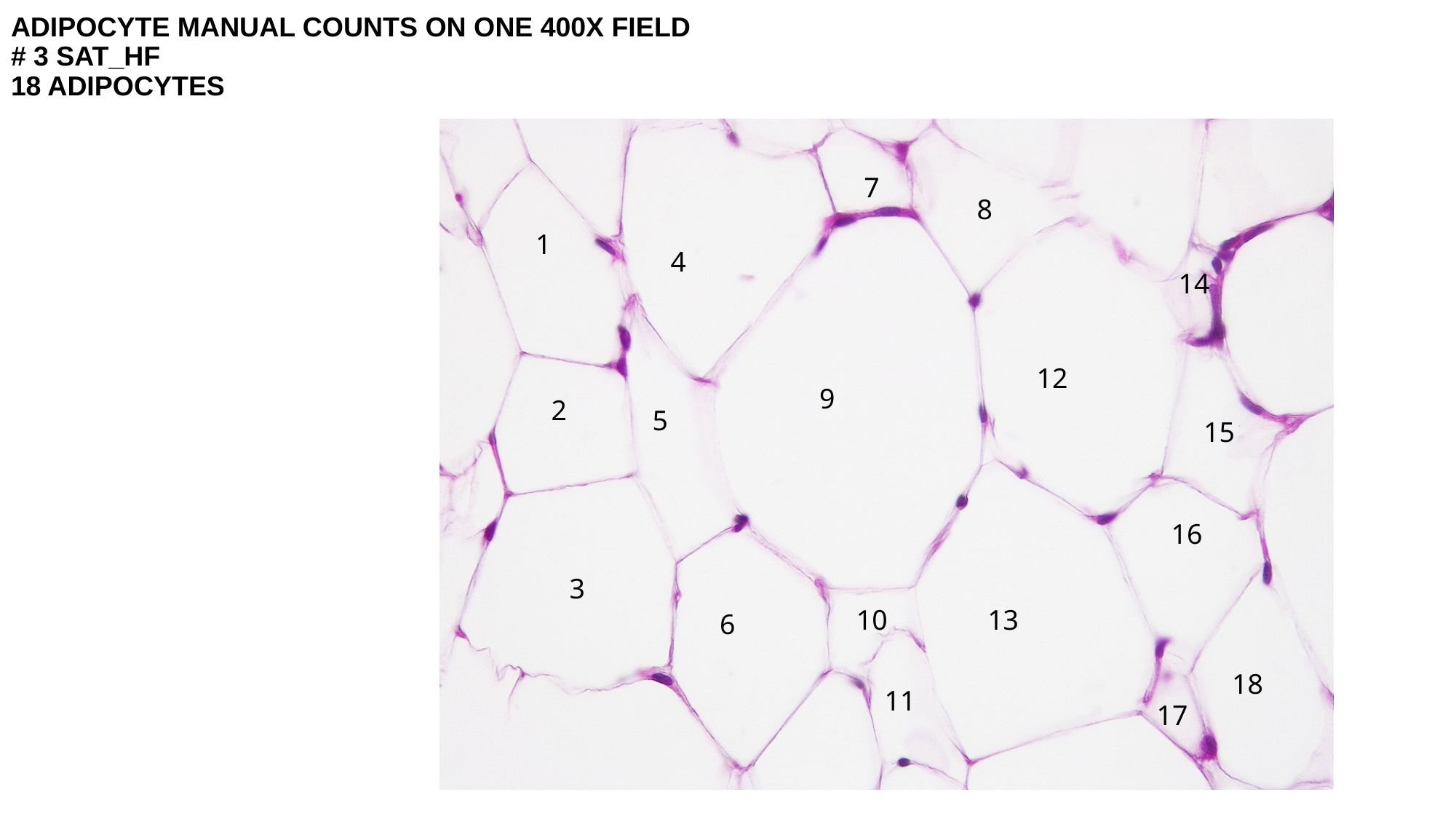

# ADIPOCYTE MANUAL COUNTS ON ONE 400X FIELD # 3 SAT_HF 18 ADIPOCYTES
7
8
1
4
14
12
9
2
5
15
16
3
10
13
6
18
11
17

## Slide 4
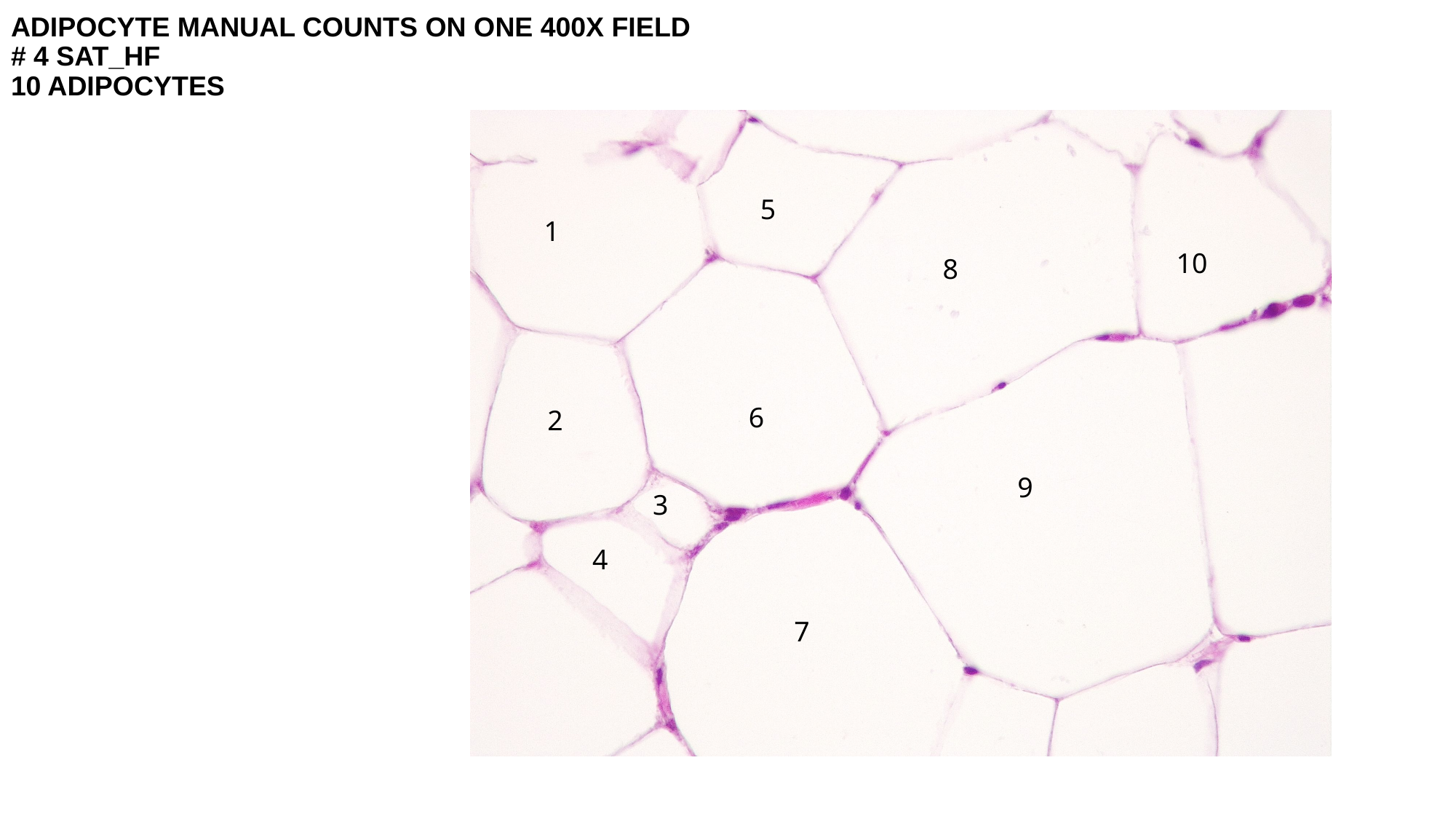

# ADIPOCYTE MANUAL COUNTS ON ONE 400X FIELD # 4 SAT_HF 10 ADIPOCYTES
5
1
10
8
6
2
9
3
4
7

## Slide 5
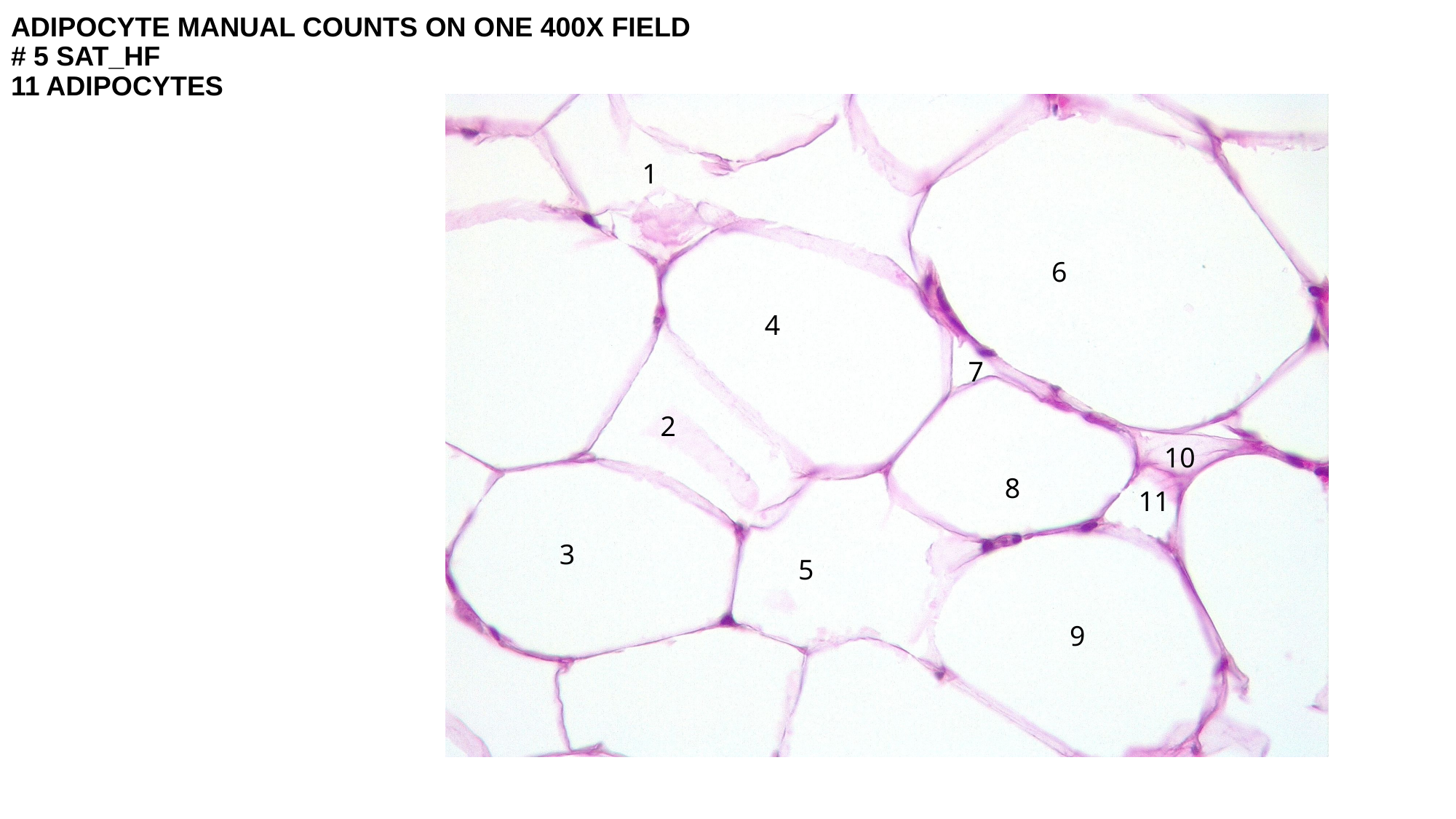

# ADIPOCYTE MANUAL COUNTS ON ONE 400X FIELD # 5 SAT_HF 11 ADIPOCYTES
1
6
4
7
2
10
8
11
3
5
9
