# Supplementary material for: Inhibition of DPP-4 Attenuates Endotoxemia-Induced NLRC4 Inflammasome and Inflammation in Visceral Adipose Tissue of Mice Fed a High-Fat Diet
Source: Biomolecules. 2025 Feb 25;15(3):333. doi: 10.3390/biom15030333 (PMC11940500; doi:10.3390/biom15030333)
Supplement: Supplementary file 1 [file biomolecules-15-00333-s001.zip › Supplementary file 8 SAT-HFL.pptx]

## Slide 1
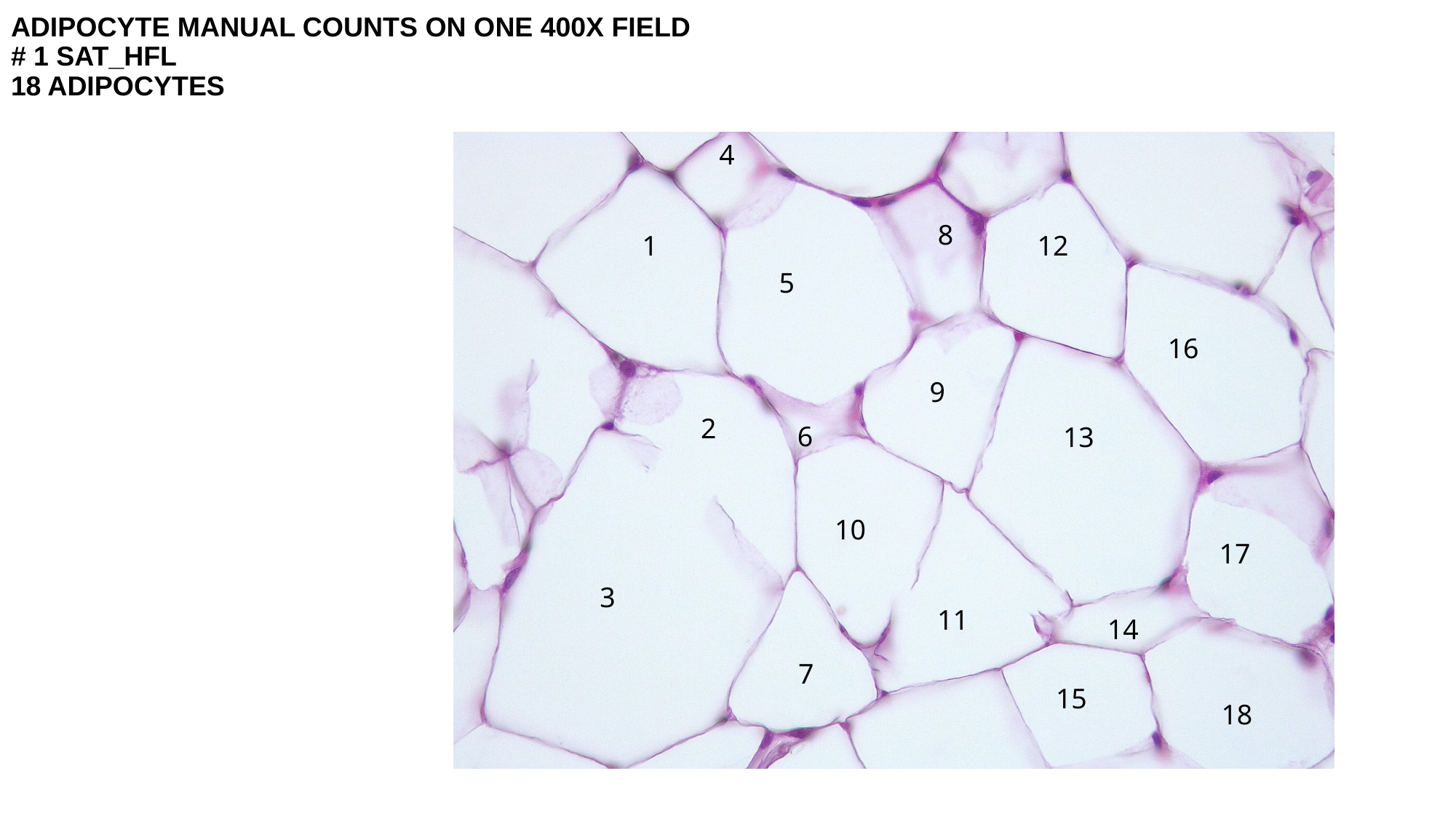

# ADIPOCYTE MANUAL COUNTS ON ONE 400X FIELD # 1 SAT_HFL 18 ADIPOCYTES
4
8
1
12
5
16
9
2
6
13
10
17
3
11
14
7
15
18

## Slide 2
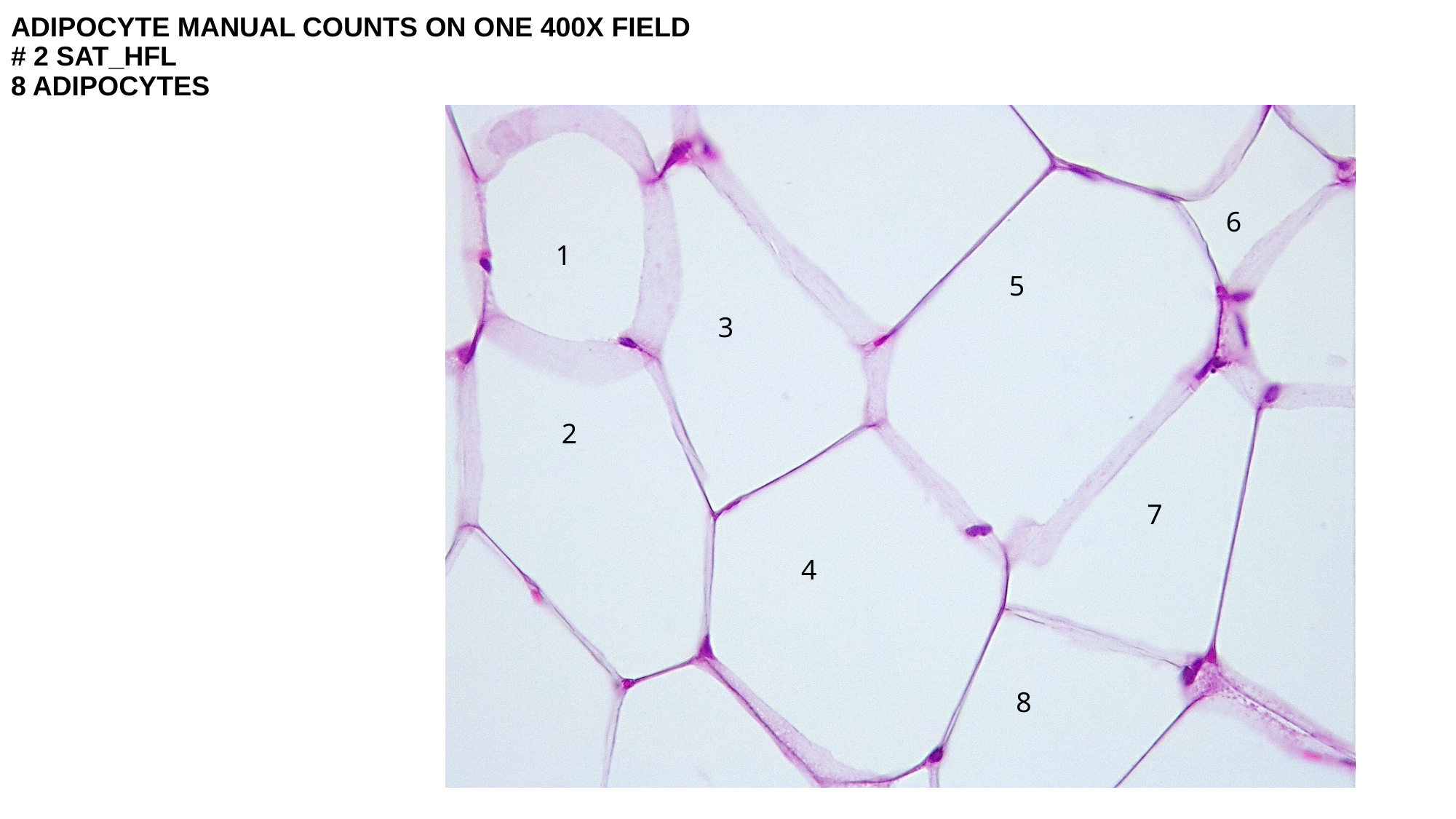

# ADIPOCYTE MANUAL COUNTS ON ONE 400X FIELD # 2 SAT_HFL 8 ADIPOCYTES
6
1
5
3
2
7
4
8

## Slide 3
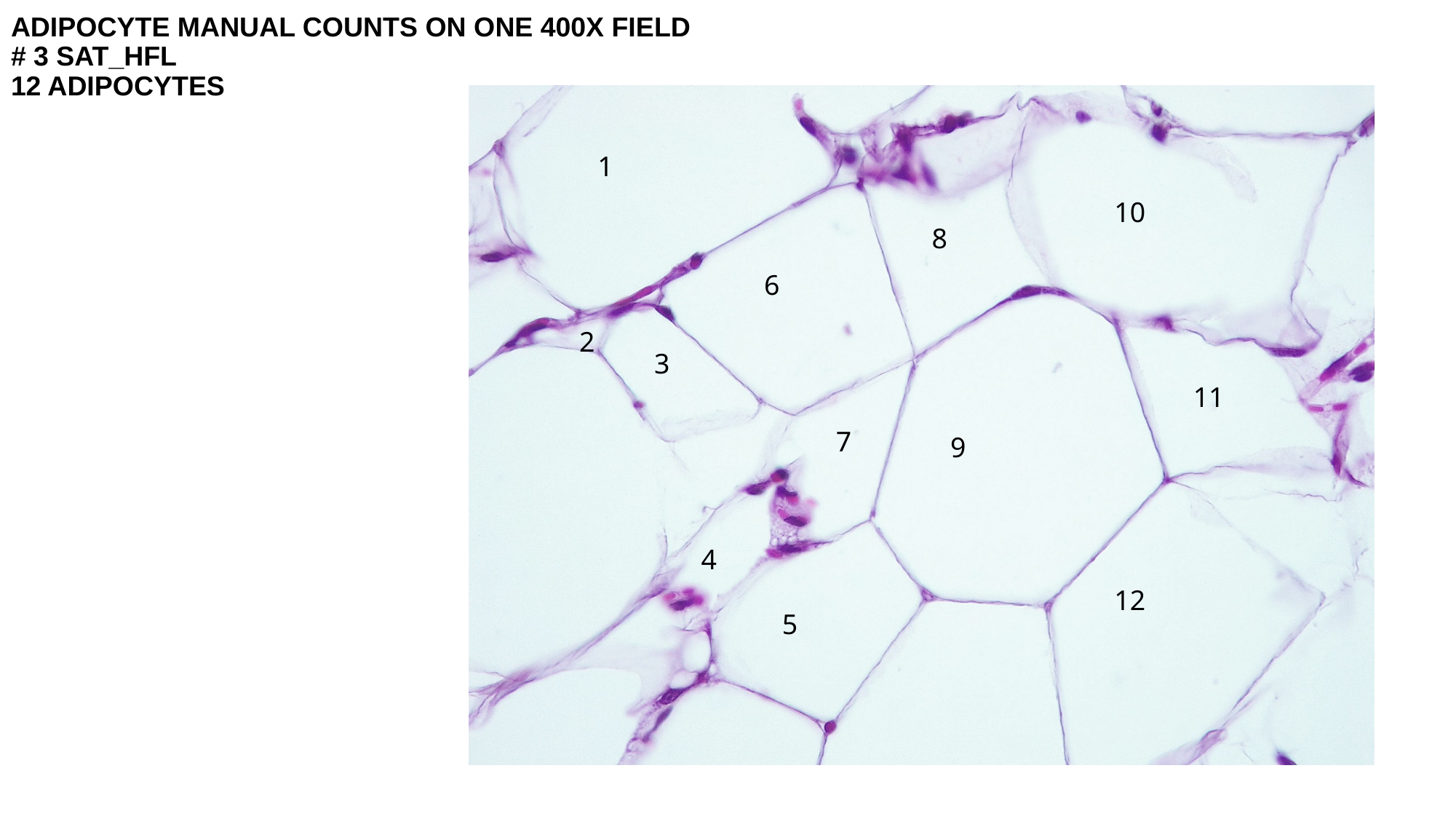

# ADIPOCYTE MANUAL COUNTS ON ONE 400X FIELD # 3 SAT_HFL 12 ADIPOCYTES
1
10
8
6
2
3
11
7
9
4
12
5

## Slide 4
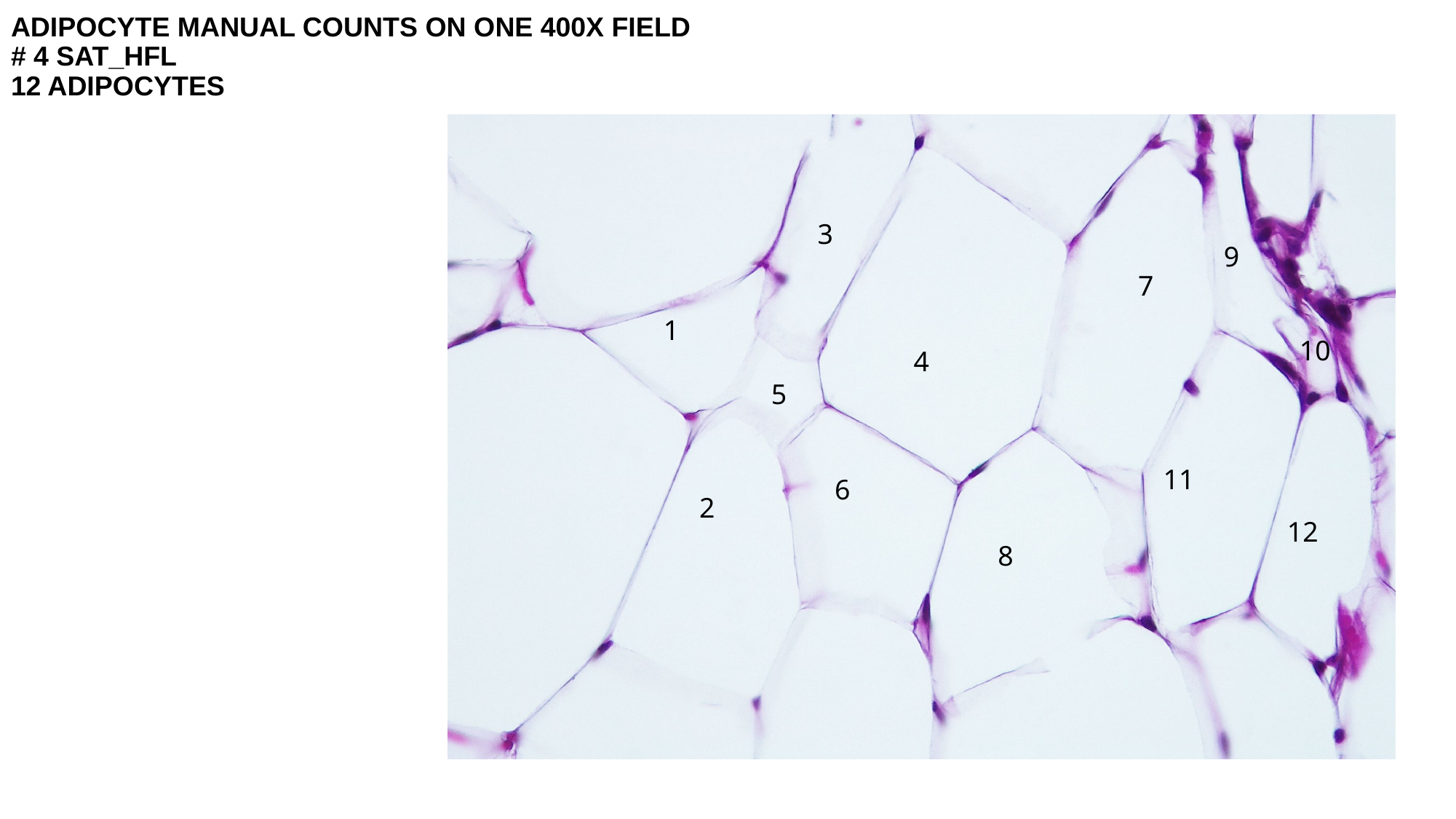

# ADIPOCYTE MANUAL COUNTS ON ONE 400X FIELD # 4 SAT_HFL 12 ADIPOCYTES
3
9
7
1
10
4
5
11
6
2
12
8

## Slide 5
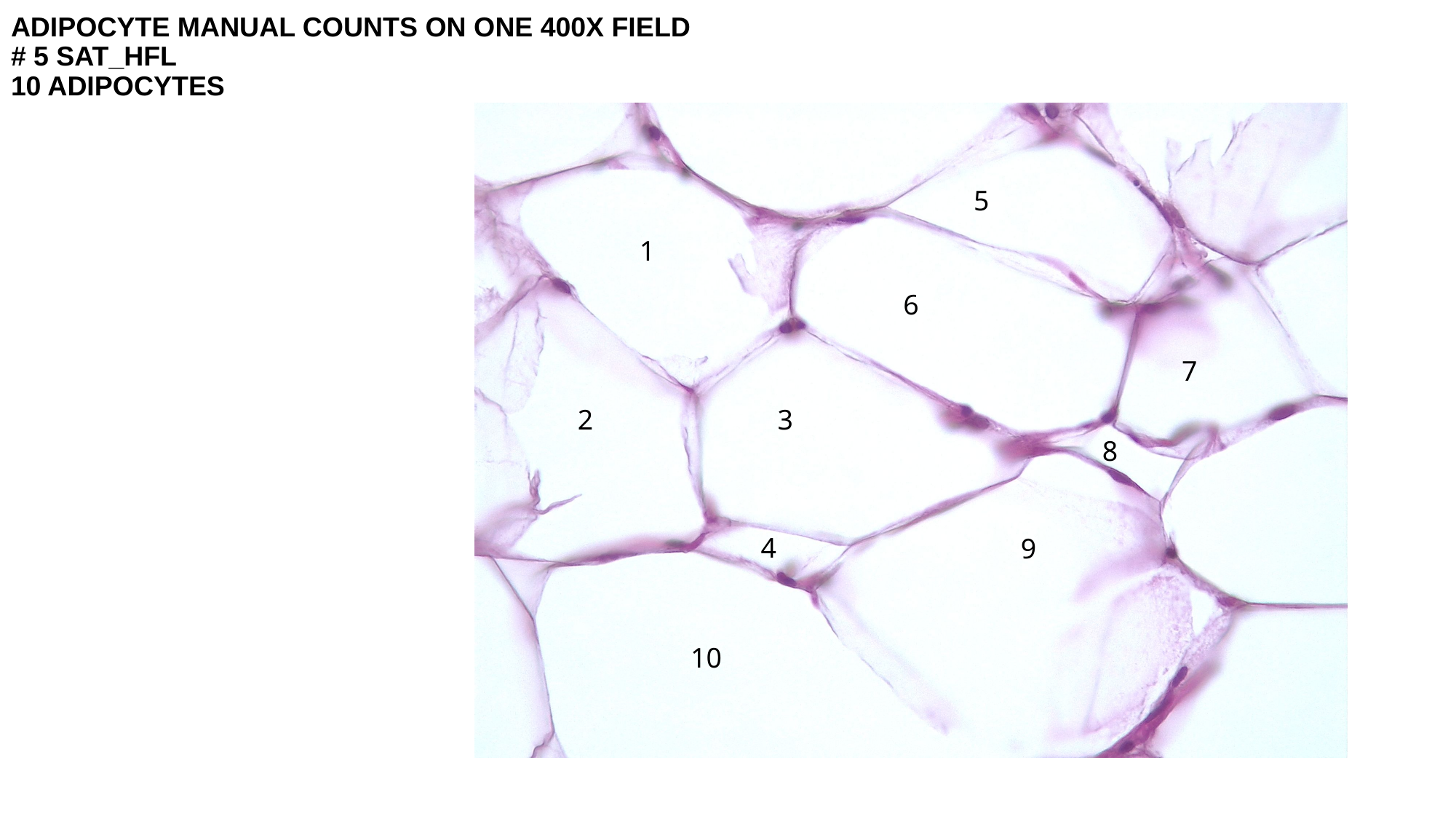

# ADIPOCYTE MANUAL COUNTS ON ONE 400X FIELD # 5 SAT_HFL 10 ADIPOCYTES
5
1
6
7
2
3
8
4
9
10

## Slide 6
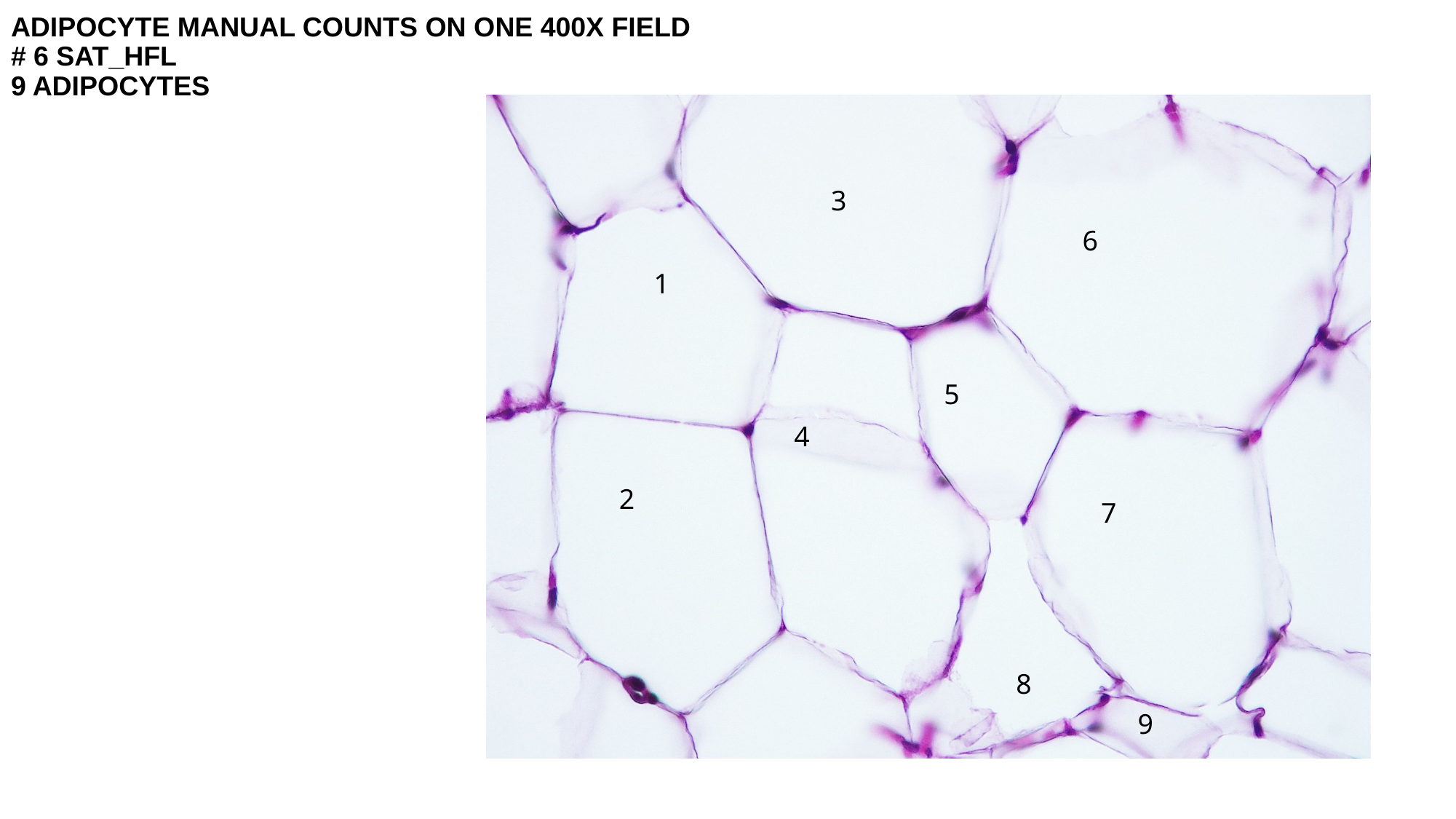

# ADIPOCYTE MANUAL COUNTS ON ONE 400X FIELD # 6 SAT_HFL 9 ADIPOCYTES
3
6
1
5
4
2
7
8
9

## Slide 7
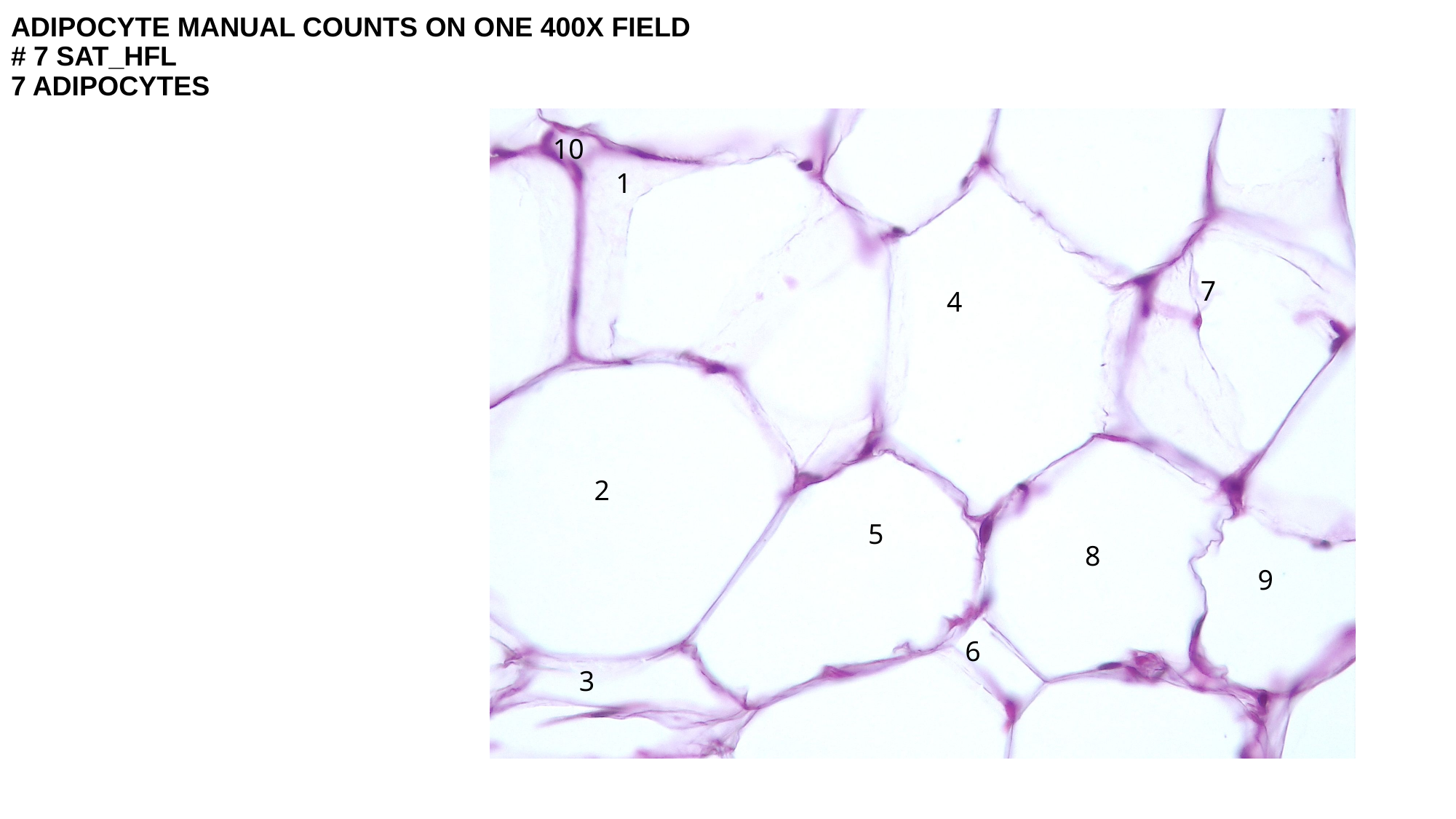

# ADIPOCYTE MANUAL COUNTS ON ONE 400X FIELD # 7 SAT_HFL 7 ADIPOCYTES
10
1
7
4
2
5
8
9
6
3

## Slide 8
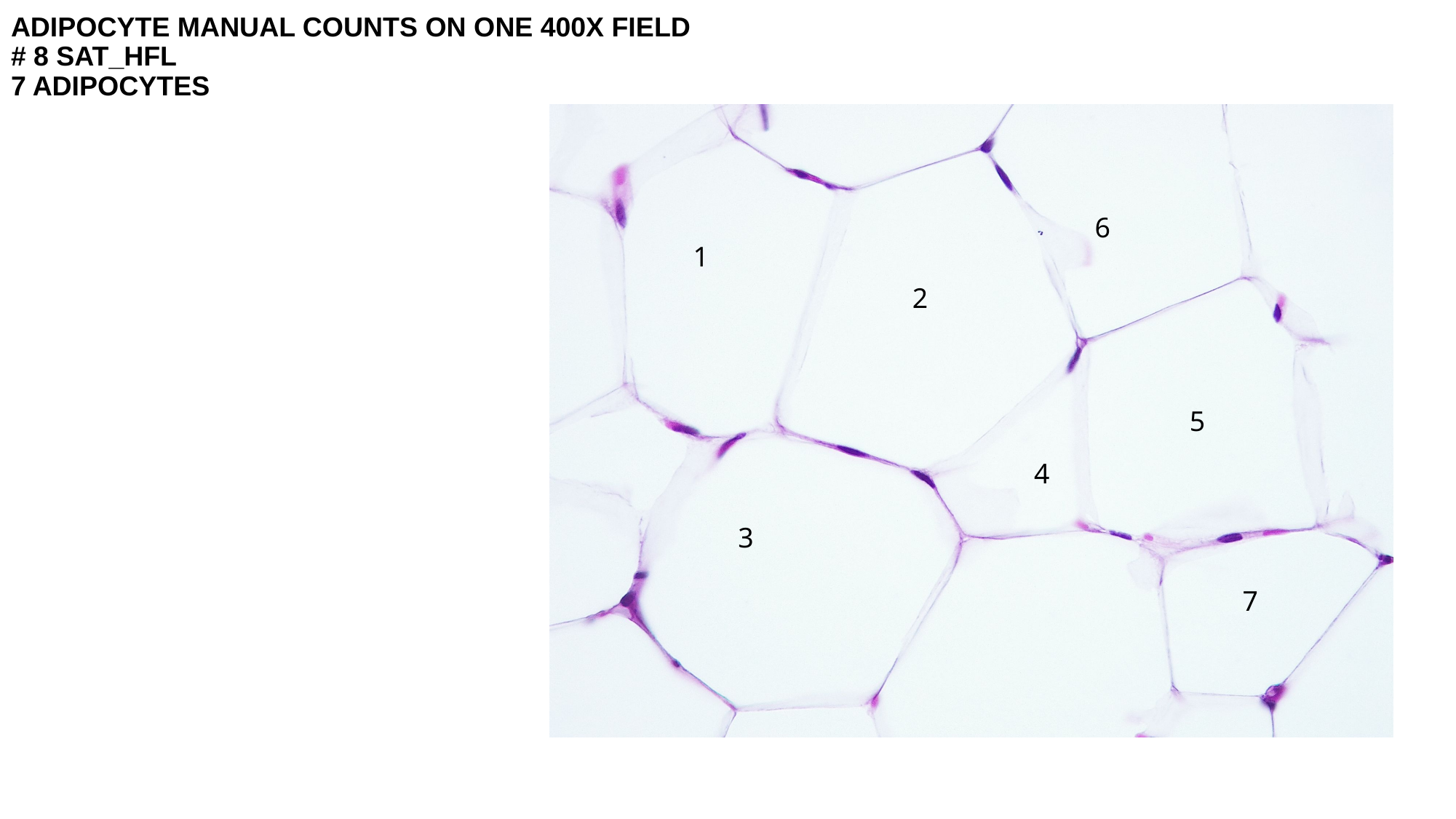

# ADIPOCYTE MANUAL COUNTS ON ONE 400X FIELD # 8 SAT_HFL 7 ADIPOCYTES
6
1
2
5
4
3
7
